# Single crystals of purely organic free-standing two-dimensional woven polymer networks

**DOI:** 10.1038/s41557-024-01580-3

**Published:** 2024-07-18

**Authors:** Ding Xiao, Zhitong Jin, Guan Sheng, Liya Chen, Xuedong Xiao, Tianyu Shan, Jiao Wang, Rahul Navik, Jianping Xu, Lin Zhou, Qing-Hui Guo, Guangfeng Li, Yihan Zhu, J. Fraser Stoddart, Feihe Huang

**Affiliations:** 1 Stoddart Institute of Molecular Science, Department of Chemistry, Zhejiang University, Hangzhou, P. R. China; 2 Zhejiang-Israel Joint Laboratory of Self-Assembling Functional Materials, ZJU-Hangzhou Global Scientific and Technological Innovation Center, Zhejiang University, Hangzhou, P. R. China; 3 School of Chemistry and Chemical Engineering, Frontiers Science Centre for Transformative Molecules, Shanghai Jiao Tong University, Shanghai, P. R. China; 4 Center for Electron Microscopy, Institute for Frontier and Interdisciplinary Sciences, State Key Laboratory Breeding Base of Green Chemistry Synthesis Technology and College of Chemical Engineering, Zhejiang University of Technology, Hangzhou, P. R. China; 5 Department of Chemistry, University of Hong Kong, Hong Kong, P. R. China; 6 Simpson Querrey Institute for BioNanotechnology, Northwestern University, Chicago, IL USA; 7 School of Chemistry, University of New South Wales, Sydney, New South Wales Australia

**Keywords:** Supramolecular polymers, Two-dimensional materials

## Abstract

The aesthetic and practicality of macroscopic fabrics continue to encourage chemists to weave molecules into interlaced patterns with the aim of providing emergent physical and chemical properties when compared with their starting materials. Weaving purely organic molecular threads into flawless two-dimensional patterns remains a formidable challenge, even though its feasibility has been proposed on several occasions. Herein we describe the synthesis of a flawless, purely organic, free-standing two-dimensional woven polymer network driven by dative B−N bonds. Single crystals of this woven polymer network were obtained and its well-defined woven topology was revealed by X-ray diffraction analysis. Free-standing two-dimensional monolayer nanosheets of the woven polymer network were exfoliated from the layered crystals using Scotch Magic Tape. The surface features of the nanosheets were investigated by integrated low-dose and cryogenic electron microscopy imaging techniques. These findings demonstrate the precise construction of purely organic woven polymer networks and highlight the unique opportunities for the application of woven topologies in two-dimensional organic materials.

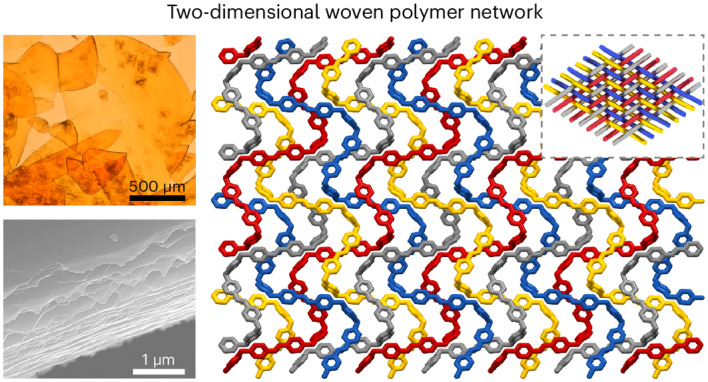

## Abstract (web summary)

Weaving purely organic molecular threads into two-dimensional patterns remains a formidable challenge. Now, driven by the formation of dative B–N bonds, a purely organic, two-dimensional and flawless woven polymer network has been prepared. In addition, free-standing monolayers of woven polymer nanosheets have been obtained through mechanical exfoliation.

## Main

Weaving, going back thousands of years^1,2^, has proven to be an enduring and powerful tool on the macroscopic and microscopic scales for the construction of functional two-dimensional (2D) and three-dimensional (3D) fabrics from relatively simple one-dimensional (1D) strands. From culture to engineering and from macroscopic to microscopic displays, this Stone Age artistry has been used and admired in everyday life, provoking creativity and ingenuity in both art and science. In a manner reminiscent of the making of macroscopic fabrics, molecular weaving demonstrates the possibilities for the bottom-up regulation of material properties as a consequence of the topological construction starting from molecular threads^3–18^. Hence, the nanoscale applications of weaving technology are considered to be promising strategies for the construction of versatile new materials for ever-expanding applications in modern society, while simultaneously arousing interest in the fields of chemistry and materials science^19–23^.

Two-dimensional materials have attracted considerable attention since the exfoliation of graphene from graphite in 2004 using a mechanical cleavage method^24^. Ultrathin sheet morphology and extremely high surface areas bring superior properties and promising applications in the fields of condensed matter physics, materials science and chemistry^25–37^. Inspired by the topology of weaving and encouraged by the emergent properties of 2D materials, plaiting molecular threads into 2D patterns has been a perennial pursuit for chemists^38^. In 2017, based on a sacrificial metal–organic framework template strategy, Wang and coworkers^12^ reported a 2D polymer network with interwoven chains. Thereafter, Wennermers et al.^19^ described a triaxial-patterned supramolecular woven network (molecular kagome). The periodically interlaced matrix was formed as a result of *π*···*π* stacking between pendent perylene monoimide units. In 2020, the Leigh group^20^ prepared layered 2D molecular woven fabrics by tessellating metal-coordinated woven 3 × 3 molecular grids into polymer networks using disulfide bonds. These groundbreaking investigations represent notable steps forward in the development of 2D woven materials^21^.

Weaving purely organic molecular threads into flawless 2D biaxial woven patterns, however, remains a significant challenge, although this possibility has been proposed on a number of occasions^19–23^. Furthermore, single crystals of purely organic, free-standing two-dimensional woven polymer networks (2DWPNs) have not been comprehensively investigated. As a result, obtaining precise structural information, such as bond lengths, bond angles and the spatial positions of atoms in polymer networks, has been difficult when discussing the mechanisms of formation and structure–property relationships in 2D woven materials. Moreover, free-standing 2D monolayered woven sheets exfoliated from layered materials are scarce^12,19,20^, posing an obstacle to the effective exploration of their diverse surfaces and structural features.

Herein we show how a purely organic 2DWPN was obtained and characterized by single-crystal X-ray analysis. Moreover, free-standing 2D monolayers were exfoliated successfully from bulk crystals using Scotch Magic Tape. Specifically, we obtained this 2DWPN by defining two-over and two-under weaving patterns based on the woven polymerization of 1,4-bis(benzodioxa-borole)benzene (**BDBB**) and 1,2-bis(4-pyridyl)ethylene (**BPE**) driven by dative B–N bonds. The key point about this approach is that the polymer network topology is regulated through the adjustable angles associated with the B–N bonds, which take advantage of the intrinsic conformational flexibility of these linkages to facilitate the formation of the desired woven topology^39,40^. In addition, benefiting from the highly dynamic nature of dative B–N bonds in solution and their high stability in the solid state, centimetre-scale single crystals of the 2DWPN were easy to obtain, laying a solid material foundation for the exploration of the formation mechanism, the 2D woven surface features and mechanical properties of the 2DWPN.

## Results and discussion

### Construction and characterization of 2DWPN-1

**Fig. 1:**
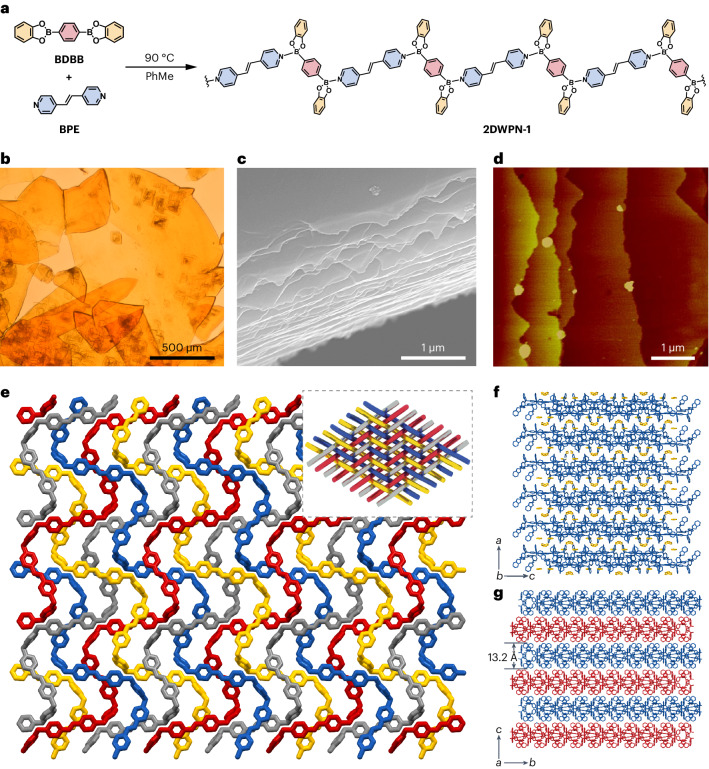
Illustration of the 2DWPN labelled 2DWPN-1. **a**, Synthesis of **2DWPN-1**. **b**, Optical microscopic images of its single crystals. **c**,**d**, Scanning electron microscopy (**c**) and AFM (**d**) images of **2DWPN-1** crystal cross-section. **e**, Top view of a monolayer of the crystal packing in **2DWPN-1**. The independent chains are distinguished by different colours. Solvent molecules, H atoms and catechol groups were removed for the sake of presenting a clearer woven display. The inset shows a graphical representation of the biaxial weaving structure in **2DWPN-1**. **f**, View along the *b* axis of the crystal packing in **2DWPN-1**; here PhMe molecules (yellow) were retained between layers and H atoms were removed for the sake of providing a clearer display. **g**, View along the *a* axis of the crystal packing in **2DWPN-1**; all solvent molecules and H atoms were removed for the sake of clarity.

**BDBB** was synthesized by employing dehydration between commercially available 1,4-phenylenediboronic acid and catechol. Subsequently, the 2DWPN labelled **2DWPN-1** was obtained by heating a colourless mixture of **BDBB** and commercially available **BPE** in methylbenzene (PhMe) at 90 °C for 2 h and slowly cooling the reaction mixture to room temperature (Fig. 1a and Supplementary Figs. 1–9). An optical microscopic image revealed that centimetre-scale yellow single crystals of **2DWPN-1** were obtained with a schistose structure (Fig. 1b).

The micromorphology of a **2DWPN-1** crystal was examined using field-emission scanning electron microscopy. The macroscopic schistose crystal is composed of molecule-thick sheets as a result of well-organized stacking (Fig. 1c). Furthermore, atomic force microscopy (AFM) was employed to illustrate the structure associated with the accumulation. We found that the thickness of each layer is approximately 1.3 nm (Supplementary Fig. 8) and the arrangement between layers is loose, a situation that favours mechanical exfoliation to obtain monolayers (Fig. 1d). In addition, single-crystal X-ray diffraction was employed to directly observe precise molecular structure information regarding **2DWPN-1**, which crystallizes in the orthorhombic *P*_*bca*_ space group with lattice constants *a* = 23.4671(6), *b* = 19.6536(5) and *c* = 28.7688(5) Å (Supplementary Table 1). Further analysis revealed that **BDBB** molecules are linked by dative B–N bonds to **BPE** molecules to form 1D helical chains.

Density functional theory (DFT) calculations based on the crystal data for **2DWPN-1** were used to analyse the nature of the dative B–N bonds. The large electron localization function value between the B and N atoms indicates (Supplementary Figs. 10–12) the formation of dative B–N bonds with strong bond energies of 150.7 kJ mol^–1^ (Supplementary Table 7). The dative B–N bonds provide excellent stability to **2DWPN-1** and serve as the foundation for its ability to be exfoliated and applied in some scenarios. From the top view of the crystal structure (*c* axis), a 2D polymer network with a two-over and two-under weaving pattern is generated through the entanglement of the 1D helical threads. For clarity, hydrogen atoms and catechol groups, which do not affect the main chain topology, are concealed. Distinct colours were assigned to molecular chains, facilitating an intuitive representation of the intricate woven topology within the crystal structure of **2DWPN-1** (Fig. 1e and Supplementary Fig. 13). Notably, the woven nodes in the 2D networks are formed (Supplementary Fig. 14) by the crossover of a pair of double bonds in the **BPE** moieties in the molecular warp and weft, with a plane-to-plane distance of 3.79 Å and a cross angle of 42° between two **BPE** moieties. These covalent entanglements exert the influence of the woven topology upon the mechanical properties of these self-standing networks. Such a 2D woven monolayer can stack to form a final 3D multilayer structure, although the filling of PhMe solvent molecules between these woven layers prevents them from being densely packed (Fig. 1f). Furthermore, given their distribution between layers, these solvent molecules have no impact on the freedom of movement associated with chain segments within the woven layers. Importantly, when the solvent is removed at 120 °C, the stacking superstructure is disrupted, causing the crystals to lose their crystallinity (Supplementary Fig. 15). A side-on view (*bc* planes; Fig. 1g) reveals an anti-direction arrangement mode between adjacent layers in which the thickness of the 2D woven monolayer is 13.2 Å from direct measurement of the single-crystal structure, a distance that is consistent with the observed results from AFM measurements.

### Mechanism of topology formation

**Fig. 2:**
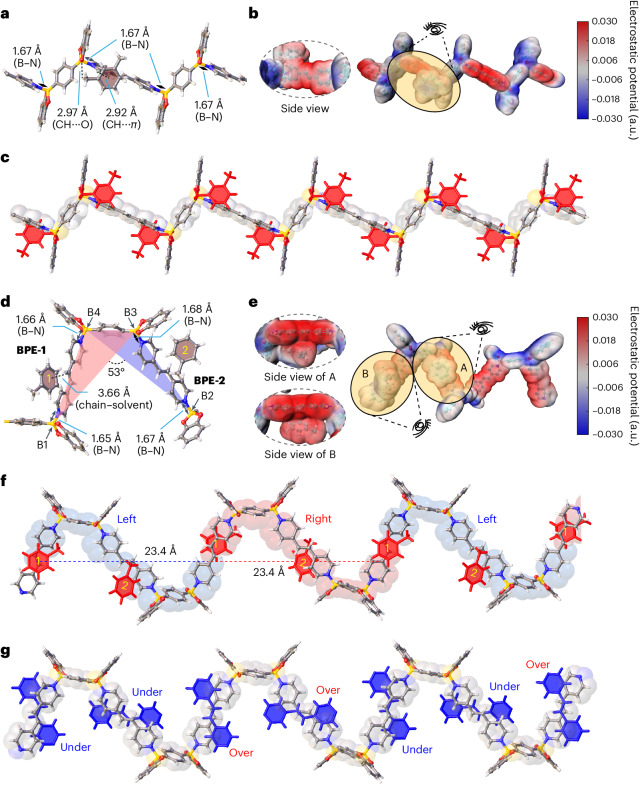
Mechanism of topology formation in NWPN-1 and 2DWPN-1. **a**, Illustration of the interactions between the solvent molecules and the polymer chains in **NWPN-1**, and the lengths of the dative B–N bonds in **NWPN-1**. **b**, Surface electrostatic potentials of **NWPN-1**. **c**, The conformation of a polymer chain in **NWPN-1**. **d**, The interaction between solvent molecules (**Toluene-1** and **Toluene-2**) and molecular chains, the lengths of dative B–N bonds and the dihedral angle in a unit of **2DWPN-1**. **e**, Surface electrostatic potentials of **2DWPN-1**. **f**, The helical conformation of the polymer chains in **2DWPN-1**. **g**, The woven nodes of the polymer chains in **2DWPN-1**.

To gain a deeper insight into the mechanism of formation of the woven topology in the case of this polymer network, we synthesized, as a means of comparison (Supplementary Figs. 16–21), a non-woven polymer network (**NWPN-1**) in *m*-xylene from the same starting materials and used the same method of preparation as that used in the production of **2DWPN-1**. In essence, the synthetic conditions employed in making **2DWPN-1** and **NWPN-1** differ only in the nature of the solvent, suggesting that it plays a key role in the formation of these polymer topologies. Single-crystal X-ray diffraction analysis showed that each asymmetric unit of **NWPN-1** contains only one *m*-xylene molecule. We also investigated the interactions between the solvent molecules and the polymer network, showing (Supplementary Fig. 22) the existence of C–H···*π* interactions (3.26−3.27 Å) between a *m*-xylene molecule and the four catechol groups located on different polymer main chains. The conformation of each polymer chain (Fig. 2a) is not affected by the solvent molecules in **NWPN-1**, and each dative B–N bond in the chains has the same bond length of 1.67 Å. We can see clearly (Fig. 2b) that the symmetrical distribution of electron clouds and solvent molecules has no effect on the electron cloud distribution associated with the polymer chains, by inspection of the electrostatic potential map. Therefore, the polymer chains in **NWPN-1** exhibit a classical extended zigzag conformation (Fig. 2c). Finally, the zigzag polymer chains of **NWPN-1** adopt a grillage accumulation as a consequence of the balanced interactions of each *m*-xylene residue with its four adjacent polymer chains (Supplementary Figs. 23 and 24).

In the case of **2DWPN-1** obtained in PhMe, an asymmetric unit consists of two **BDBB** moieties, two **BPE** moieties and two PhMe molecules (**Toluene-1** and **Toluene-2**). In the formation of the 2D woven topology, the two PhMe molecules are thought to play different roles. Specifically, **Toluene-1** forms a *π*···*π* stacking interaction (3.66 Å) with **BPE-1** (Fig. 2d). As a result, the bond energies of the two related dative B–N bonds in **BPE-1**, with bond lengths of 1.65 and 1.66 Å, are enhanced on account of their higher electron densities. The interaction between **Toluene-2** and **BPE-2** is weaker. Accordingly, the two related dative B–N bonds possess longer bond lengths of 1.68 and 1.67 Å. Based on electrostatic potential analysis, **Toluene-1** enters into space conjugation with **BPE-1**, resulting in the enhancement of the electron cloud density around **BPE-1**. On the contrary, **Toluene-2** has no obvious space conjugation with **BPE-2** and the electron cloud around **BPE-2** exhibits no obvious change (Fig. 2e). These asymmetric interactions result in a dihedral angle of 53° between the B1–B4–B3 and B2–B3–B4 planes related to two **BPE** units and one **BDBB** moiety during the polymerization (Fig. 2d). The repeated extension of the twisted fragments results in helical polymer chains (Fig. 2f). Interestingly, each helical chain is composed of two helical segments with the same helical pitch of 23.4 Å that are arranged alternately in opposite helical directions.

The adjacent reverse spirals make up two alternating bays with up and down openings, providing the over-and-under stereoscopic space for the weaving of warps and wefts (Supplementary Figs. 25–30) guided by the *π*···*π* stacking interactions between the **BPE** moieties in different threads. Each bay contains two **BPE** moieties, allowing two polymer chains to cross by an over-or-under space to form a typical biaxial woven topology (Fig. 2g). Therefore, the PhMe molecules change the conformation of the polymer chains to form a 2DWPN as a result of the *π*···*π* stacking interactions with **BPE** moieties, while the steric *m*-xylene residues are unable to do so. We suspect that the construction of the woven topology is influenced strongly by the steric effect of benzene solvent molecules. To verify this hypothesis, we prepared single crystals of the dative B–N polymer in *p*-xylene and the more sterically bulkly *o*-xylene using the same method as that employed in the production of **2DWPN-1** and **NWPN-1**, respectively. In a manner similar to the preparation of **2DWPN-1** and **NWPN-1**, a 2DWPN (**2DWPN-2**) was generated in *p*-xylene by *π*···*π* interactions between *p*-xylene residues and **BPE** moieties, and a parallel stacking non-woven polymer (**NWPN-2**) was obtained in *o*-xylene (Supplementary Figs. 31–56), an observation that is consistent with the expected result. Overall, the conformation of the dative B–N bond is impacted by the steric and electronic properties of the interacting components^41,42^. This kind of intrinsic conformational flexibility is the central element for the acquisition of the woven topology in the presence of selected solvents.

### Preparation and characterization of 2DWPN-1 nanosheets

From the packing of **2DWPN-1** (Fig. 1g), the adjacent layers interact only through solvent molecules. We adopted a computational method to analyse the difference between the interactions of adjacent layers and the interactions among different components inside the same layer in the crystal structure of **2DWPN-1**. Theoretical calculations showed that the packing energy (Δ*E*_*ab*_) of **2DWPN-1** along the *ab* plane is 4.5 times stronger than that (Δ*E*_*c*_) along the *c* axis (Supplementary Fig. 57). The much stronger interactions among different components in the same 2D, *ab* plane, compared with the interactions between 2D layers along the *c* axis, make the layered **2DWPN-1** crystals easy to exfoliate into large-size crystalline woven monolayers (Supplementary Figs. 58–66).

**Fig. 3:**
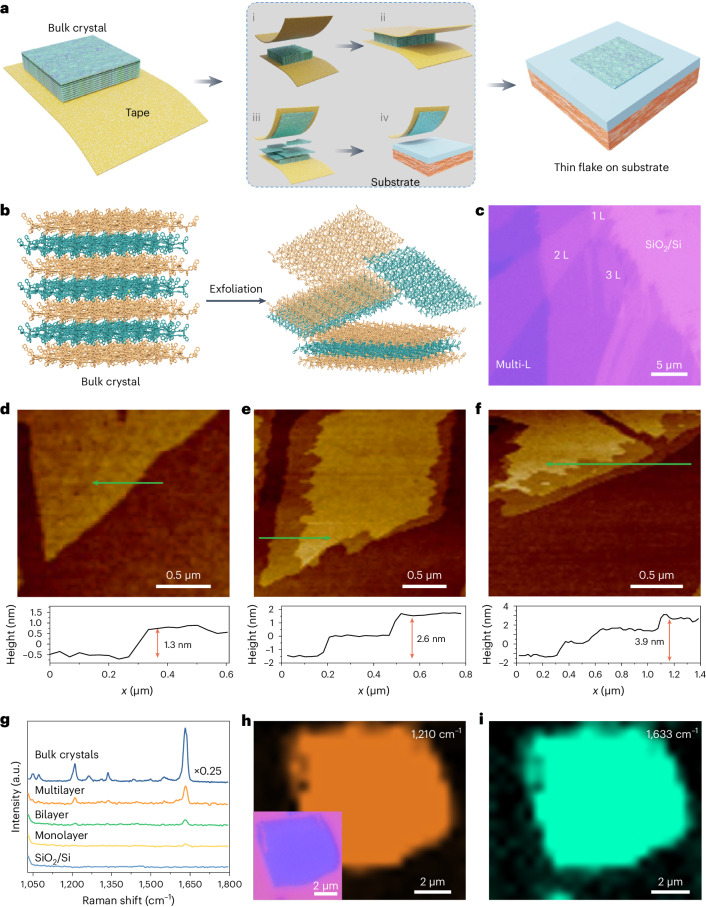
Fabrication and characterization of atomically thin 2DWPN-1 flakes. **a**, Schematic diagram of Scotch Magic Tape-assisted mechanical exfoliation and transfer of a layered **2DWPN-1** bulk crystal. **b**, Schematic illustration of the exfoliation of **2DWPN-1** nanosheets. **c**, Optical microscopic image of exfoliated monolayer (1 L), bilayer (2 L), trilayer (3 L) and multilayer (Multi-L) **2DWPN-1** flakes on a SiO_2_/Si substrate. **d**–**f**, AFM images of monolayer (**d**), bilayer (**e**) and trilayer (**f**) **2DWPN-1** flakes on a SiO_2_/Si substrate and corresponding thicknesses (bottom) of 1.3 nm, 2.6 nm and 3.9 nm, respectively. The thickness plots are along the green arrows. **g**, Raman spectra of bulk **2DWPN-1** and **2DWPN-1** flakes. **h**,**i**, The corresponding Raman mapping images based on the intensities at 1,210 cm^−1^ (**h**) and 1,633 cm^−1^ (**i**) of a **2DWPN-1** flake. The inset shows the corresponding optical microscopic image. Source data

Inspired by the weak interlayer interactions in the layered **2DWPN-1** crystals, we exfoliated a bulk crystal using a Scotch Magic Tape-assisted mechanical method^43^, with reference to the exfoliation (Fig. 3a,b) of traditional van der Waals 2D materials. Micrometre-scale **2DWPN-1** flakes were obtained on a SiO_2_/Si substrate (Fig. 3c). Notably, we obtained a free-standing monolayer woven flake of **2DWPN-1** with a thickness of 1.3 nm, and introduced the woven polymer network materials successfully to the 2D limit (Fig. 3d). The AFM images of a bilayer woven flake with a thickness of 2.6 nm and a trilayer woven flake with a thickness of 3.9 nm are shown in Fig. 3e,f, respectively. Moreover, the root-mean-square roughness (*R*_q_) values of these **2DWPN-1** flakes (Supplementary Fig. 64) were less than 0.2 nm, indicating atomically flat surfaces. Compared to the 2D woven nanosheets obtained by the liquid exfoliation method (Supplementary Figs. 64 and 65), the clean and atomically flat surfaces of these as-prepared **2DWPN-1** flakes are more favourable for exploring intrinsic properties. As a result, the layered **2DWPN-1** crystal was exfoliated successfully to the molecular thickness limit. The **2DWPN-1** flakes have a micrometre-scale edge size and atomically flat surfaces, paving the way for applications of woven polymer networks in the field of 2D materials.

Raman spectroscopy was performed to obtain structural and phonon-vibration information for **2DWPN-1**. Raman spectra (Fig. 3g) of a bulk **2DWPN-1** crystal show two main peaks at 1,210 and 1,633 cm^−1^, and a few weaker peaks at 1,050, 1,072, 1,264, 1,336 and 1,552 cm^−1^. The analysis, combined with the structure of **2DWPN-1**, indicates that the Raman peaks at 1,050 and 1,072 cm^−1^ are attributable to the dative B–N vibrations, and the signals at 1,336, 1,633 and 1,552 cm^−1^ arise from C=C vibrations. The peaks at 1,210 and 1,264 cm^−1^ correspond to the C–O and C–B vibrations, respectively. The experimental data match well with the calculated results (Supplementary Figs. 67–70). Without an obvious peak position shift, the Raman peak intensities gradually decrease with the thickness of **2DWPN-1** decreasing to the 2D limit. The Raman mapping images based on the intensity at 1,210 cm^−1^ (Fig. 3h) and 1,633 cm^−1^ (Fig. 3i) of a **2DWPN-1** flake (inset of Fig. 3h) show homogeneous colour distribution, demonstrating the uniform and high-quality 2D sheets of **2DWPN-1**.

We also performed transmission electron microscopy (TEM) to investigate the real-space superstructure information of **2DWPN-1** nanosheets. From low-magnification TEM images (Supplementary Fig. 71), we observed that the **2DWPN-1** crystals have a nanoplate morphology with homogeneous distributions of B, C, N and O as probed by electron energy-loss spectroscopy (Supplementary Figs. 72 and 73). The selected-area electron diffraction patterns of **2DWPN-1** nanosheets match well with the simulated electron diffraction patterns based on bulk single-crystal structures (Supplementary Fig. 71), confirming that the 2D woven nanosheets retain the periodic structures of the *ab* plane in the 3D crystals. The explicit molecular-level real-space structural elucidation of the purely organic networks requires high-resolution (HR) TEM imaging. Organic materials are extremely vulnerable to electron-beam irradiation and are subject to radiolytic structural damage under the high accumulated electron dose required for traditional HRTEM imaging. Recent advances in low-dose electron microscopy allow the direct imaging of beam-sensitive materials such as metal–organic frameworks^44–47^. Nevertheless, the low-dose imaging of purely organic networks has greater challenges on account of the low image contrast in addition to the high beam sensitivity of the networks.

**Fig. 4:**
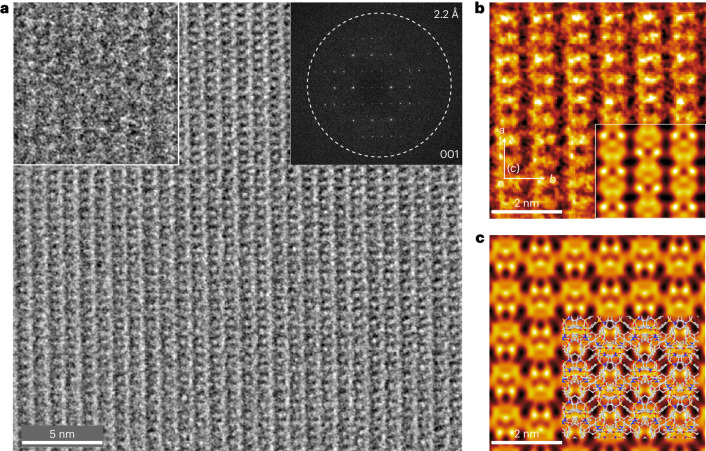
Cryogenic low-dose HRTEM image of the crystalline 2DWPN-1. **a**, Cryogenic low-dose HRTEM image of the crystalline **2DWPN-1** taken along the [001] direction. The image was denoised and the contrast inversion effects caused by the objective lens CTF were corrected. Insets show a raw image (upper left) and the fast Fourier transform pattern (upper right) of the HRTEM image with structural information transfer up to 2.2 Å. **b**, A false-colour motif averaged along the [010] direction. **c**, The simulated projected electrostatic potential with a point spread function width of 2.2 Å. The inset of **b** shows the *p2gm* projection symmetry-imposed image, and the projected structural model of **2DWPN-1** is embedded in **c**.

It has been widely acknowledged that electron microscopy carried out under cryogenic conditions (that is, cryo-EM) can minimize radiolytic beam damage effects and thus alleviate the dose-limited resolution problem^48–51^. To this end, by integrating low-dose and cryo-EM imaging techniques together with a state-of-the-art electron direct detector and a custom-designed ultrastable cryo-transfer holder, we were able to image the **2DWPN-1** nanosheets with high spatial resolution and superstructure integrity. A low-dose cryo-EM image of the woven polymer networks projected along the [001] direction of **2DWPN-1** crystals is shown in Fig. 4a. From the denoised image after correcting for contrast inversion effects arising from the contrast-transfer function (CTF) of the objective lens, the topological features of the woven polymer networks can be visualized clearly with structural information transfer up to 2.2 Å. From the false-colour motif-averaged and symmetry-imposed images in Fig. 4b, the woven polymer networks exhibit contrast closely resembling interconnected hollow ovals that match well with the projected electrostatic potentials simulated based on the proposed structural model for **2DWPN-1** (Fig. 4c). These observations confirm unambiguously the identical polymer chain conformations and associate the woven topologies of **2DWPN-1** with those determined by X-ray crystallography.

### Mechanical properties of 2DWPN crystals

**Fig. 5:**
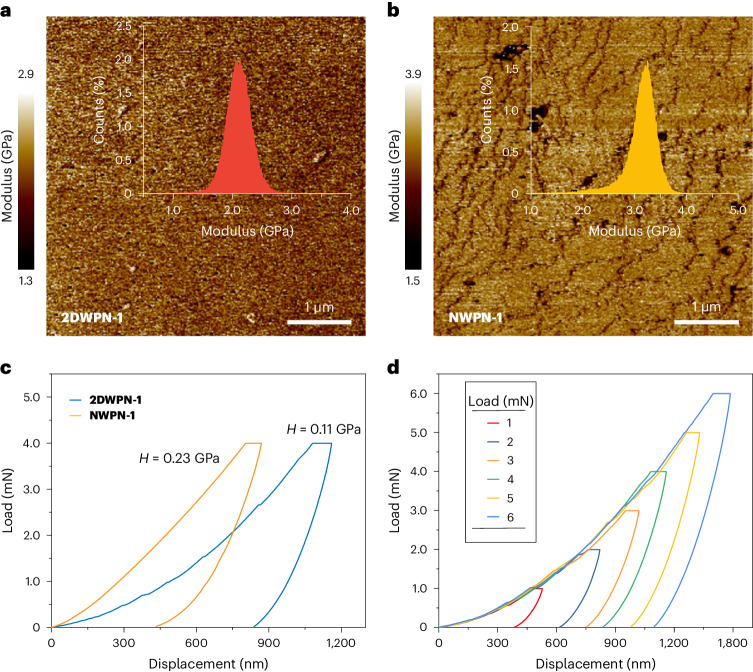
Study of the mechanical properties of 2DWPN-1 and NWPN-1. **a**,**b**, Mapping of the Derjaguin–Müller–Toporov moduli of **2DWPN-1** (**a**) and **NWPN-1** (**b**). The insets show the corresponding Derjaguin–Müller–Toporov modulus distribution. **c**, *P*–*h* curves for **2DWPN-1** on the (001) facet, and **NWPN-1** on the (110) facet of the multilayer stacking plane at a fixed load (4 mN). **d**, *P*–*h* curves obtained using various loadings from 1 to 6 mN. Source data

Apart from their aesthetically appealing superstructures, the woven topology also furnishes materials with unique mechanical behaviour. To reveal quantitatively the influence of the woven structure on the mechanical response of **2DWPN-1** crystals, PeakForce quantitative nanomechanical mapping and nanoindentation were employed. For a comparison, we also investigated the mechanical properties of the **NWPN-1** crystals. Here **2DWPN-1** and **NWPN-1** differ only in their polymer topologies, so the differences in their mechanical properties reflect the influence of their woven topologies. The uniform surface textures (Fig. 5a,b) of crystals of **2DWPN-1** and **NWPN-1** ensure a homogeneous modulus distribution. The Derjaguin–Müller–Toporov moduli are 2.1 and 3.2 GPa for **2DWPN-1** and **NWPN-1**, respectively. Therefore, the surface flexibility of **2DWPN-1** with the woven topology is superior to that of non-woven **NWPN-1**.

Nanoindentation was employed to investigate the deformation properties of **2DWPN-1** and **NWPN-1**. Load–displacement (*P*–*h*) curves were obtained (Fig. 5c) by indenting crystals of **2DWPN-1** or **NWPN-1** with a Berkovich tip in a load-controlled mode of 4 mN. Under the same load, **2DWPN-1** (*D* = 1,159 nm, *H* = 0.11 GPa) exhibits a greater displacement depth (*D*) and a lower hardness value (*H*) than **NWPN-1** (*D* = 868 nm, *H* = 0.23 GPa) along the *c* axis, which is perpendicular to the multilayer stacking plane of the crystal structures. Moreover, load–displacement (*P*–*h*) curves with similar slopes obtained by indenting **2DWPN-1** and **NWPN-1** crystals (Fig. 5d and Supplementary Figs. 74–76) confirm the good homogeneity of these crystals using a load-controlled mode of different loads from 1 to 6 mN. Overall, the quantitative nanomechanical mapping and nanoindentation results both show that the woven topology endows the rigid crystal with excellent flexibility, an observation that may be attributed to the unique and effective mechanisms to disperse external forces as a result of the conformational freedom of polymer chains and sliding space in the topological networks. In particular, the molecular warp and weft strands experience relative sliding through woven nodes with an interplanar distance of 3.79 Å under external stimuli while the interlaced structures of the polymer chains maintain the integrity of woven networks (Supplementary Fig. 14). As a result, the stress is dispersed throughout the polymer network, preventing stress concentration that could lead to structural damage while imparting exceptional flexibility upon the crystal (Supplementary Videos 1 and 2). These findings highlight that topological control is a simple and effective method to develop crystalline materials with exotic mechanical performance.

## Conclusion

Herein we have designed and constructed a class of purely organic 2DWPNs based on woven polymerization driven by dative B−N bonds. Because of the intrinsic conformational flexibility of dative B−N linkages, the solvent molecules play a crucial role in the formation of the woven topology, as verified through X-ray single-crystal analysis and comparison tests. Furthermore, free-standing 2D nanosheets with a thickness of 1.3 nm were successfully prepared from as-synthesized 2D woven polymer crystals by using micromechanical exfoliation, adding a long-awaited member to the 2D materials family. Subsequently, the surface structural features of these 2D woven polymer crystals were shown at a molecular level by low-dose cryogenic TEM. In addition, the woven topology provides a unique and effective stress-dispersion pathway and improves the flexibility of rigid crystals. This investigation not only presents an efficient approach to pure organic woven polymer networks as structural curiosities, but also unravels the mechanism of their formation, in addition to the 2D features and mechanical properties of woven polymer networks, opening the door to functional materials constructed from purely organic free-standing 2DWPNs.

## Methods

### HRTEM imaging

The microscope was operated at an acceleration voltage of 300 kV under a very low electron dose rate (0.5 e^−^ pixel^–1^ s^−1^ or 3 e^−^ Å^−2^ s^−1^; e^–^, electron). Images were acquired as a series of 100 frames with a 0.2 s per frame rate and the image stack was subjected to post-acquisition drift correction and contrast correction before comparing it with the simulated projected electrostatic potentials. A motif-averaging algorithm^44,45^ was used for denoising a unit cell or multiple unit cells by averaging over the crystalline domains with periodic units. After correcting for contrast inversion effects arising from the CTF, the motif-averaged image was visualized using a ‘black-body’ false-colour code. Furthermore, projection symmetry imposition allows the correction of minor misalignments of the zone axis during imaging based on a lattice averaging algorithm^52^.

The Fourier transform *I*(**q**) of a HRTEM image is related to the spatial frequency (**q**)-dependent structure factor *F*(**q**) by the CTF of the objective lens *T*(**q**):

1 $$I({\mathbf{q}})=T({\mathbf{q}})\times F({\mathbf{q}}).$$

The CTF can be expressed as two parts, a sine function sin*χ*(**q**) and an envelope function *D*(**q**):

2 $$T({\mathbf{q}})=\sin \left(\uppi \Delta f\lambda {{\mathbf{q}}}^{2}+\frac{1}{2}\uppi {C}_{{\mathrm{s}}}{\lambda }^{3}{{\mathbf{q}}}^{4}\right)D\left({\mathbf{q}}\right)$$

where Δ*f* is defocus, *λ* is the electron wavelength and *C*_s_ is spherical aberration. For the low-dose imaging of low-contrast organic crystalline matters using an aberration-corrected electron microscope, as conducted in this work, most types of aberrations are minimal and the CTF is modulated deliberately by introducing an appropriate amount of defocus to enhance the contrast and thus improve the resolution, limited by the noise. Because of the contrast reversal arising from the sine function part of the CTF, the HRTEM image is usually not directly interpretable. Following a CTF-correction procedure reported previously (*C*_s_ = 1 μm and Δ*f* = 330 nm at 300 kV)^45^, we were able to flip the sign of the inverted parts of the CTF curves to make the HRTEM image more chemically interpretable.

### Bond energy calculations

All-electron DFT calculations were carried out employing the latest version of ORCA quantum chemistry software (version 5.0.3)^53^. The calculated structures were based on their single-crystal structures. The positions of the hydrogen atoms were optimized, and the other atoms retained their positions unchanged. The B3LYP functional^54^ and def2-SVP basis set^55^ were adopted for the DFT calculations. The DFT-D3 with Becke–Johnson damping^56^ was applied to correct the weak interactions and improve the accuracy of the calculations. The nature of the bonds and the hole–electron analysis were studied using Multiwfn software^57^. Blue and yellow isosurfaces represented hole and electron distributions, respectively. The bond energy (BE) between **BDBB** and **BPE** was calculated based on the following formula:

3 $${\rm{BE}}={E}_{{\rm{adduct}}}-\left({E}_{{\rm{BDBB}}}+{E}_{{\rm{BPE}}}\right) $$

where *E*_adduct_ is the energy of entire molecule consisting of **BPE** and **BDBB**, *E*_BDBB_ is the energy of fragment **BDBB** and *E*_BPE_ is the energy of fragment **BPE**. Based on these methods, the value of the B–N bond energy was calculated. Specifically, we selected a part of the polymer chain as the adduct from its crystal data, and split it from the B−N bond into two parts, **BDBB** and **BPE**. Following DFT calculations, the corresponding optimized conformations, bond energies and bond lengths (crystal data) were found and are listed in Supplementary Table 7.

### Electrostatic potential map calculations

The calculated structures were built from their single-crystal structures. The positions of the hydrogen atoms were optimized, while the other atoms retained their positions unchanged. All-electron DFT calculations were carried out using the latest version of ORCA quantum chemistry software^53^ (version 5.0.4). The BLYP functional was adopted for geometry optimization calculations, the def2-SVP basis set^55^ was used and the optimal geometry for each compound was determined. The singlet point energy calculations were performed with the B3LYP functional and a larger basis set, the def2-TZVP basis set. The SMD implicit solvation model^58^ was used to account for the solvation effect. The DFT-D3 dispersion correction with Becke–Johnson damping^56,59^ was applied to correct the weak interactions and improve the calculation accuracy. Electrostatic potential analysis was performed by Multiwfn software^57^. The visualization of the electrostatic potential analysis was achieved using VMD software.

## Online content

Any methods, additional references, Nature Portfolio reporting summaries, source data, extended data, supplementary information, acknowledgements, peer review information; details of author contributions and competing interests; and statements of data and code availability are available at 10.1038/s41557-024-01580-3.

## Supplementary information

Supplementary Information Supplementary Figs. 1–80, Tables 1–7 and Discussion.

Supplementary Video 1 In situ nanoindentation test for 2D woven polymer single crystal.

Supplementary Video 2 In situ nanoindentation test for non-woven polymer single crystal.

Supplementary Data 1 Crystallographic data for **2DWPN-1**; CCDC number 2238613.

Supplementary Data 2 Crystallographic data for **2DWPN-2**; CCDC number 2238614.

Supplementary Data 3 Crystallographic data for **NWPN-1**; CCDC number 2238615.

Supplementary Data 4 Crystallographic data for **NWPN-2**; CCDC number 2238616.

Supplementary Data 5 Computational data for electrostatic potential calculations.

Supplementary Data 6 Source data for supplementary figures.

## Source data

Source Data Fig. 3 Data for thicknesses of **2DWPN-1** nanosheet in Fig. 3d–f, and data for Raman spectra of bulk **2DWPN-1** and **2DWPN-1** flakes.

Source Data Fig. 5 Data for Derjaguin–Müller–Toporov modulus distribution (shown in Fig. 5a,b), and data for *P*–*h* curves (shown in Fig. 5c,d).

## Data Availability

Data supporting the findings of this investigation are available from the manuscript and its Supplementary Information. Crystallographic data for the structures reported have been deposited at the Cambridge Crystallographic Data Centre (CCDC) under deposition numbers 2238613 (**2DWPN-1**), 2238614 (**2DWPN-2**), 2238615 (**NWPN-1**) and 2238616 (**NWPN-2**). Copies of the data can be obtained free of charge via https://www.ccdc.cam.ac.uk/structures/. Supplementary videos are provided with this paper. Source data are provided with this paper.
