# Supplementary material for: Single crystals of purely organic free-standing two-dimensional woven polymer networks
Source: Nat Chem. 2024 Jul 18;16(11):1906–14. doi: 10.1038/s41557-024-01580-3 (PMC11527790; doi:10.1038/s41557-024-01580-3)
Supplement: Supplementary file 1 — Supplementary Figs. 1–80, Tables 1–7 and Discussion. [file 41557_2024_1580_MOESM1_ESM.pdf]

nature chemistry

Supplementary information https://doi.org/10.1038/s41557-024-01580-3

Single crystals of purely organic free-standing two-dimensional woven polymer networks

In the format provided by the authors and unedited

Table of Contents

*1. Synthesis and characterization of **BDBB*** 3

*2. Preparation of **2DWPN-1*** 4

*3. Preparation of **NWPN-1*** 4

*4. Preparation of **2DWPN-2*** 4

*5. Preparation of **NWPN-2*** 4

*6. Characterization of **2DWPN-1** crystals* 5

*7. AFM Image of a **2DWPN-1** bulk crystal* 10

*8. Color-filled maps of valence electron density and electron localization function (ELF) of **BPE*** 11

*9. Color-filled maps of valence electron density and ELF of **BDBB*** 12

*10. Color-filled maps of valence electron density and ELF of the dative compounds formed by **BDBB** and **BPE*** 13

*11. Crystal structure of **2DWPN-1*** 14

*12. Typical [π···π] interactions between polymer chains in the crystal structure of **2DWPN-1*** 14

*13. The effect of solvents on the crystallinity of crystal **2DWPN-1*** 15

*14. Characterization of **NWPN-1** crystals* 16

*15. Crystal structure of **NWPN-1*** 19

*16. Crystal structure of **2DWPN-1*** 21

*17. Characterization of **2DWPN-2** crystals* 24

*18. Crystal structure of **2DWPN-2*** 28

*19. Characterization of **NWPN-2** crystals* 35

*20. Crystal structure of **NWPN-2*** 38

*21. Theoretical calculations of packing energies for **2DWPN-1*** 41

*22. Optical microscopy images of typical **2DWPN-1** flakes exfoliated on a SiO_2/Si substrate*......42

23. AFM Images of 2D flakes on a SiO_2/Si substrate......44

24. AFM Measurements of blank SiO_2/Si substrates......47

*25. AFM of as-exfoliated **2DWPN-1** nanosheets obtained by liquid exfoliation*......48

*26. AFM Measurements of the particles obtained from **NWPN-1** bulk crystals after ultrasonication*......50

*27. Simulation of Raman peaks of **2DWPN-1***......51

*28. TEM Analysis of **2DWPN-1***......55

*29. Nanoindentation studies on crystals of **2DWPN-1** and **NWPN-1***......57

Supplementary Table 1|** Crystallographic data and structural refinement summary of **2DWPN-1......61

Supplementary Table 2|** Crystallographic data and structural refinement summary of **NWPN-1......62

Supplementary Table 3|** Crystallographic data and structural refinement summary of **2DWPN-2......63

Supplementary Table 4|** Crystallographic data and structural refinement summary of **NWPN-2......64

Supplementary Table 5|** Elastic moduli and hardness of **2DWPN-1......66

Supplementary Table 6|** Elastic moduli and hardness of **NWPN-1......66

***Supplementary Table 7|** Calculated results of the dative B−N bond energy*......67

Supplementary References......67

1. *Synthesis and characterization of* ***BDBB***

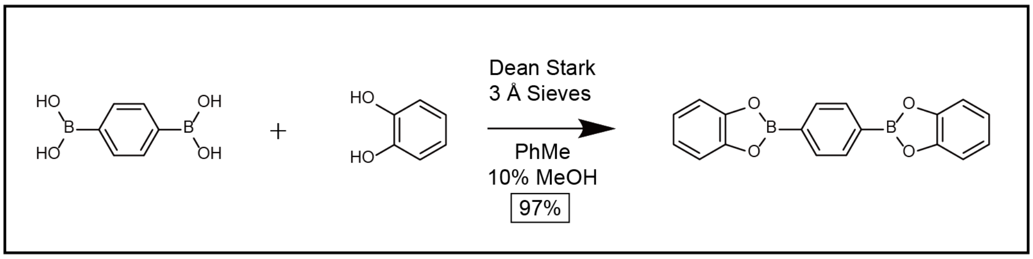

Supplementary Fig. 1. Synthetic route to **BDBB**

BDBB was synthesized as reported[1] in the literature. A mixture of benzene-1,4-diboronic acid (0.101 g, 0.610 mmol) and catechol (0.140 g, 1.27 mmol) was dissolved in PhMe (40.0 mL) and MeOH (4.0 mL). The solution was heated under reflux for 90 min with a Dean-Stark trap filled half full with 3 Å sieves. During this time a white solid began to precipitate from the solution. The solution was evaporated to dryness and excess of catechol was removed by sublimation using a Kugel-Rohr at 80 °C under reduced pressure (1 mm Hg). The resulting product took the form of white needle-like crystals (0.186 g, 97% yield). ^{1}H NMR (600 MHz, $CDCl_3$) δ 8.21 (s, 4H), 7.35–7.34 (dd, 4H, J = 5.76, 3.36 Hz), 7.16–7.15 (dd, 4H, J = 5.76, 3.30 Hz).

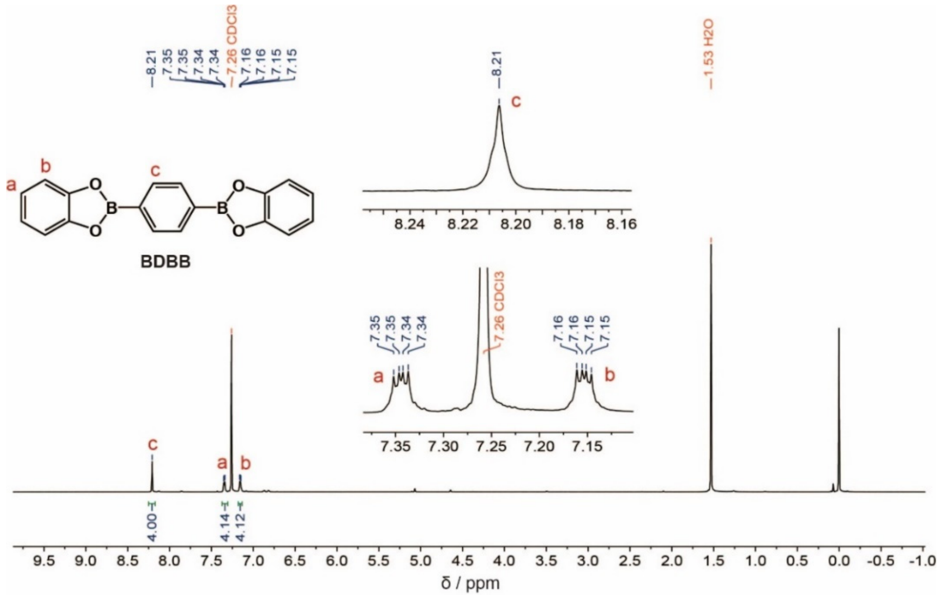

Supplementary Fig. 2. The ^{1}H NMR spectrum of **BDBB** in $CDCl_3$

2. *Preparation of* ***2DWPN-1***

BDBB (9.42 mg, 0.03 mmol) and **BPE** (5.47 mg, 0.03 mmol) were added to PhMe (15.0 mL) and mixed using ultrasound for 15 mins. The mixture was heated at 90 °C for 24 h. The resulting solution was cooled slowly to room temperature. Yellow single crystals of **2DWPN-1** of centimeter size were obtained.

3. *Preparation of* ***NWPN-1***

BDBB (9.42 mg, 0.03 mmol) and **BPE** (5.47 mg, 0.03 mmol) were added to *meta*-xylene (15.0 mL) and mixed using ultrasound for 15 mins. The mixture was heated at 90 °C for 24 h. The resulting solution was cooled slowly to room temperature. Yellow single crystals of **NWPN-1** were obtained.

4. *Preparation of* ***2DWPN-2***

BDBB (9.42 mg, 0.03 mmol) and **BPE** (5.47 mg, 0.03 mmol) were added into a solution of *para*-xylene (15.0 mL) and subjected to ultrasound for a duration of 15 minutes. Subsequently, the mixture was subjected to heating at 90 °C for 24 hours. The resultant solution was gradually cooled to ambient temperature. Yellow single crystals of **2DWPN-2** were obtained.

5. *Preparation of* ***NWPN-2***

BDBB (9.42 mg, 0.03 mmol) and **BPE** (5.47 mg, 0.03 mmol) were introduced into a 15.0 mL solution of *ortho*-xylene and exposed to ultrasonic treatment for 15 minutes. Following this, the mixture underwent heating at 90 °C for 24 hours. Afterward, the resulting solution was slowly cooled to room temperature, yielding yellow single crystals of **NWPN-2**.

6. *Characterization of **2DWPN-1** crystals*

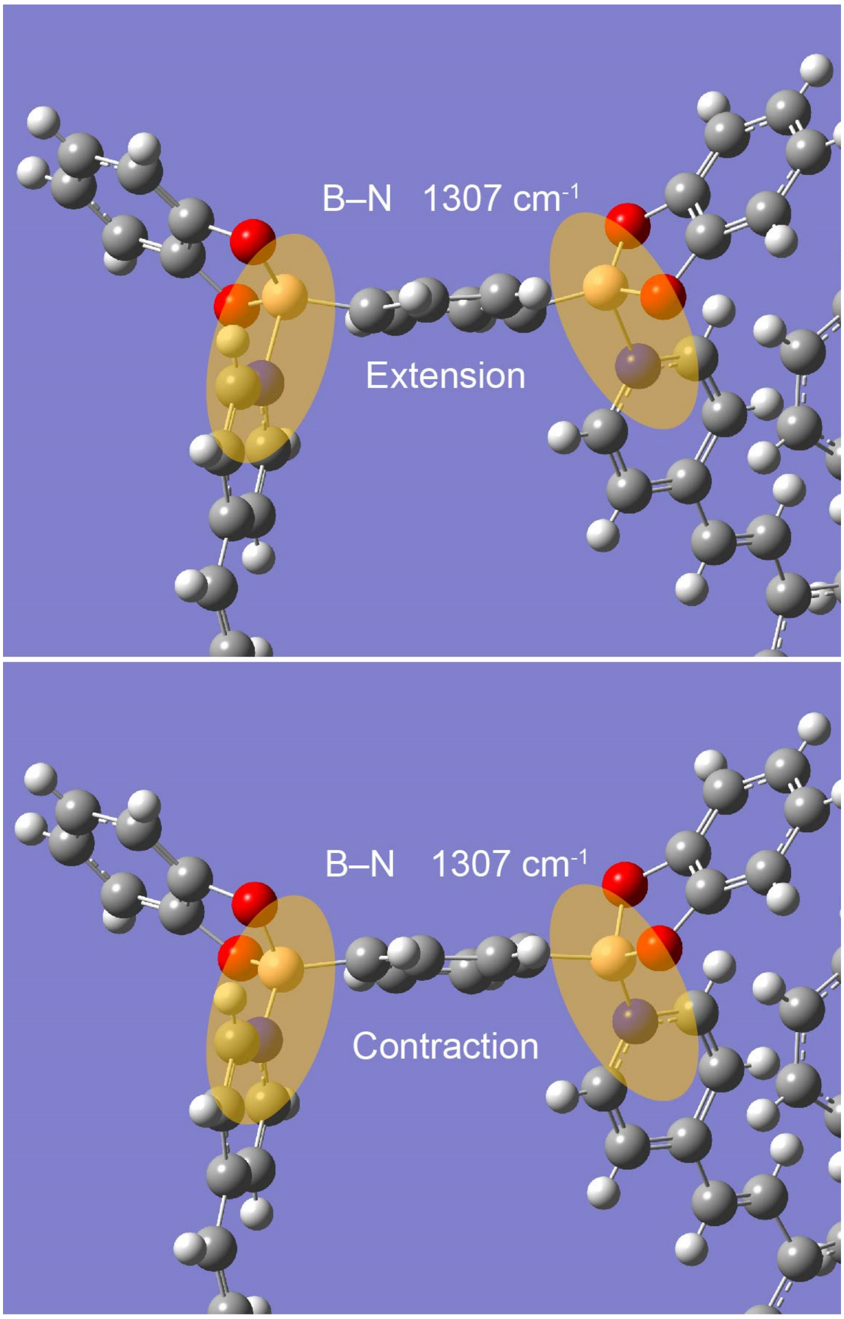

Supplementary Fig. 3. Simulation data of the infrared peak of dative B−N bonds from DFT calculation. The calculations are performed using the Gaussian 09 software package, employing the B3LYP /6-311G (d, p) level of theory[2].

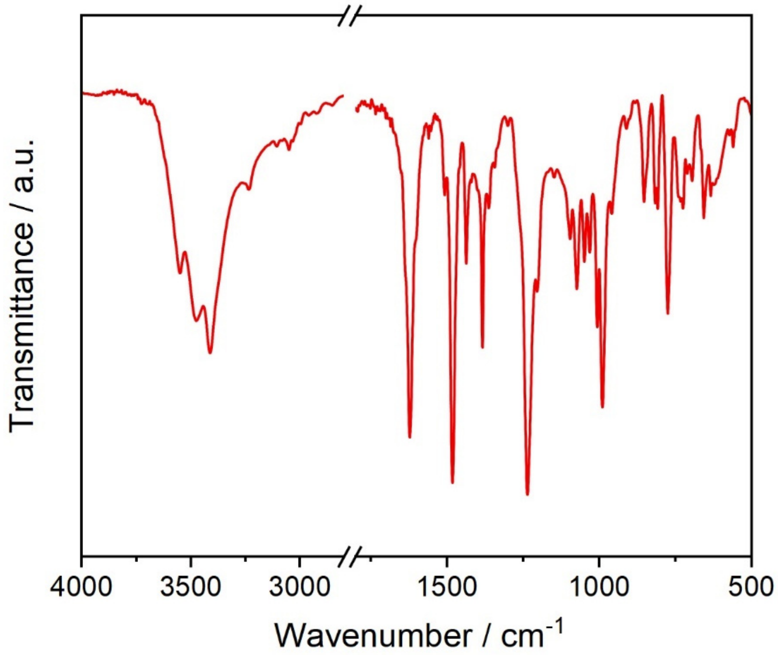

Supplementary Fig. 4. Infrared spectra of **2DWPN-1**. Wavenumbers 1483 cm^{-1} and 1237 cm^{-1} correspond to the characteristic absorption peaks of the B−O bond, and 1361 cm^{-1} corresponds to the characteristic absorption peak of the dative B−N bond

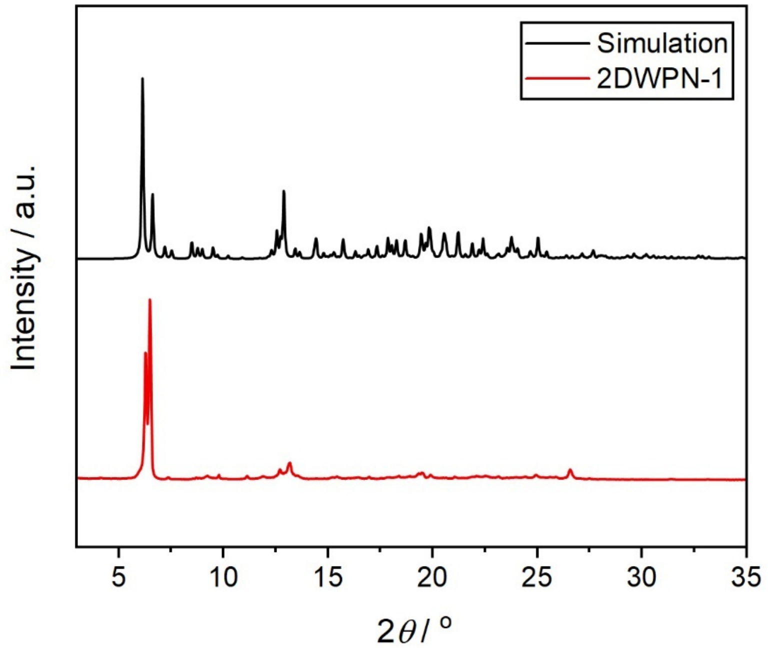

Supplementary Fig. 5. The PXRD patterns of **2DWPN-1** crystals. The PXRD pattern shows a sharp and strong peak of **2DWPN-1**, indicating a highly oriented crystal structure. The experiment data match well with the PXRD pattern simulated from the single crystal diffraction data.

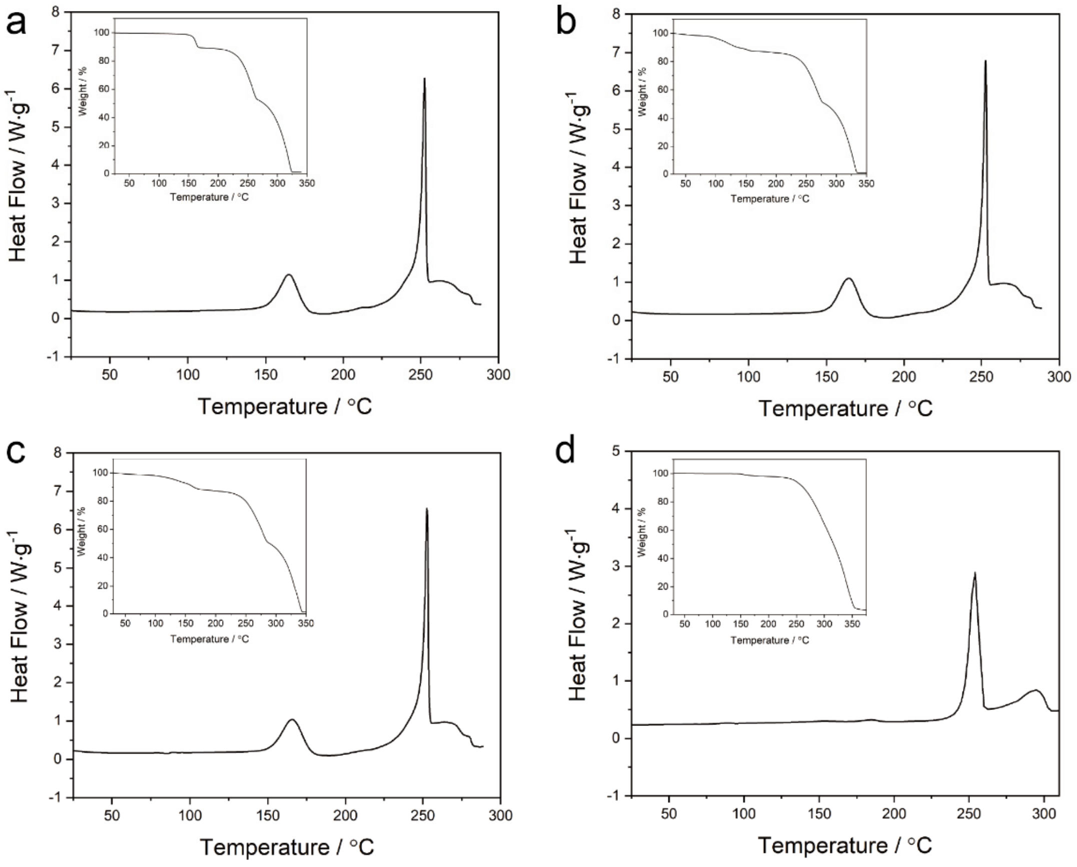

Supplementary Fig. 6. DSC thermograms of (**a**) newly prepared woven crystals of **2DWPN-1**. (**b**) Single crystals stored in air for 6 months. (**c**) Single crystals under vacuum at room temperature for 12 hours. (**d**) A sample under vacuum at 120 °C for 12 hours. Insets are the corresponding TGA curves.

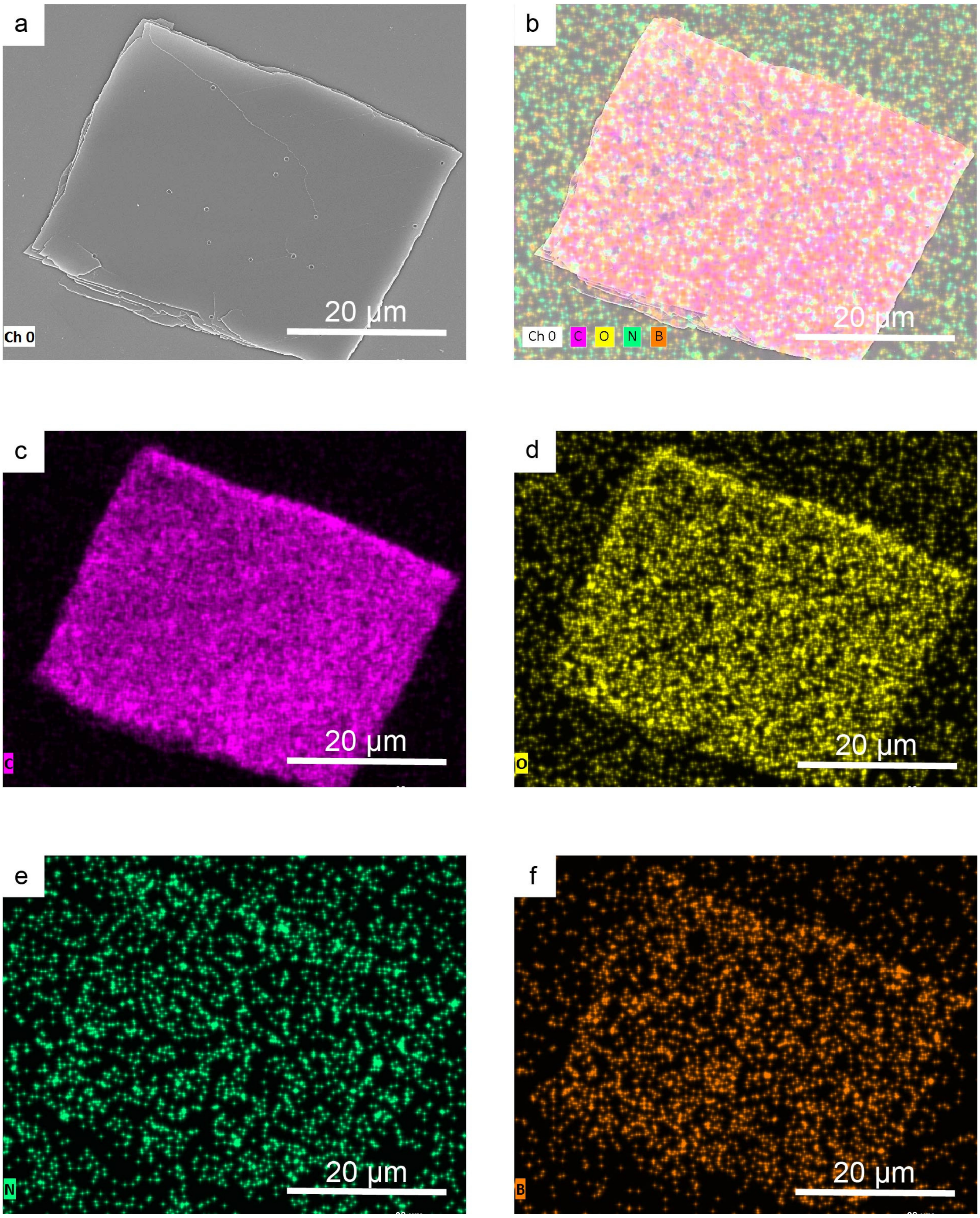

Supplementary Fig. 7. SEM Image (**a**) and the energy dispersive spectroscopy (EDS) mapping (**b**) of **2DWPN-1**. The corresponding elemental maps: carbon (**c**), oxygen (**d**), nitrogen (**e**), and boron (**f**)

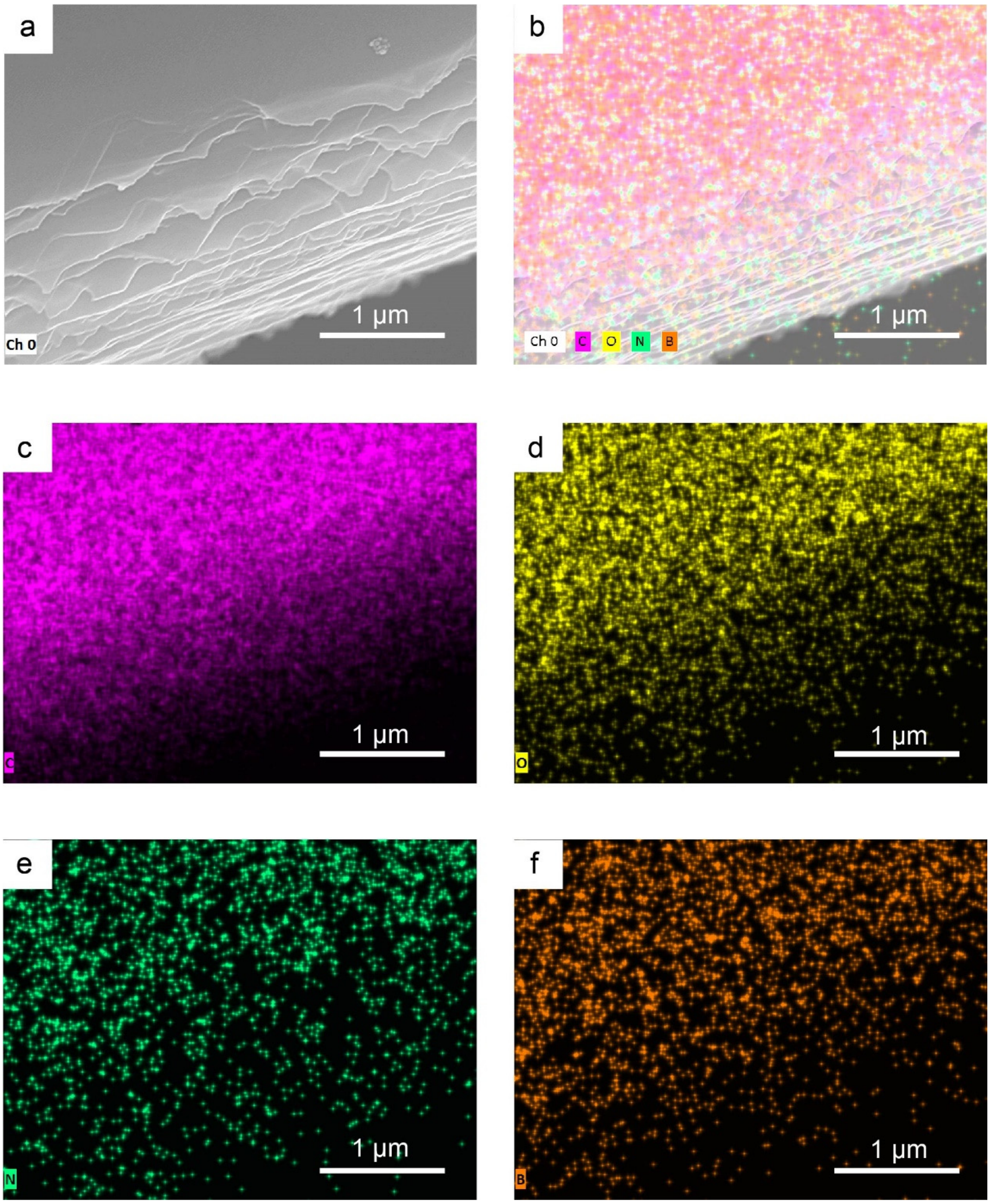

Supplementary Fig. 8. SEM Image (**a**) and the EDS mapping (**b**) of the cross section of **2DWPN-1**. The corresponding elemental maps: carbon (**c**), oxygen (**d**), nitrogen (**e**), and boron (**f**)

7. *AFM Image of a* ***2DWPN-1*** *bulk crystal*

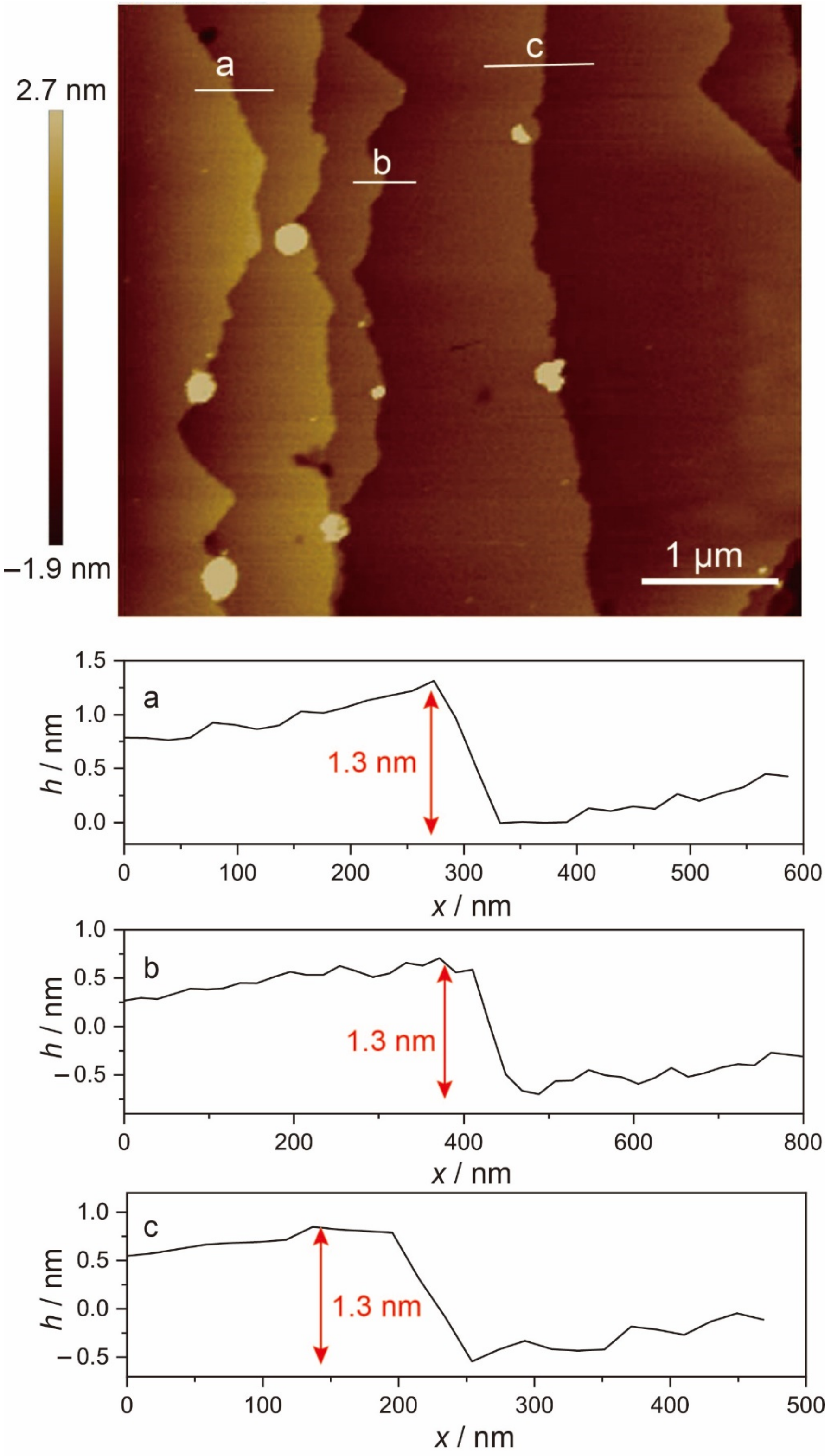

Supplementary Fig. 9. AFM Image of a **2DWPN-1** bulk crystal and the thickness of the corresponding monolayers

8. *Color-filled maps of valence electron density and electron localization function (ELF) of* ***BPE***

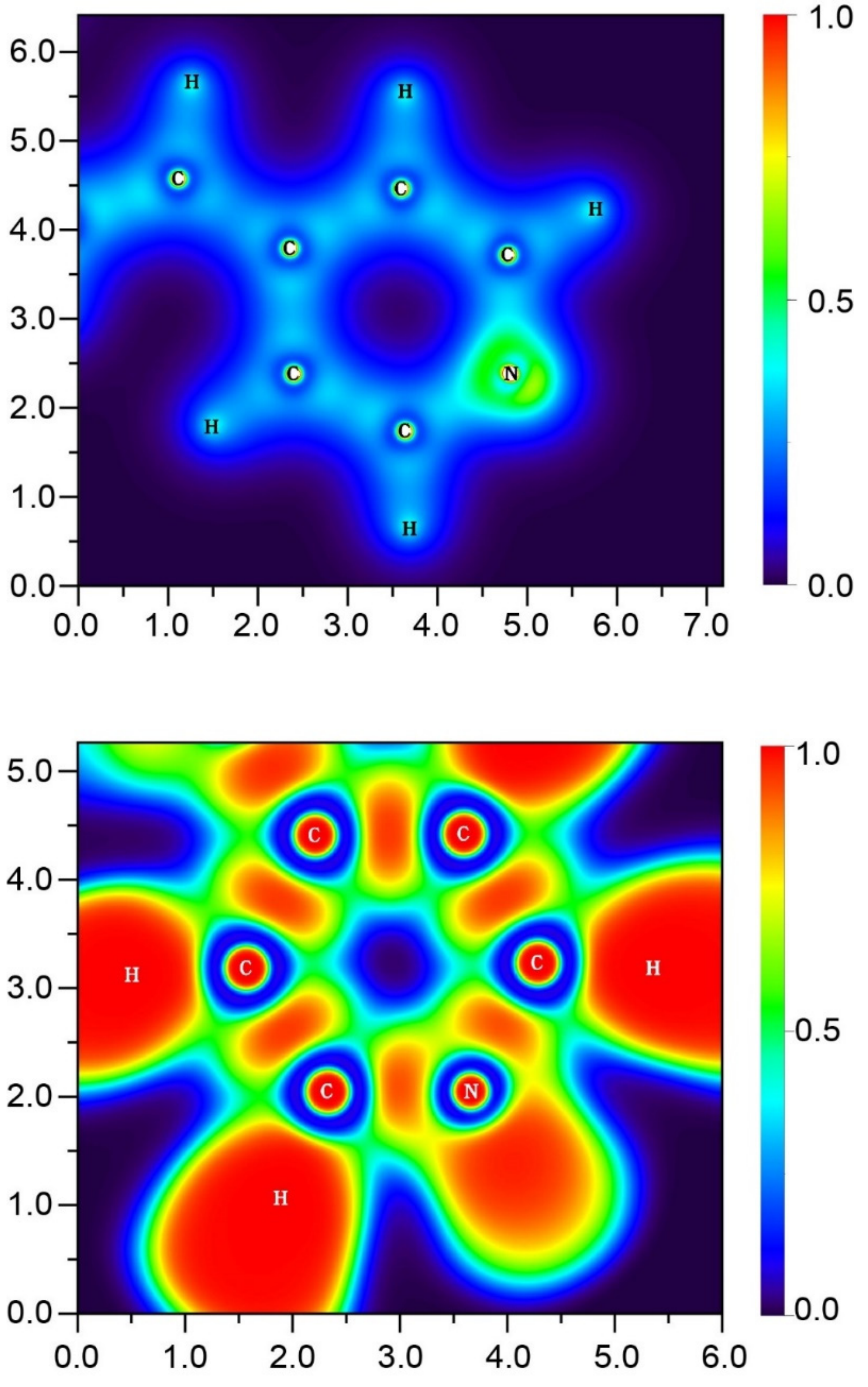

Supplementary Fig. 10. Color-filled maps of valence electron density and ELF of **BPE**

9. *Color-filled maps of valence electron density and ELF of* ***BDBB***

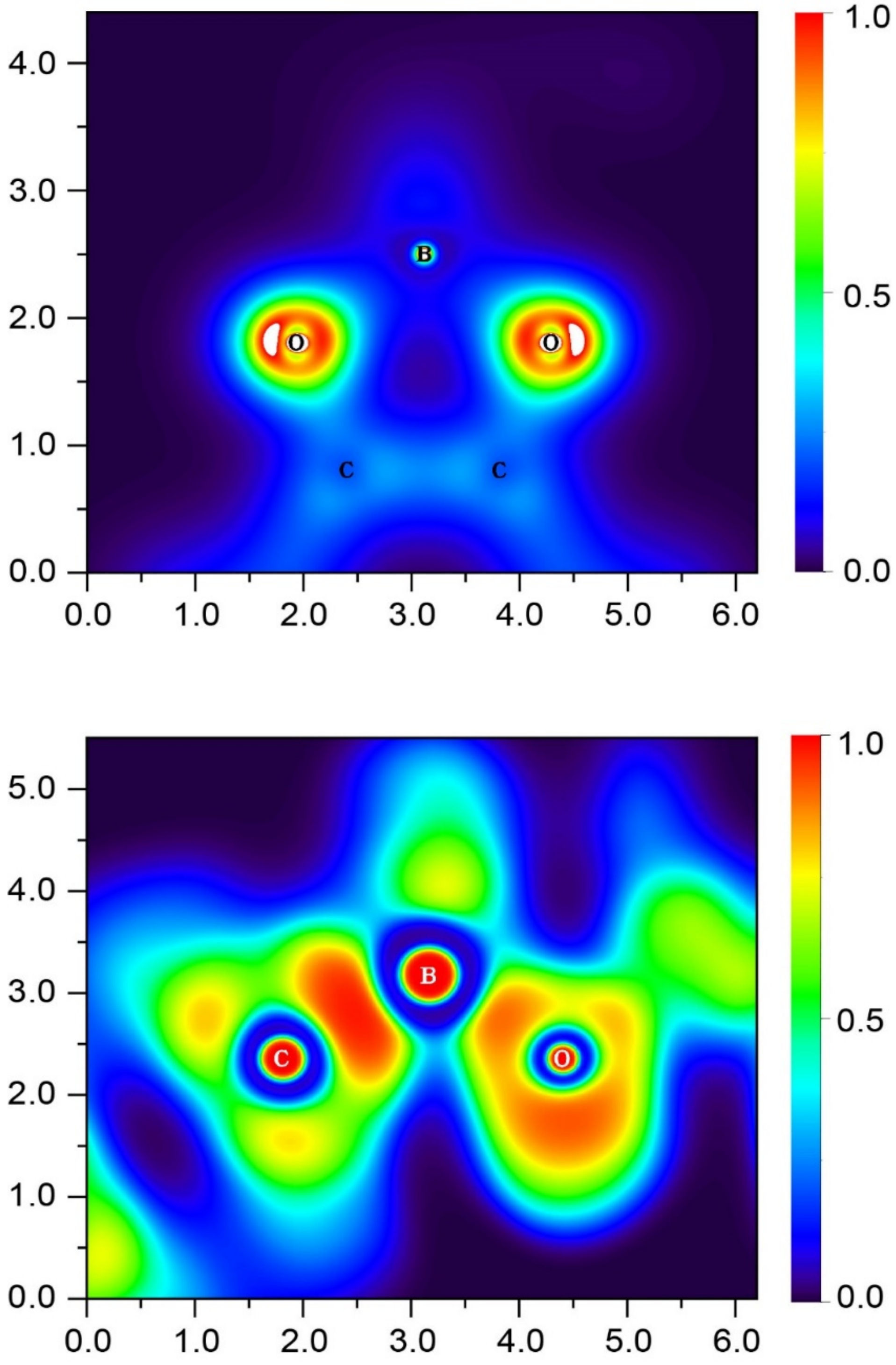

Supplementary Fig. 11. Color-filled maps of valence electron density and ELF of **BDBB**. The ELF value is low on the top of the B atom, which implies that the electron density is extremely low in this region.

10. *Color-filled maps of valence electron density and ELF of the dative compounds formed by* ***BDBB*** *and* ***BPE***

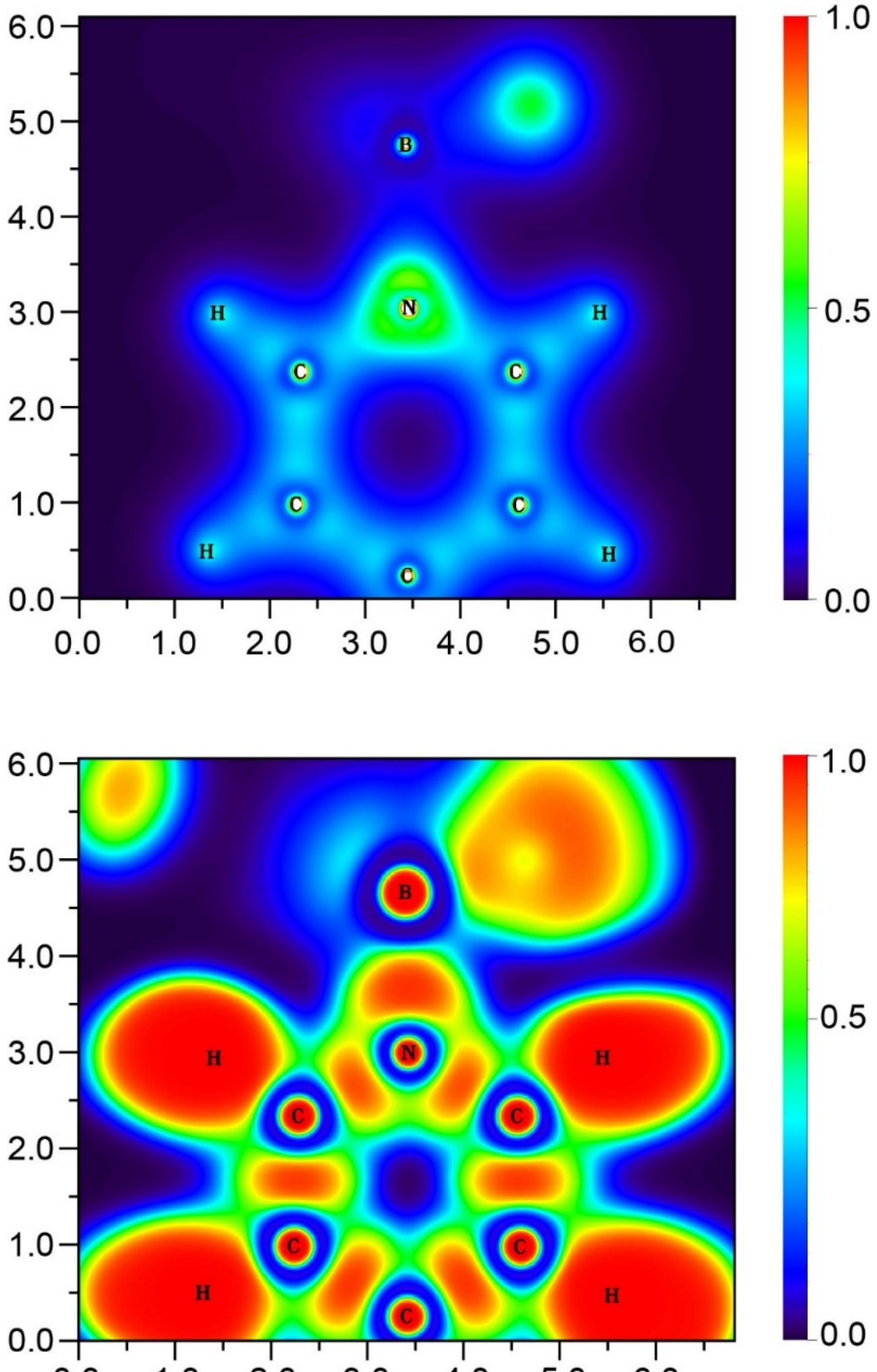

Supplementary Fig. 12. Color-filled maps of valence electron density and ELF of the complex of **BDBB** and **BPE**. There is a large ELF value between the B and N atoms, which means that a new chemical bond has been formed between the B and N atoms and that the bond energy of this new chemical bond is large.

11. *Crystal structure of **2DWPN-1***

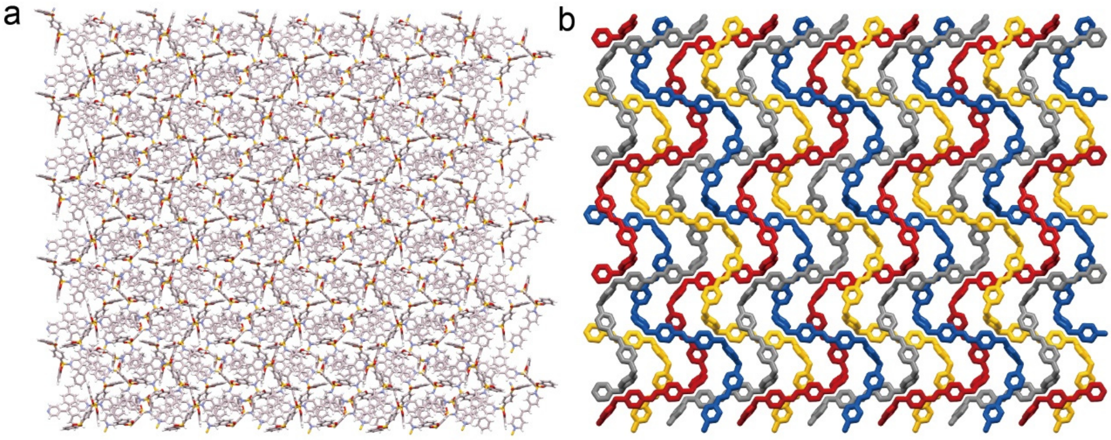

Supplementary Fig. 13. (**a**) Top view of a monolayer of the crystal packing in **2DWPN-1**. (**b**) Simplified top view of a monolayer of the crystal packing in **2DWPN-1**. The independent chains are distinguished by different colors. Solvent molecules, H atoms and catechol groups were removed for the sake of presenting a clearer woven display.

12. *Typical [π···π] interactions between polymer chains in the crystal structure of **2DWPN-1***

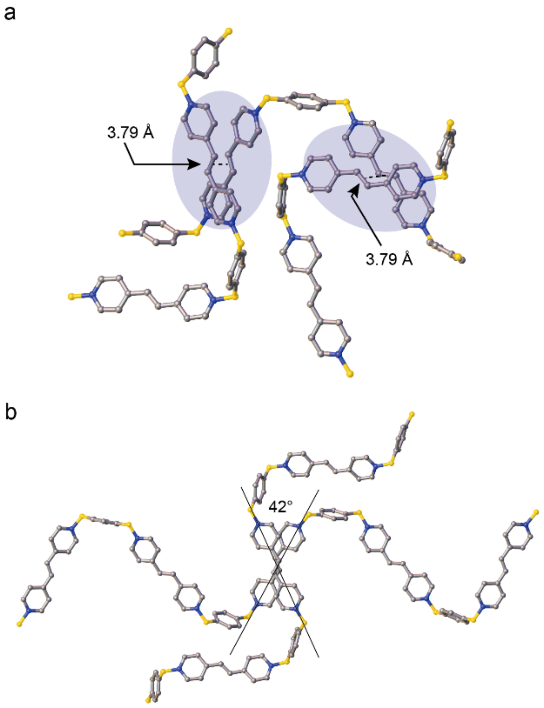

Supplementary Fig. 14. (**a**) Typical distance (3.79 Å) between two **BPE** moieties in the woven nodes of the crystal structure of **2DWPN-1**. (**b**) Two crossing molecular chains in the crystal

structure of **2DWPN-1**

13. *The effect of solvents on the crystallinity of crystal* ***2DWPN-1***

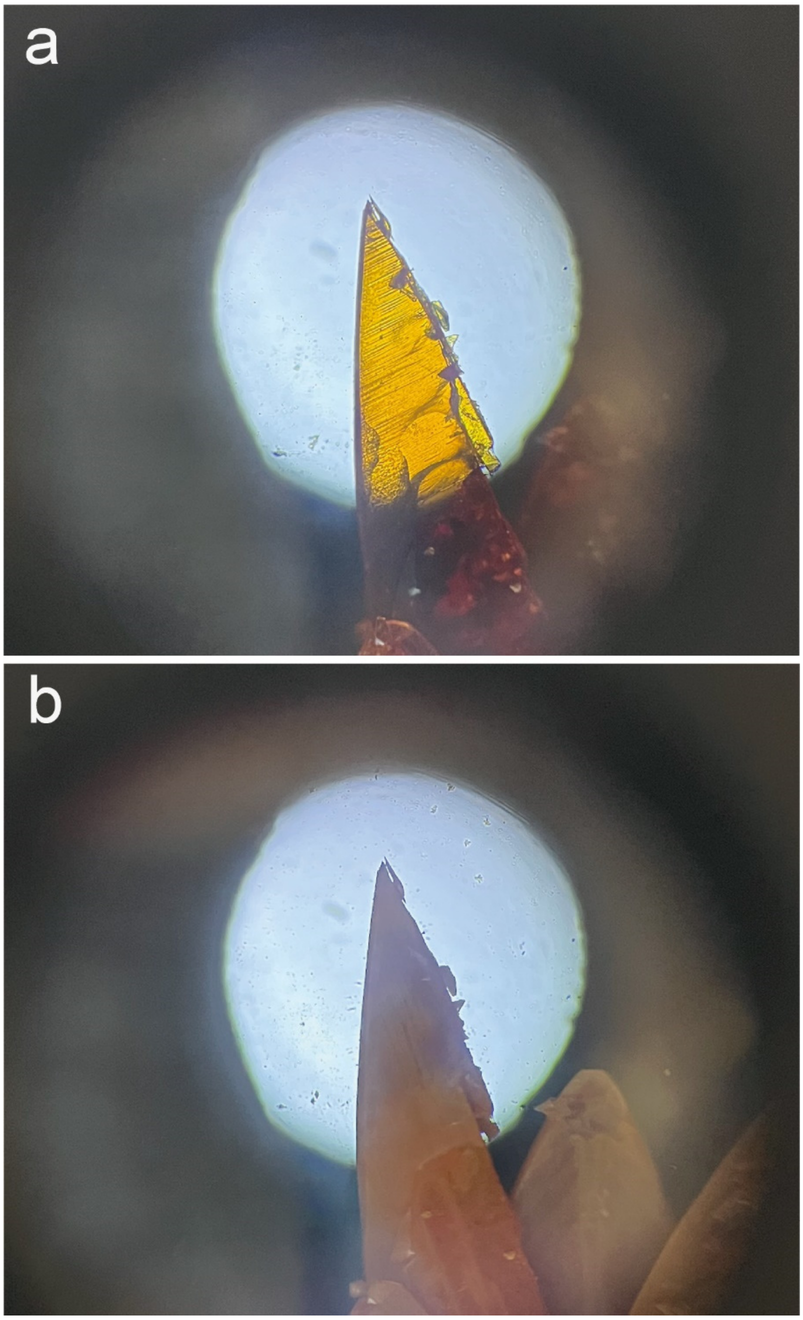

Supplementary Fig. 15. Optical microscope photographs depict the crystal of **2DWPN-1** before (**a**) and after (**b**) solvent removal. Upon heating to 120 °C, the solvent within the crystals gradually evaporates, resulting in the opacity and loss of crystallinity in the crystal of **2DWPN-1**.

14. *Characterization of **NWPN-1** crystals*

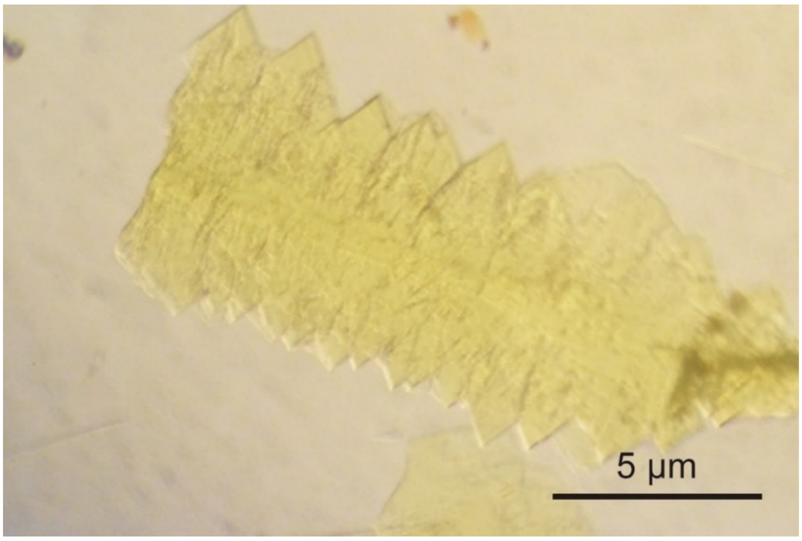

Supplementary Fig. 16. Optical microscopic (OM) image of **NWPN-1** crystals obtained as plates

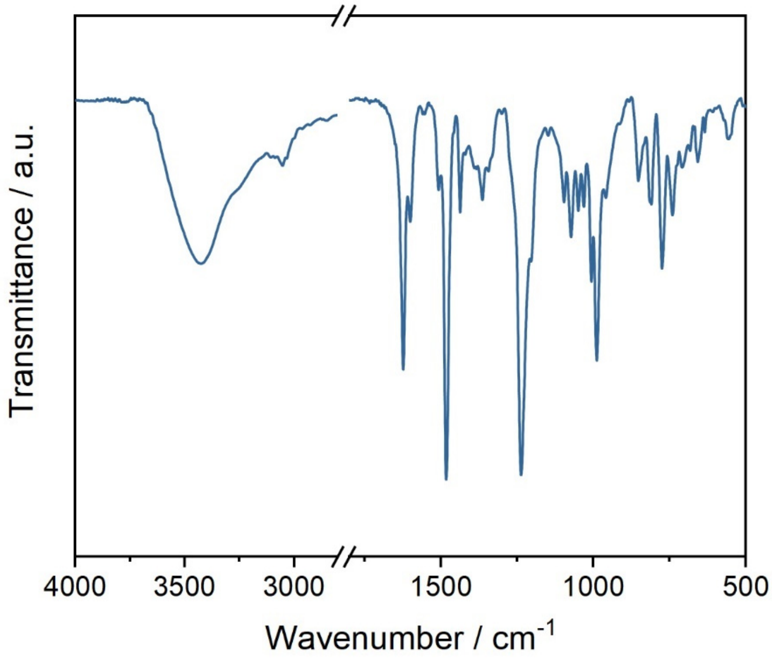

Supplementary Fig. 17. Infrared spectra of **NWPN-1**. Wavenumbers 1482 and 1238 cm^{-1} correspond to the characteristic absorption peaks of a B−O bond, and 1362 cm^{-1} corresponds to the characteristic absorption peak of a dative B−N bond

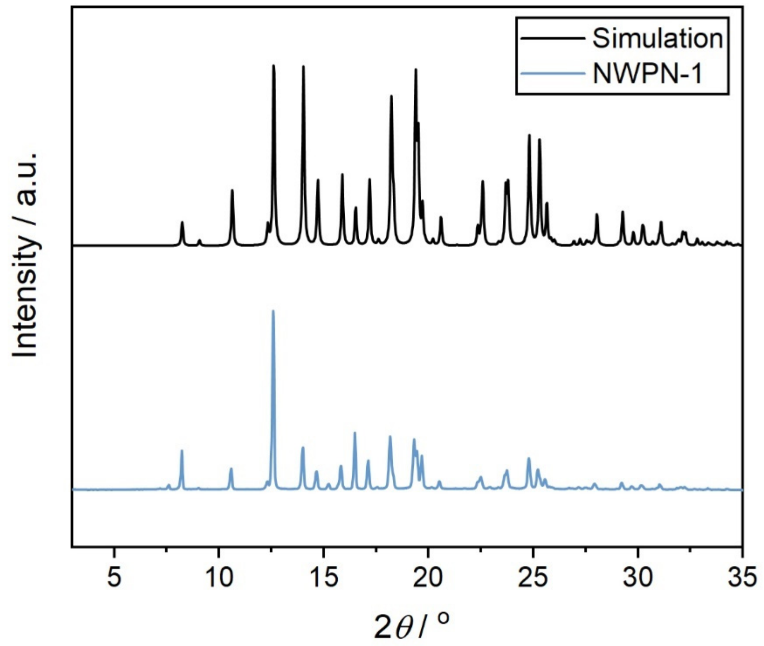

Supplementary Fig. 18. The PXRD patterns of the bulk crystals of **NWPN-1**. The experimental data match well with the PXRD pattern simulated from the single crystal diffraction data.

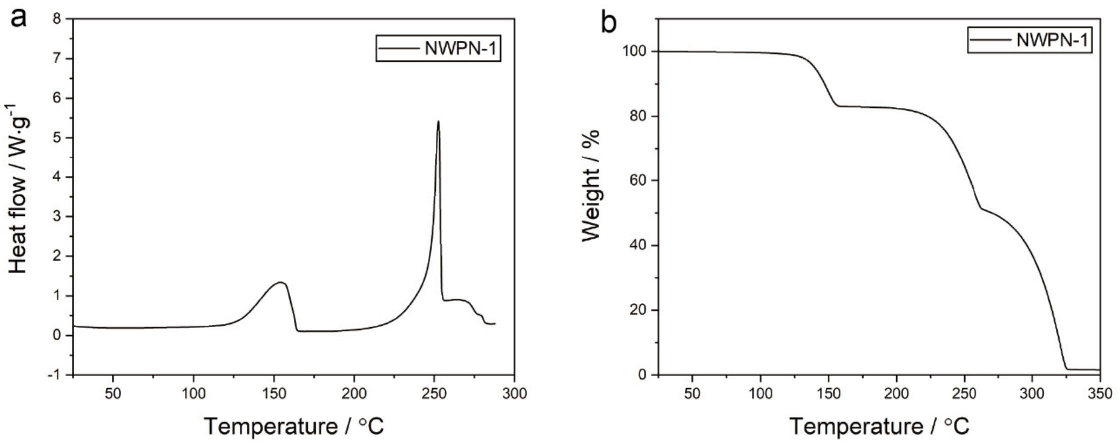

Supplementary Fig. 19. (**a**) DSC thermograms of **NWPN-1.** (**b**) TGA curve of **NWPN-1**

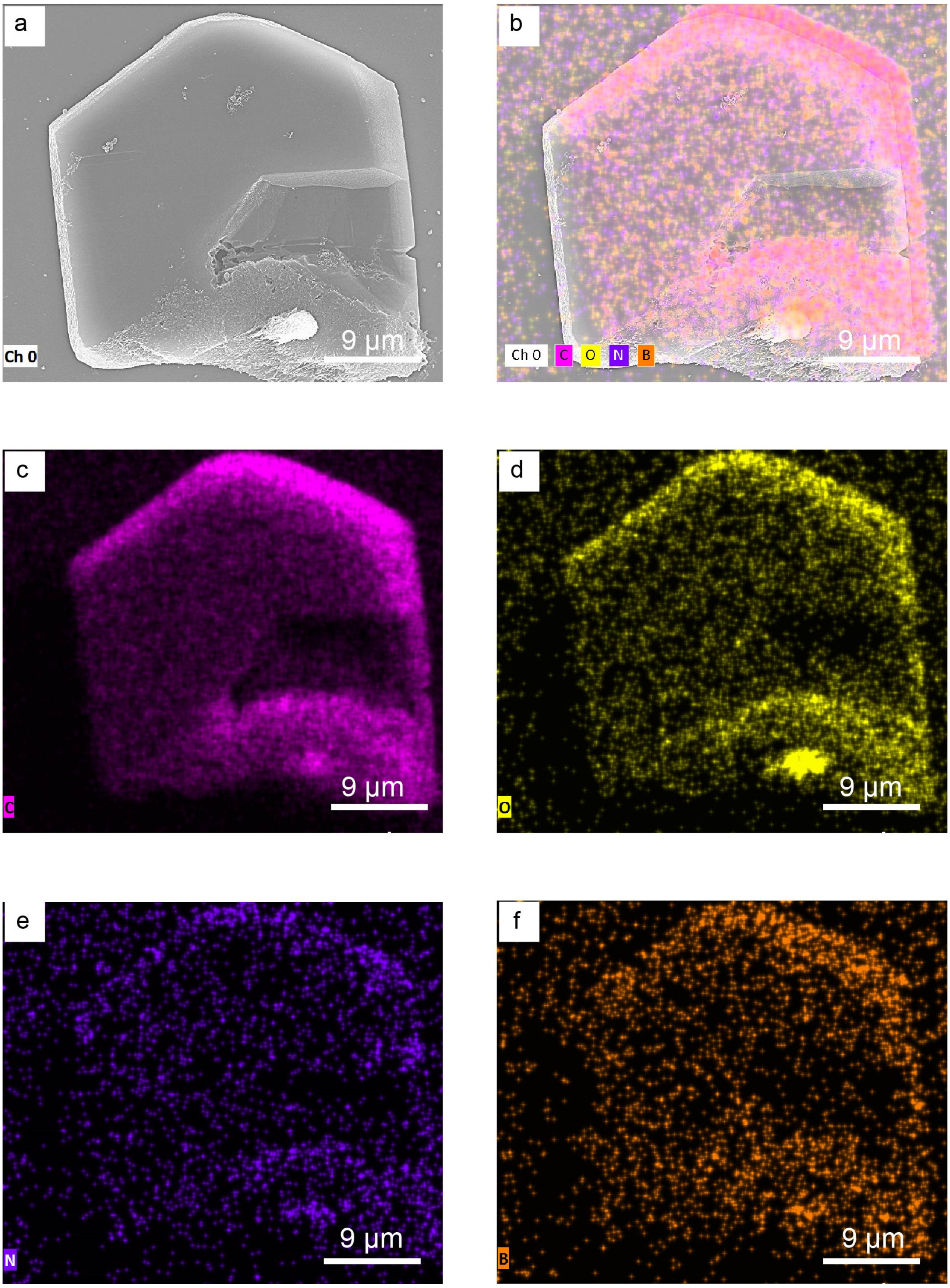

Supplementary Fig. 20. SEM Image (**a**) and the EDS mapping (**b**) of **NWPN-1**. The corresponding elemental maps: carbon (**c**), oxygen (**d**), nitrogen (**e**), and boron (**f**)

15. *Crystal structure of* ***NWPN-1***

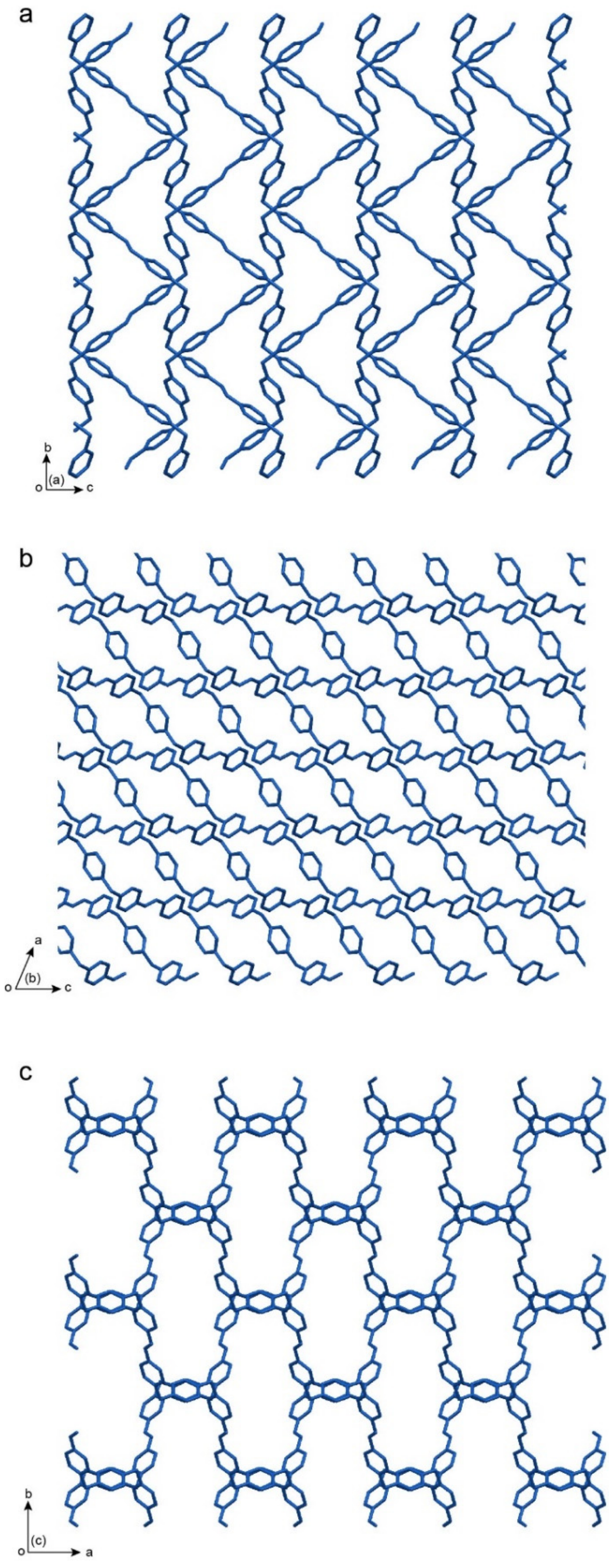

Supplementary Fig. 21. The crystal structure of **NWPN-1**. (**a**, **b**, and **c**) views along the *a*, *b*, and *c* axes. H atoms, catechol groups and *meta*-xylene are hidden to present a clear display.

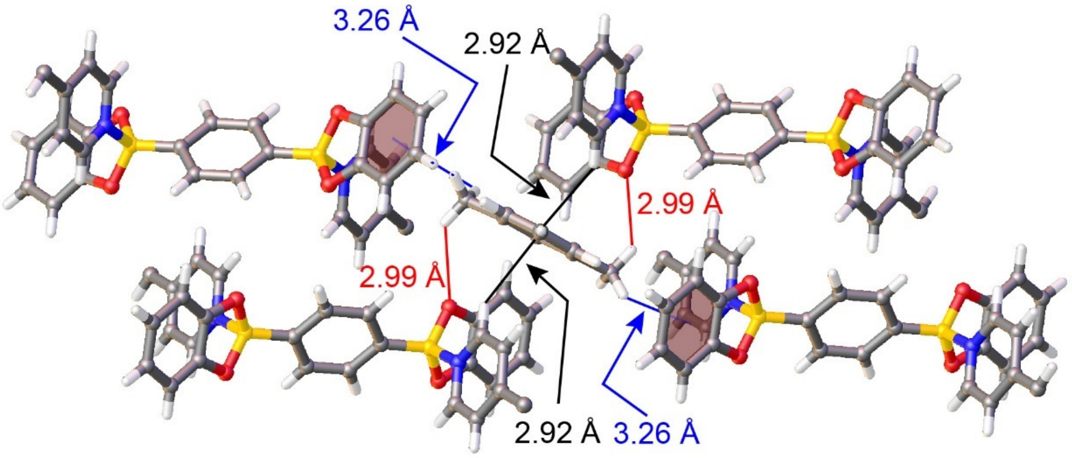

Supplementary Fig. 22. Illustration of interactions between a *meta*-xylene molecule and the surrounding four polymer chains in the crystal structure of **NWPN-1**. The *meta*-xylene solvent molecule is connected to the surrounding four polymer chains by [C−H···*π*] (2.92 Å−3.26 Å) and [C−H···O] (2.99 Å) interactions.

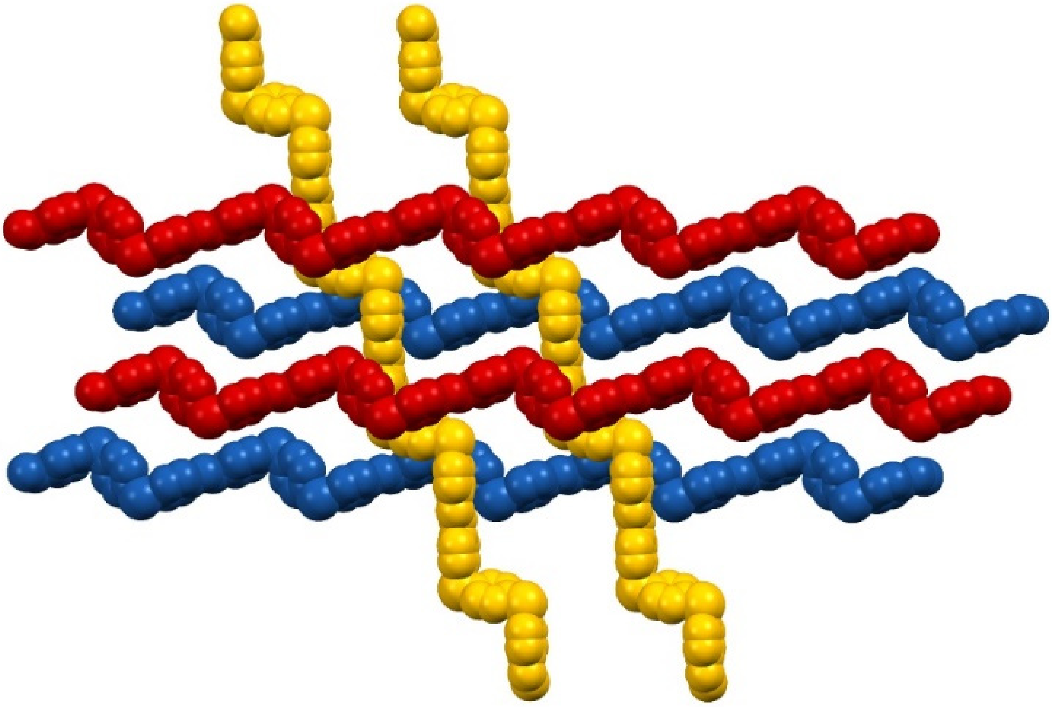

Supplementary Fig. 23. Entanglement of polymer chains in the crystal structure of **NWPN-1**. Parallel stacking of polymer chains in two directions.

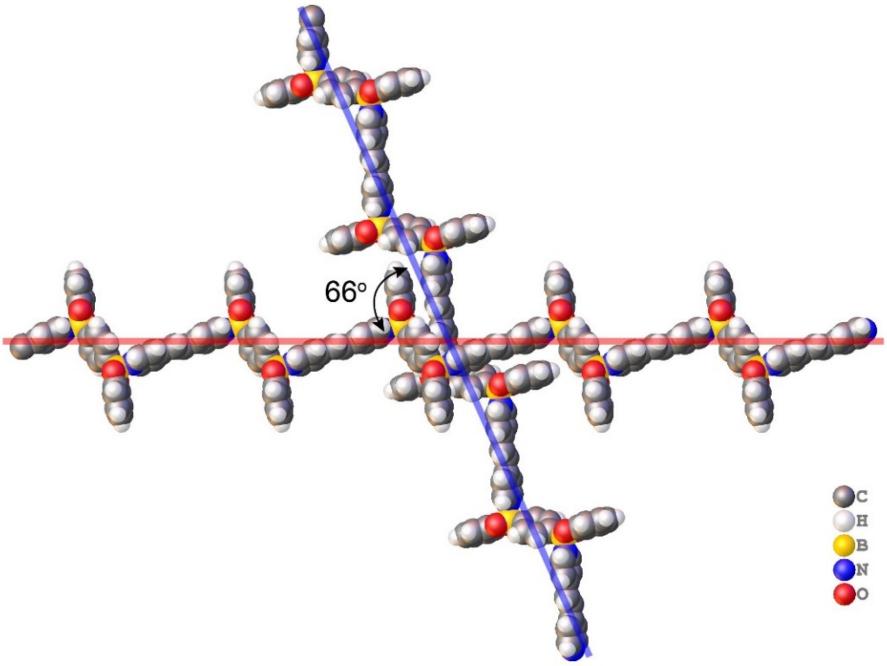

Supplementary Fig. 24. The angle between polymer chains is 66° in **NWPN-1** crystals.

16. *Crystal structure of* ***2DWPN-1***

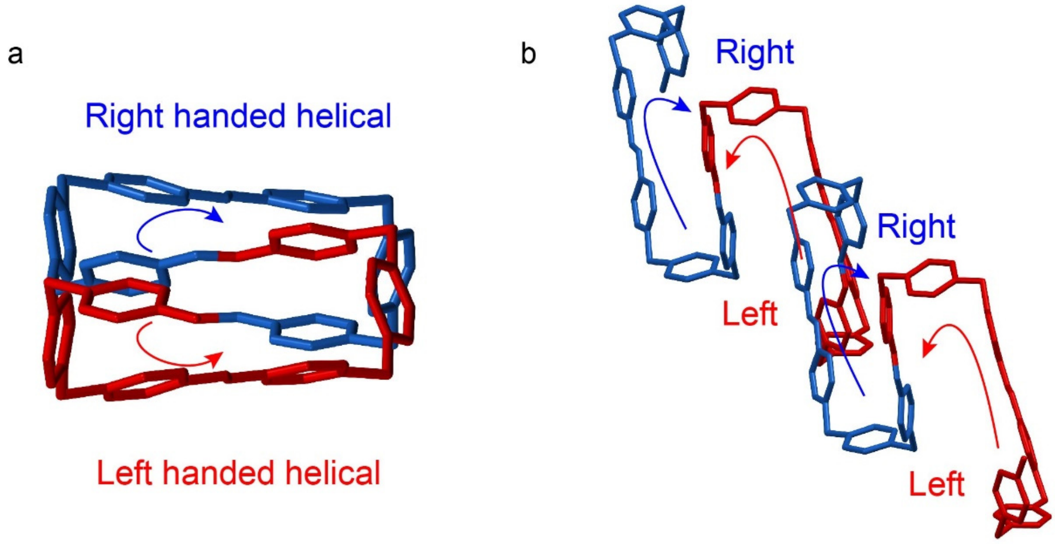

Supplementary Fig. 25. Polymer chain conformations of **2DWPN-1**. (**a**) The view along the extended direction of a polymer chain. (**b**) The side-on view of the polymer chain of **2DWPN-1**. The polymer chain is formed by the arrangement of two spirals in opposite directions, and the two spirals are alternately arranged. This polymer chain conformation forms two alternate spaces.

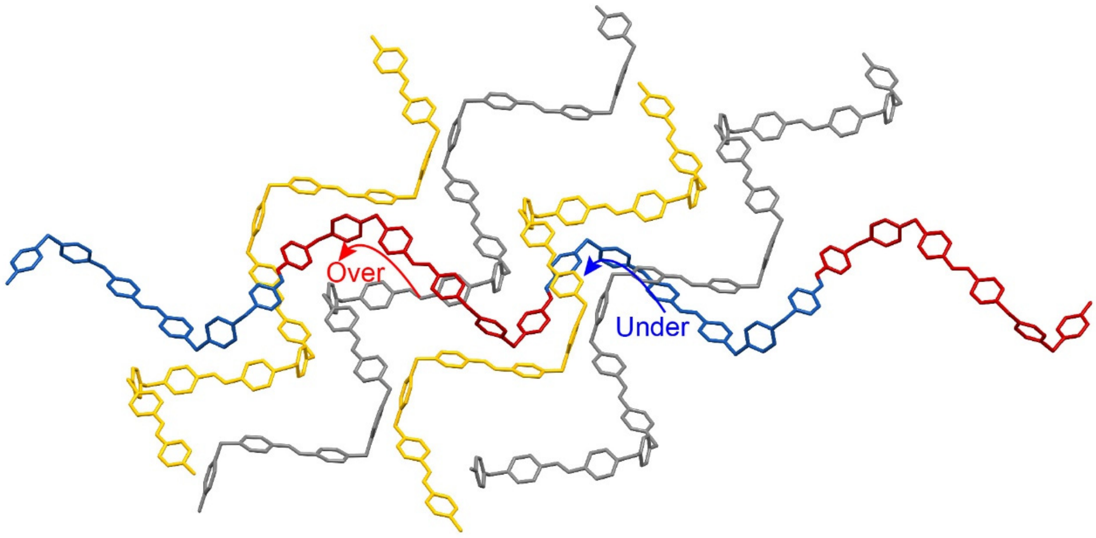

Supplementary Fig. 26. The intercalation of polymer chains in the crystal structure of **2DWPN-1**. The spaces are formed by two reverse helices in a polymer chain, causing the polymer chains to cross in a way that there are two over and two under. This kind of crossover finally forms a typical biaxial weaving.

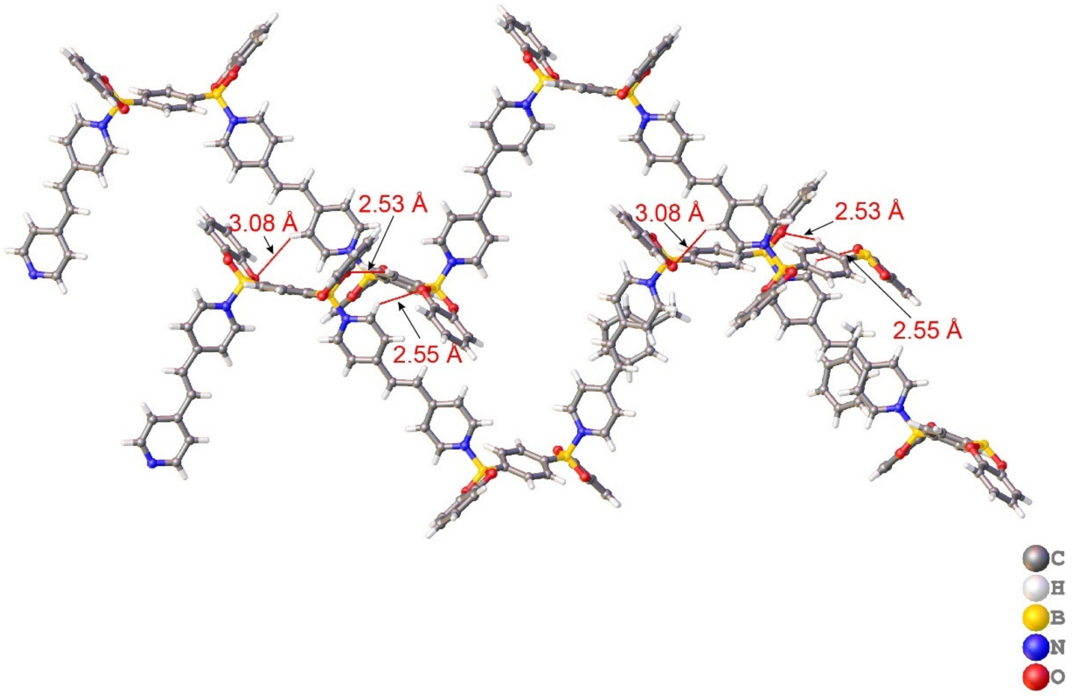

Supplementary Fig. 27. The interaction between parallel chains in the structure of **2DWPN-1**. Two parallel chains are linked by [C−H···O] (3.08 Å, 2.55 Å and 2.53 Å) interactions.

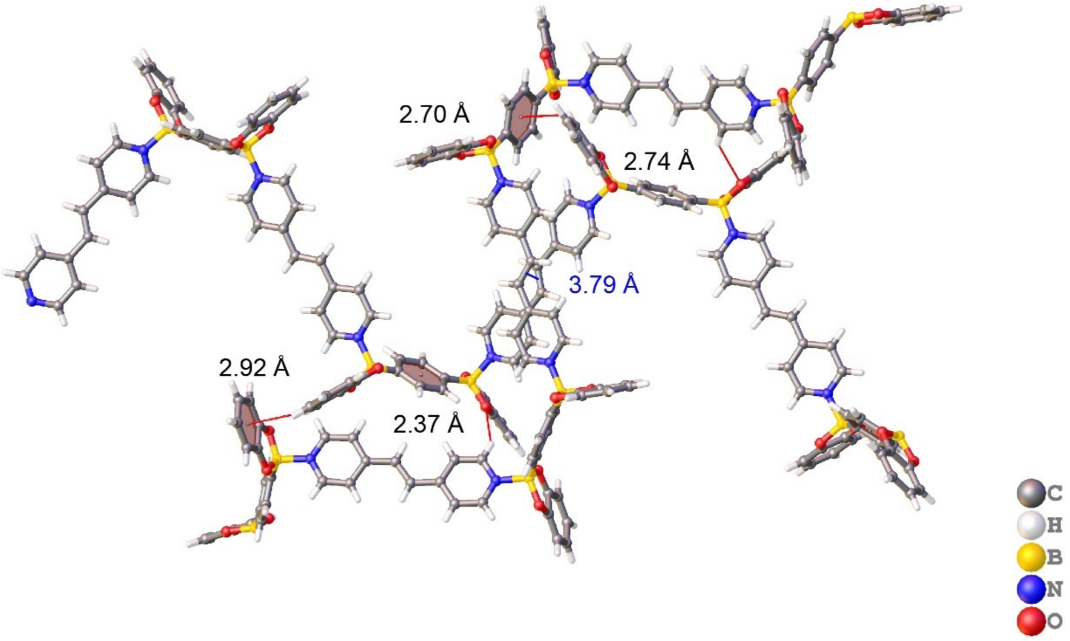

Supplementary Fig. 28. Interactions between woven chains in the crystal structure of **2DWPN-1**. Two chains are woven as a result of [$\pi\cdots\pi$] (3.79 Å) interactions.

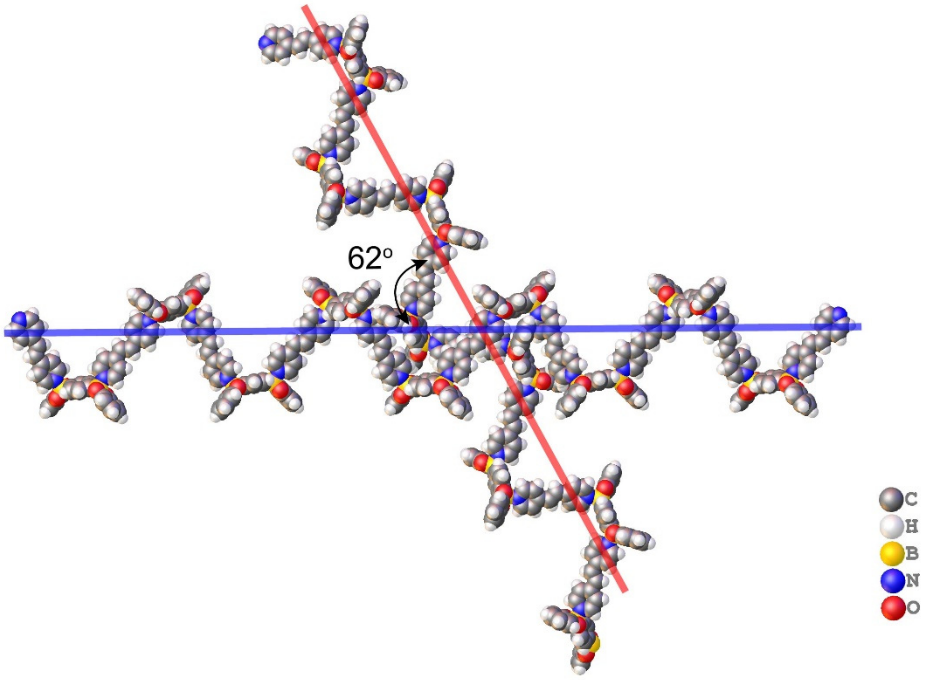

Supplementary Fig. 29. The angle between polymer chains is 62° in the crystal structure of **2DWPN-1**.

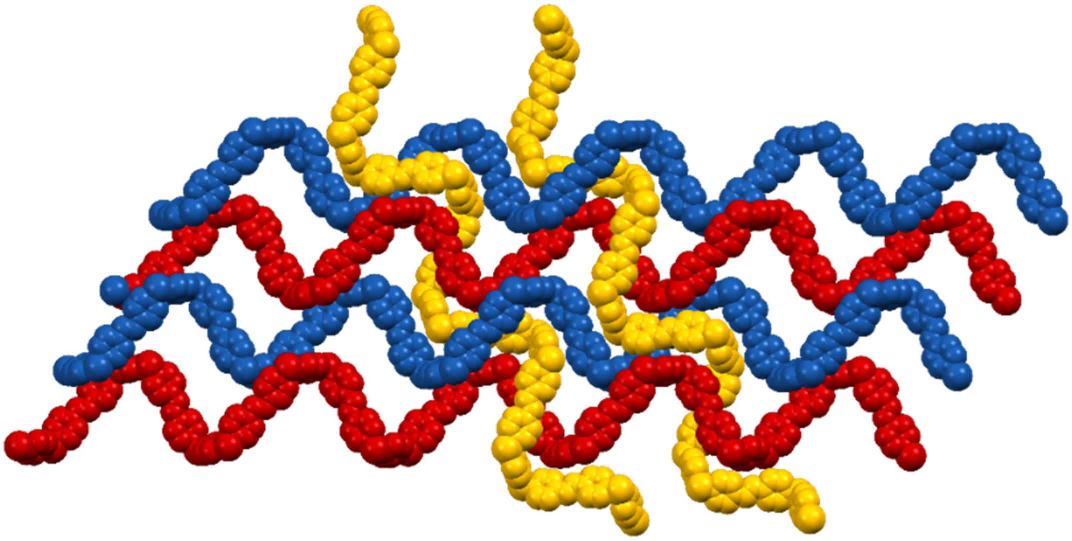

Supplementary Fig. 30. Entanglement of polymer chains in the crystal structure of **2DWPN-1**. The polymer chains in two directions form a 2D woven network by entanglement.

17. *Characterization of **2DWPN-2** crystals*

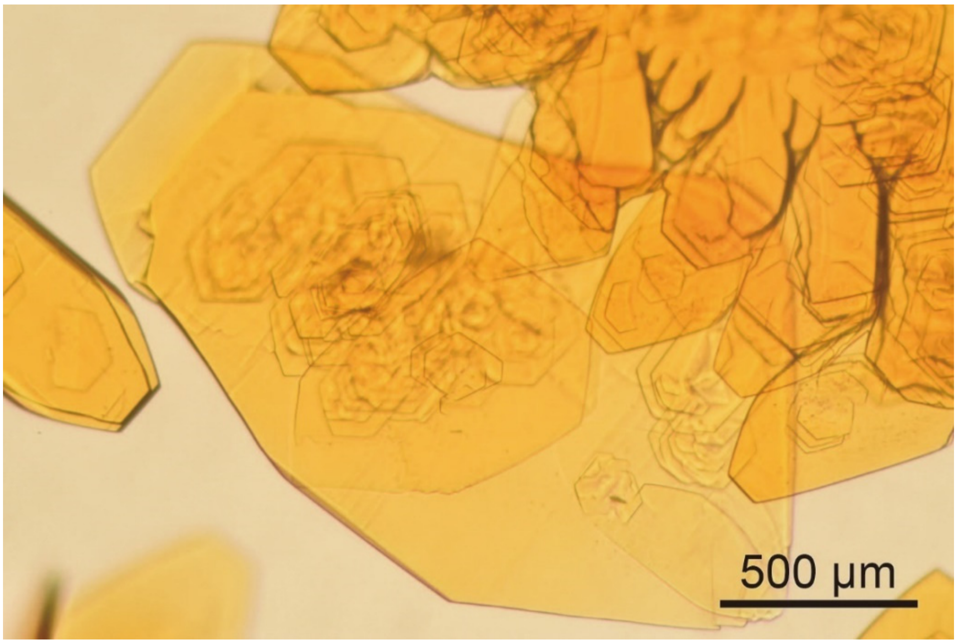

Supplementary Fig. 31. OM Image of **2DWPN-2** crystals obtained as plates

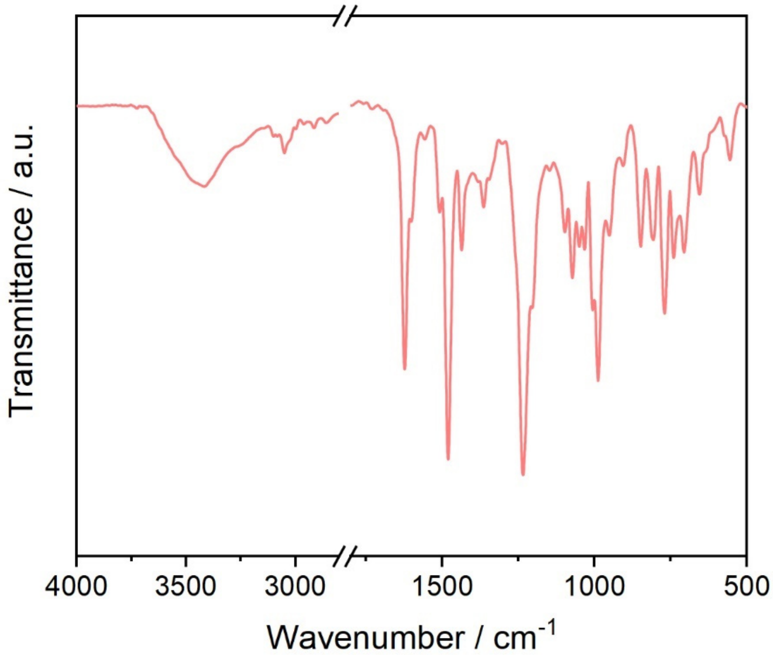

Supplementary Fig. 32. Infrared spectra of **2DWPN-2**. Wavenumbers 1481 cm^{-1} and 1234 cm^{-1} correspond to the characteristic absorption peaks of the B−O bond and 1362 cm^{-1} corresponds to the characteristic absorption peak of the dative B−N bond.

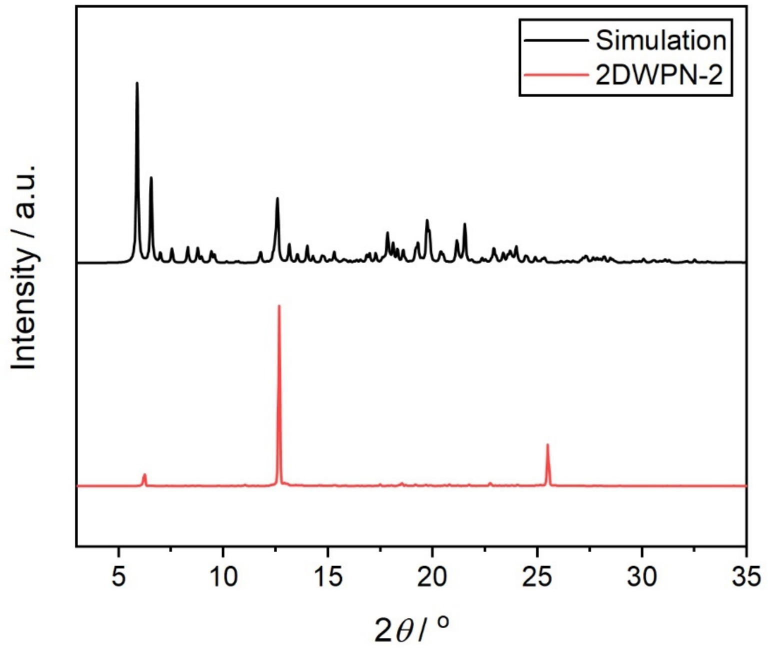

Supplementary Fig. 33. The PXRD patterns of the bulk crystals of **2DWPN-2**

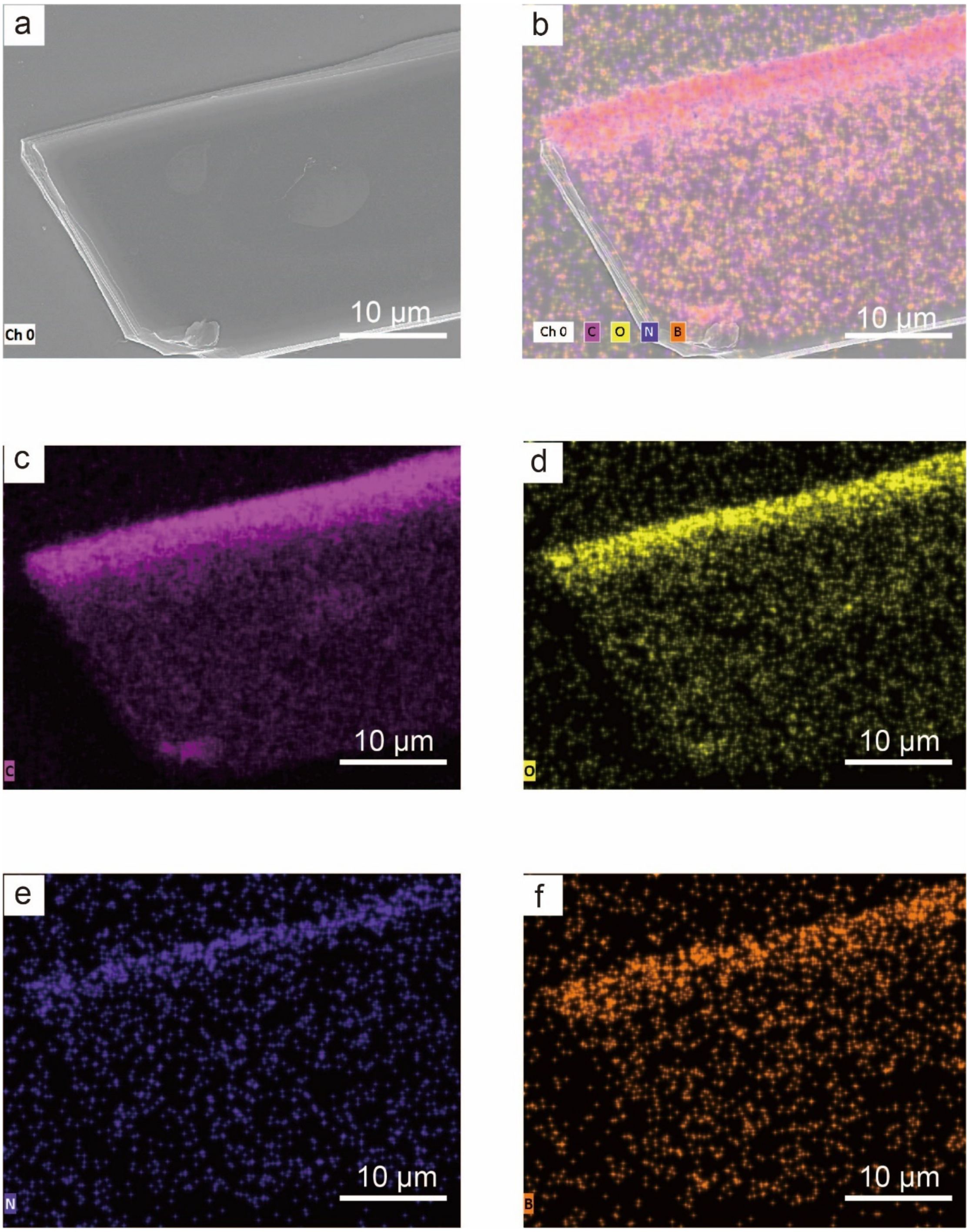

Supplementary Fig. 34. SEM Image (**a**) and the EDS mapping (**b**) of **2DWPN-2**. The corresponding elemental maps: carbon (**c**), oxygen (**d**), nitrogen (**e**), and boron (**f**)

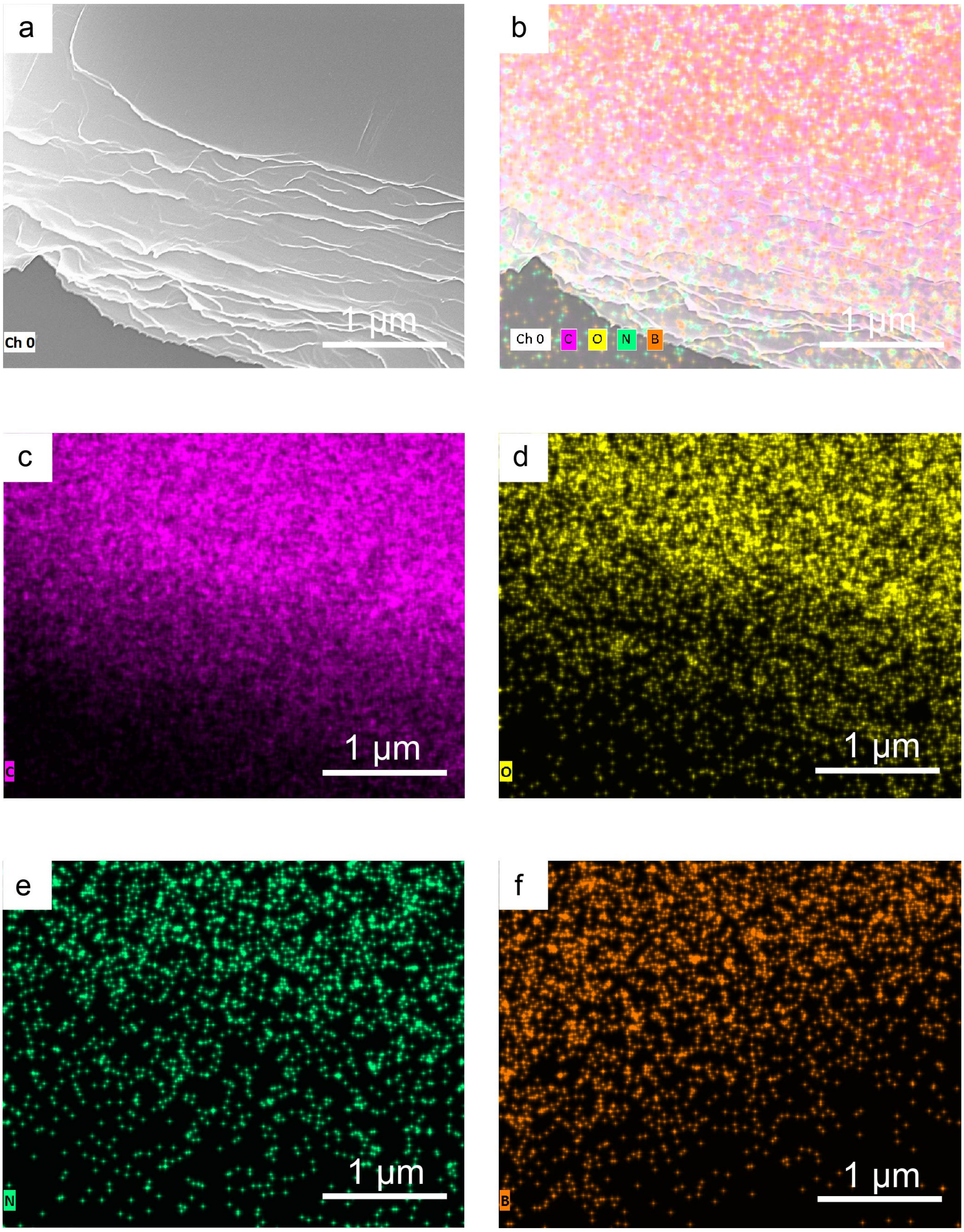

Supplementary Fig. 35. SEM Image (**a**) and the EDS mapping (**b**) of the cross section of **2DWPN-2**. The corresponding elemental maps: carbon (**c**), oxygen (**d**), nitrogen (**e**), and boron (**f**)

18. *Crystal structure of **2DWPN-2***

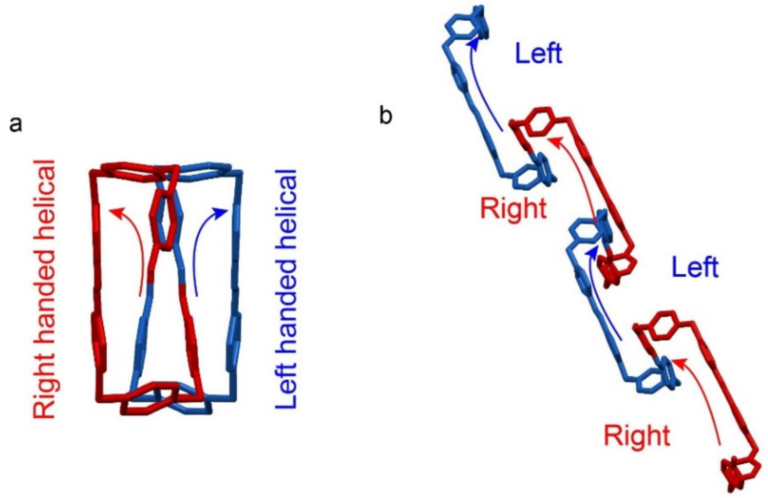

Supplementary Fig. 36. Polymer chain conformation of **2DWPN-2**. (**a**) The view along the extension of a polymer chain. (**b**) The side view of the polymer chain in **2DWPN-2**. The repeated extension of the twisted fragments results in the helical polymer chain conformation. This polymer chain conformation forms two alternate spaces.

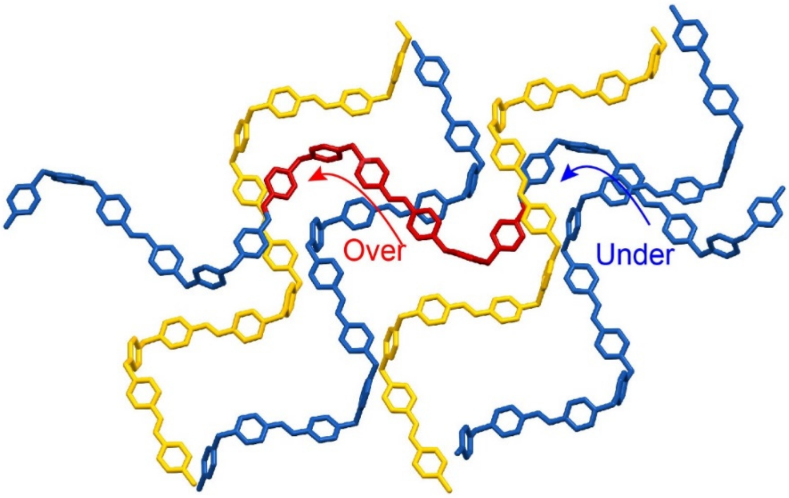

Supplementary Fig. 37. The intercalation of polymer chains in the crystal structure of **2DWPN-2**. The spaces are formed by two reverse helices in a polymer chain, causing the polymer chain to cross in a way of two over and two under. This kind of crossover finally forms a typical biaxial weaving.

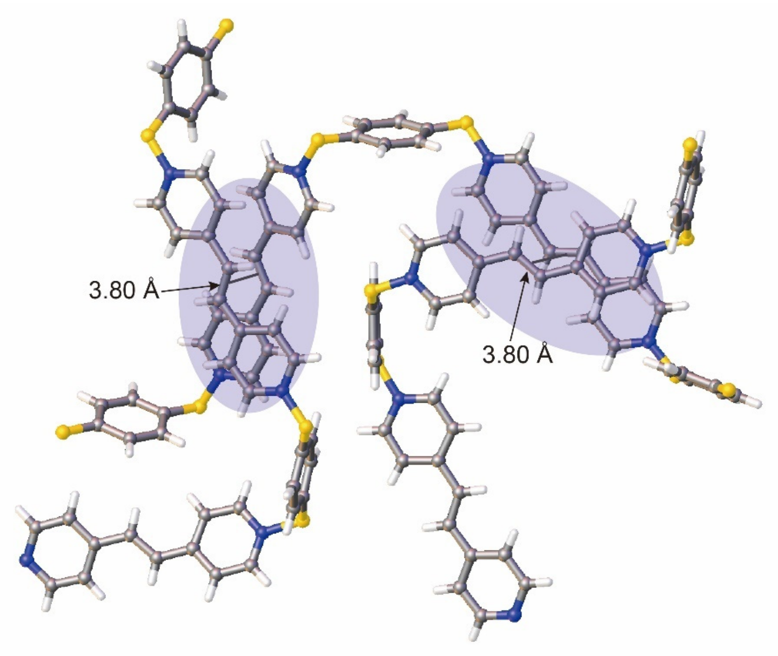

Supplementary Fig. 38. Typical [$\pi \cdots \pi$] interactions (3.80 Å) between two **BPE** moieties in the woven nodes of **2DWPN-2** crystals

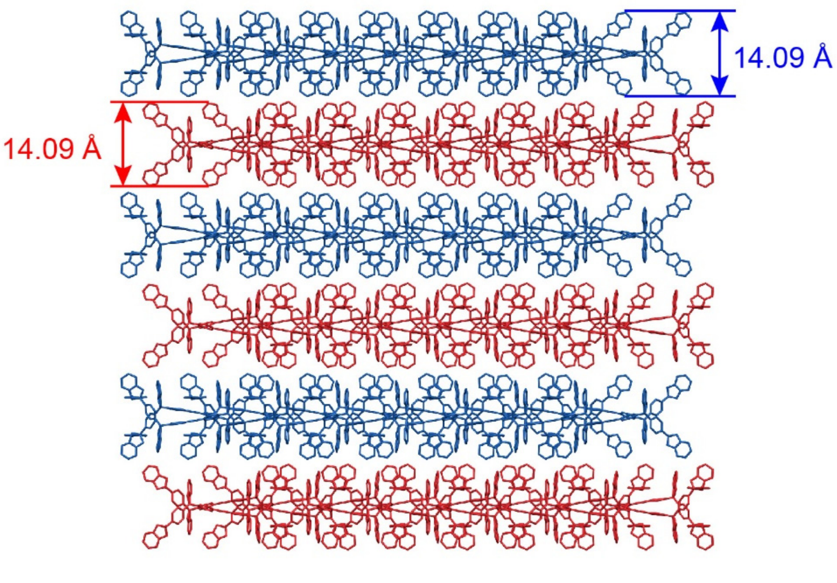

Supplementary Fig. 39. A view of the crystal structure of **2DWPN-2** along *a* axis. H atoms and *para*-xylene molecules were removed for the sake of clarity. The 2D woven monolayers adopt an anti-direction arrangement mode between the adjacent layers, forming a parallel and periodic three-dimensional (3D) multi-layer structure. The thickness of one layer of **2DWPN-2** is 14.09 Å.

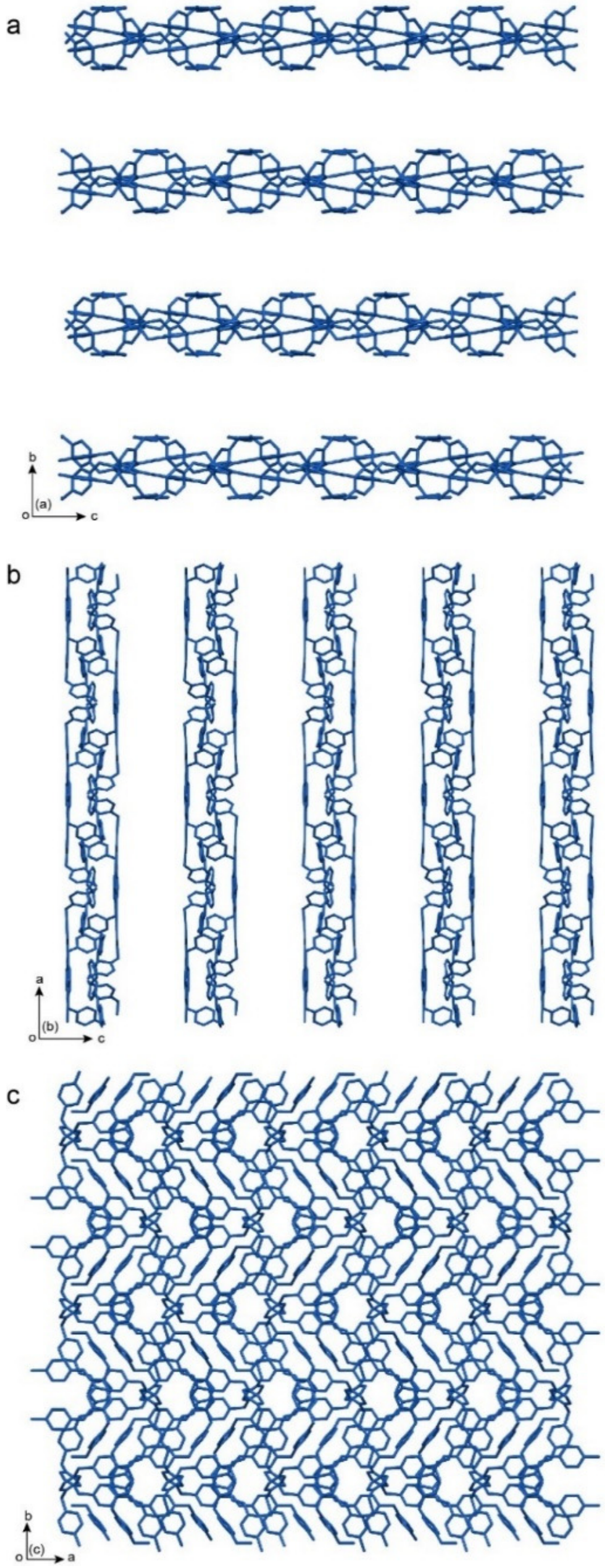

Supplementary Fig. 40. Crystal structure of **2DWPN-2**. (**a**, **b**, and **c**) views along the *a*, *b*, and *c* axes. H atoms, catechol groups and *para*-xylene molecules were hidden for the sake of clarity.

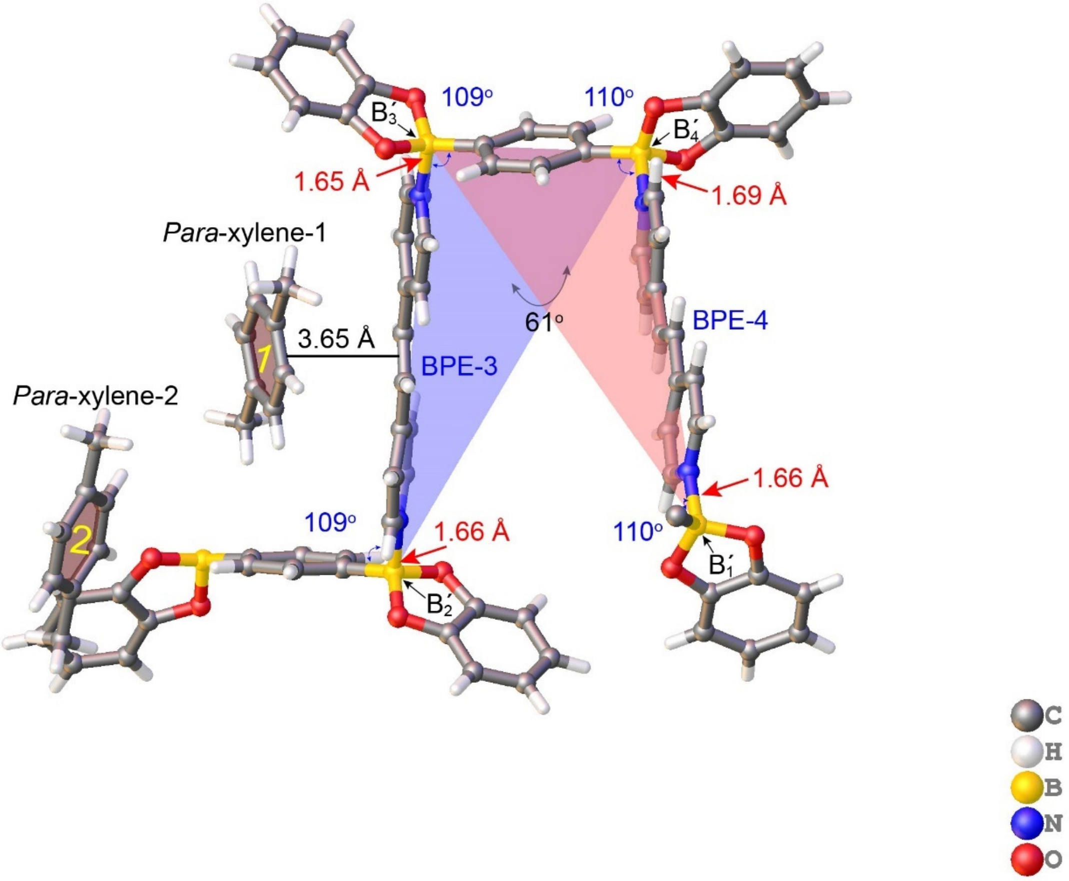

Supplementary Fig. 41. The interactions between the solvent molecules and polymer chains in an asymmetric unit of **2DWPN-2**. The ***para*-xylene-1** molecule forms [$\pi\cdots\pi$] interaction (3.65 Å) with **BPE-3** in the asymmetric unit. As a result, the dative B−N bonds of **BPE-3** with bond lengths of 1.66 Å and 1.65 Å are enhanced by the higher electron density. However, the interaction between ***para*-xylene-2** and **BPE-4** is weaker, and correspondingly, the dative B−N bonds possesses longer bond lengths of 1.69 Å and 1.66 Å. This asymmetric interaction results in a dihedral angle of 61° between the $B_2'B_3'B_4'$ and $B_1'B_4'B_3'$ planes related to two **BPE** units and one **BDBB** moiety during the polymerization.

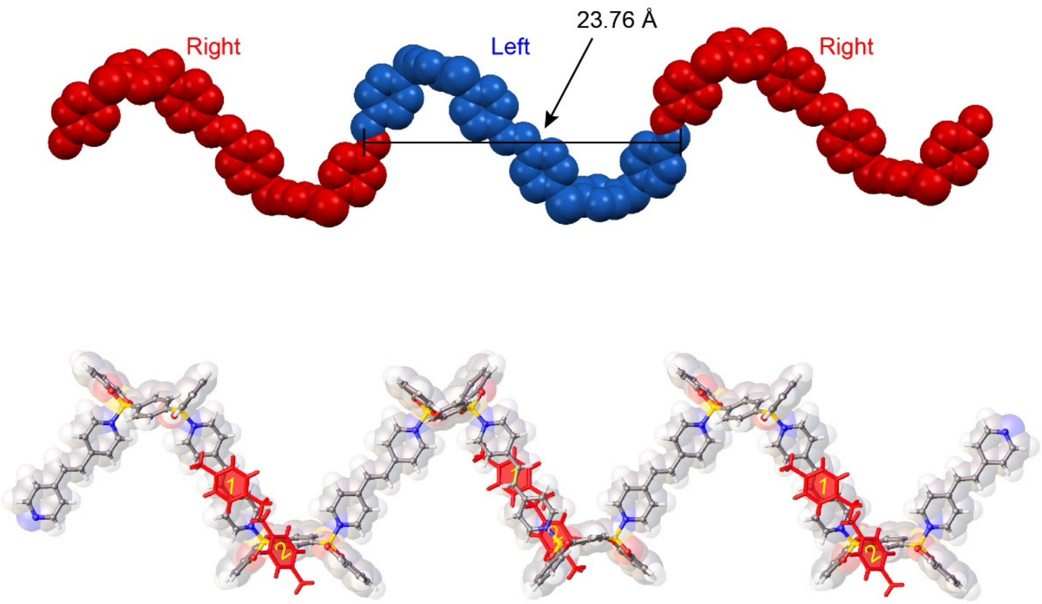

Supplementary Fig. 42. The polymer chain with helical conformation in **2DWPN-2**. The helical chain is composed of two helical segments with the same helical pitch of 23.76 Å, arranged alternately in opposite helical directions. The adjacent reverse spirals make up two alternating molecular bays with up and down openings.

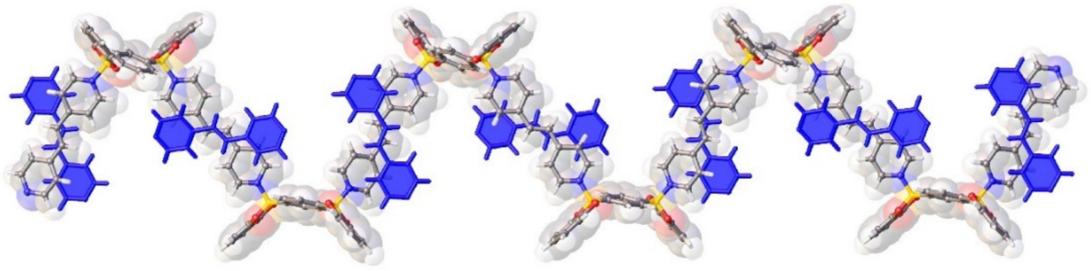

Supplementary Fig. 43. The entanglement of polymer chains at woven nodes in **2DWPN-2**. Each molecular bay contains two **BPE** moieties, thus, allowing two polymer chains to travel by an over or under space to form a typical biaxial woven topology.

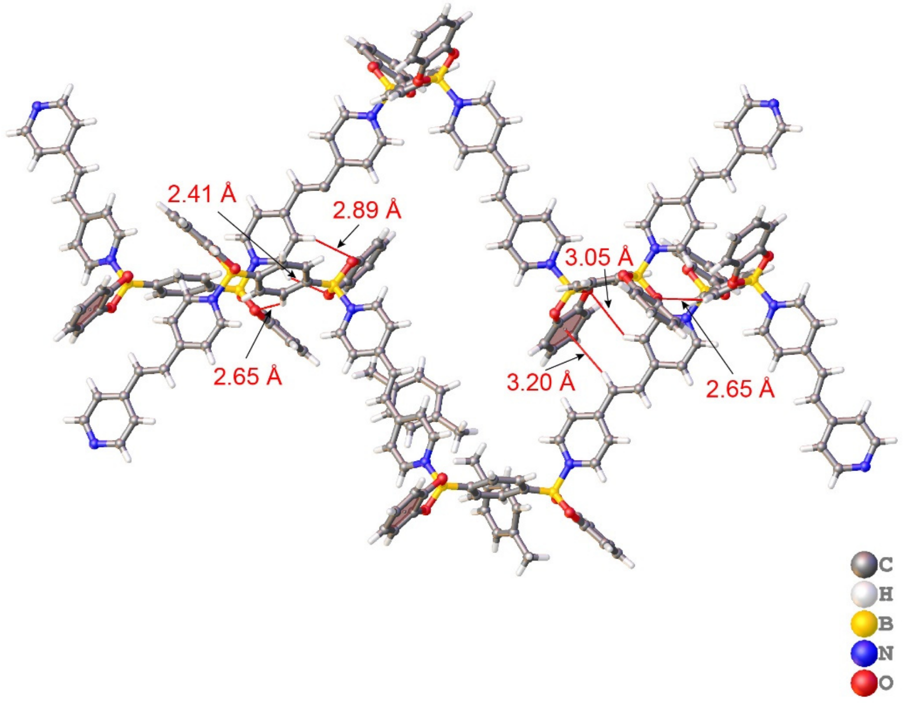

Supplementary Fig. 44. Interactions between two parallel chains in **2DWPN-2**. Two parallel chains are linked by [C−H···O] (2.65 Å, 2.41 Å and 3.05 Å) and [C−H···π] (2.89 Å, 3.20 Å) interactions.

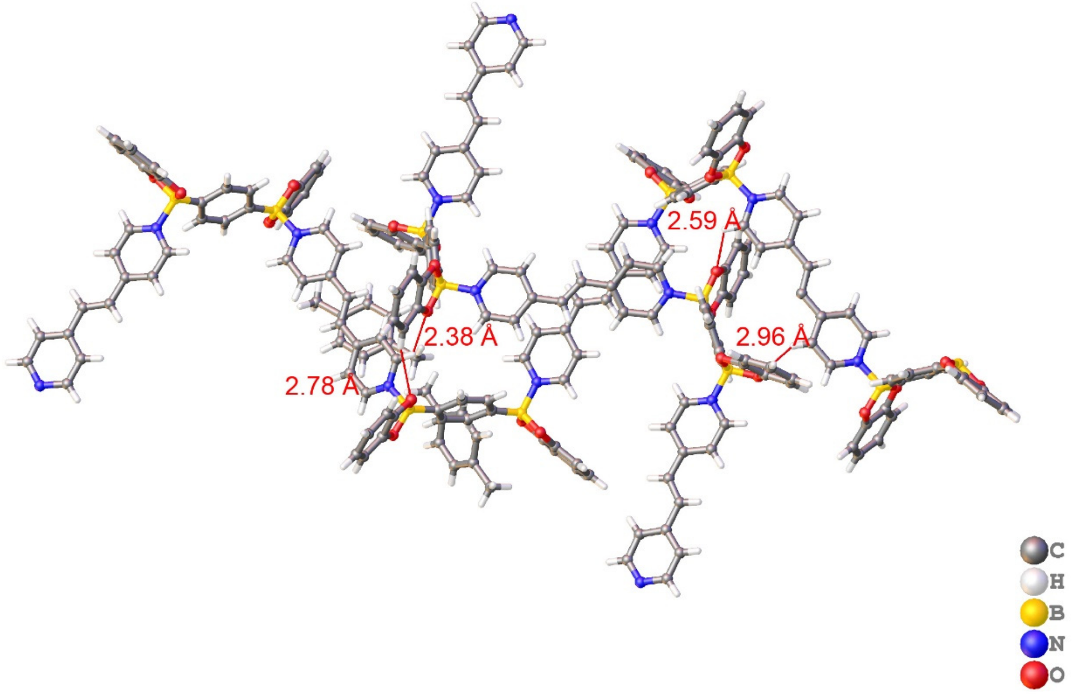

Supplementary Fig. 45. Interactions between two woven chains in **2DWPN-2**. Two woven chains are linked by [C−H···O] (2.78 Å, 2.38 Å, 2.59 Å and 2.96 Å) interactions.

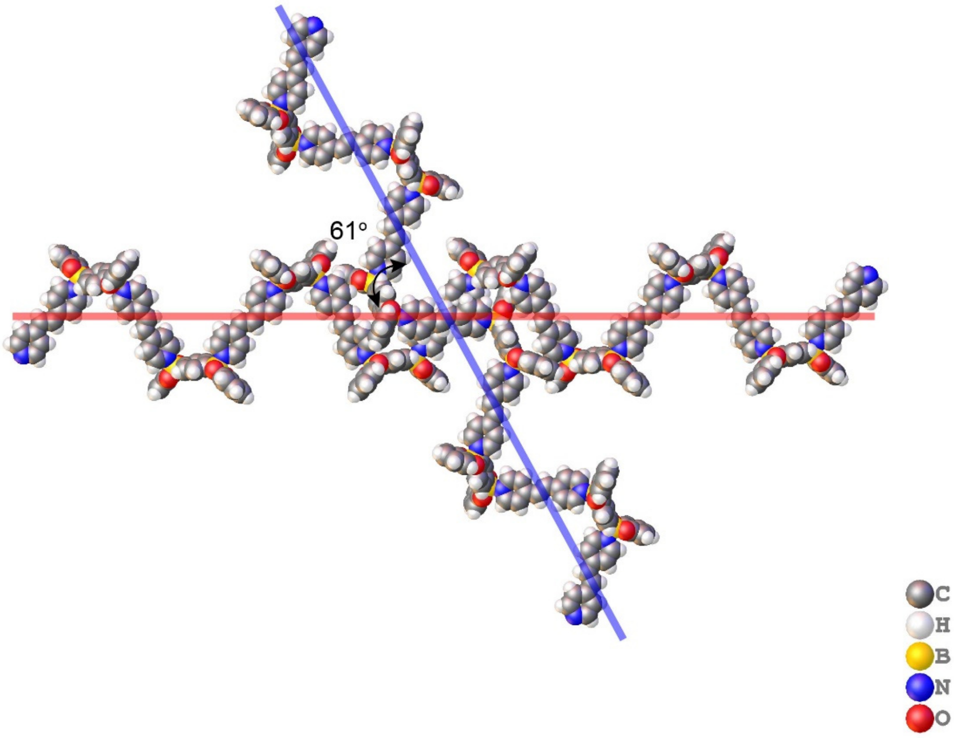

Supplementary Fig. 46. The angle between polymer chains is 61° in the crystal structure of **2DWPN-2**.

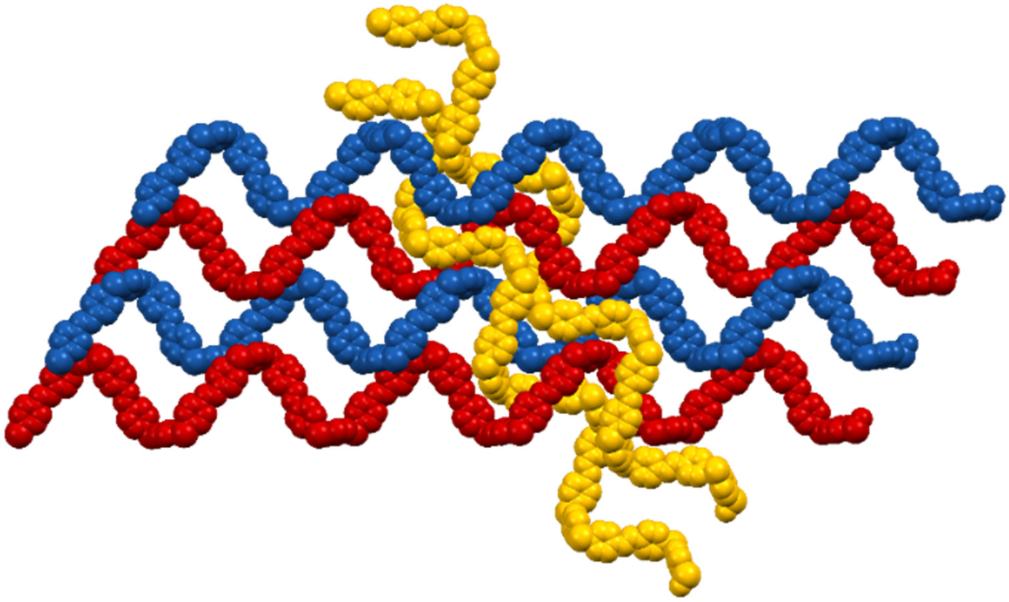

Supplementary Fig. 47. The entanglement of polymer chains in the crystal structure of **2DWPN-2**. The polymer chains in two directions form a woven network by entanglement.

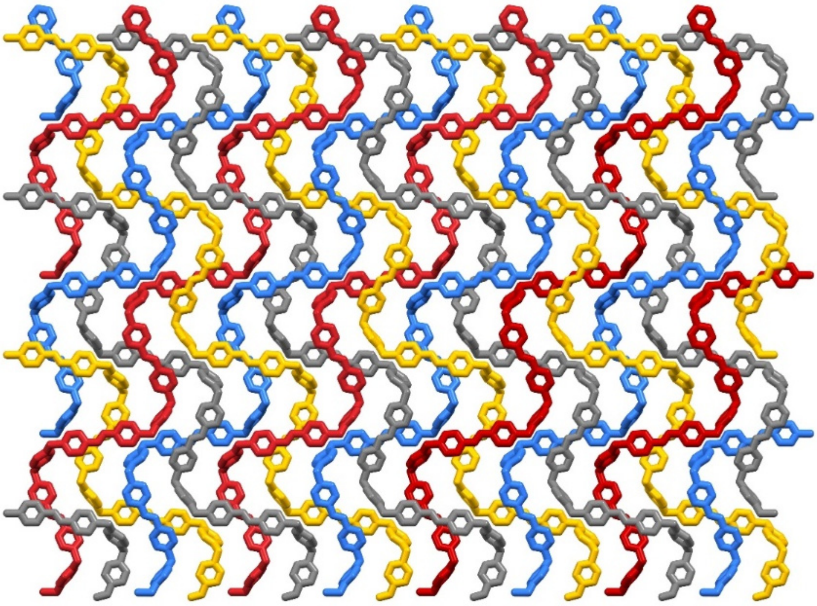

Supplementary Fig. 48. A view of the monolayer of the crystal structure of **2DWPN-2** along *c* axis. The independent chains are distinguished by different colors. H atoms, catechol groups and *para*-xylene molecules were hidden for the sake of presenting a clear woven display.

19. *Characterization of **NWPN-2** crystals*

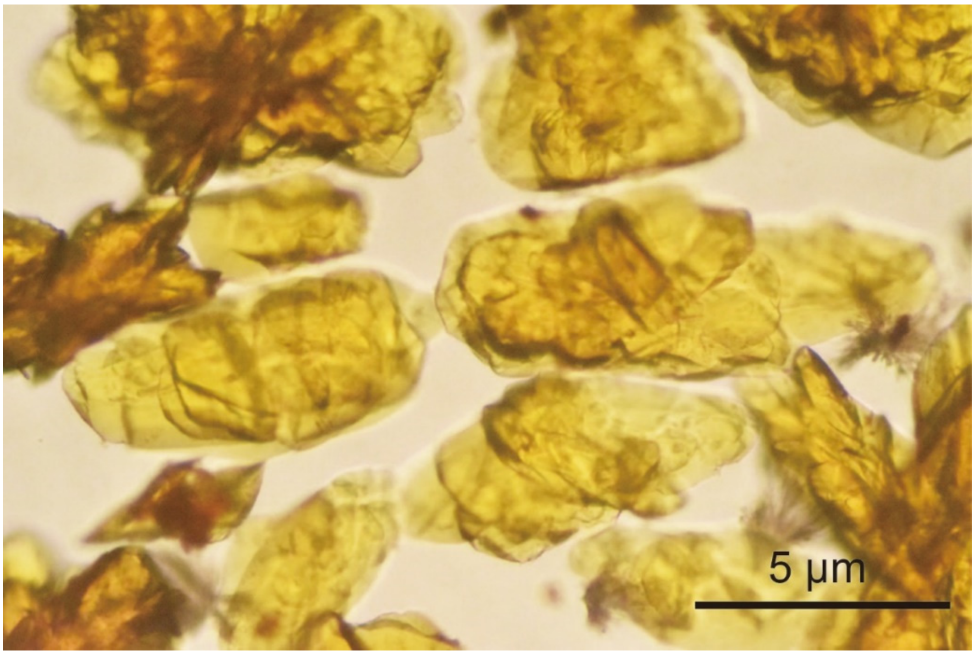

Supplementary Fig. 49. OM Image of **NWPN-2** crystals

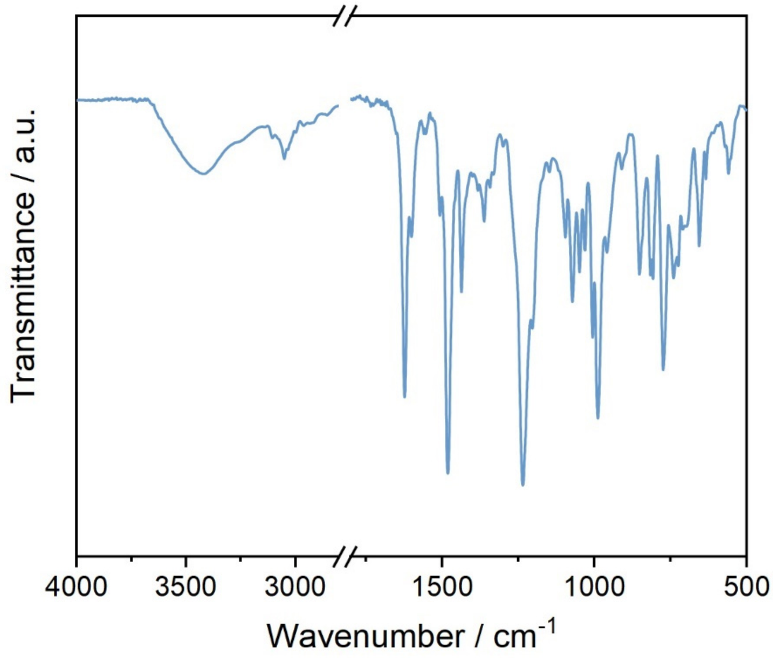

Supplementary Fig. 50. Infrared spectra of **NWPN-2**. Wavenumbers 1483 cm^{-1} and 1235 cm^{-1} correspond to the characteristic absorption peaks of the B−O bond, and 1362 cm^{-1} corresponds to the characteristic absorption peak of the dative B−N bond.

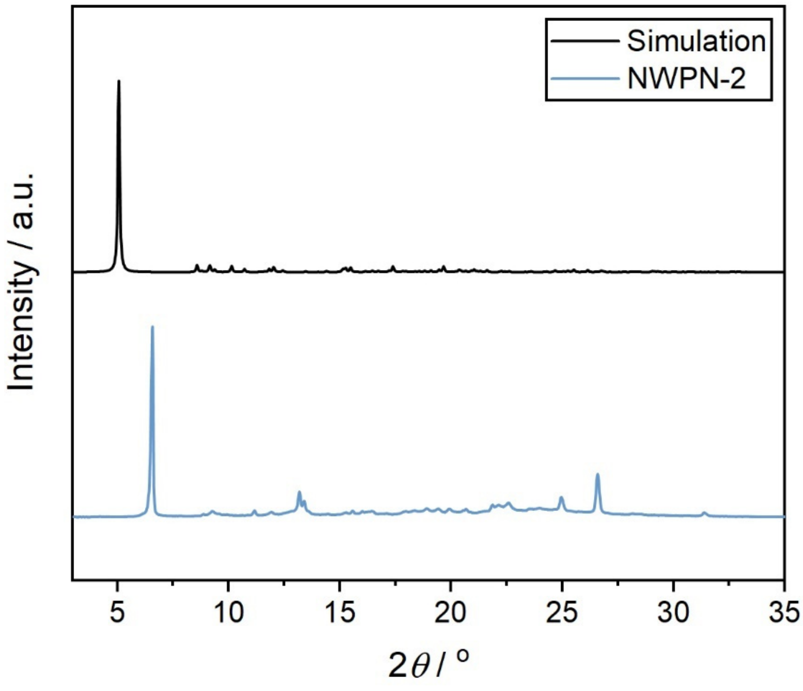

Supplementary Fig. 51. The PXRD patterns of the bulk crystals of **NWPN-2**

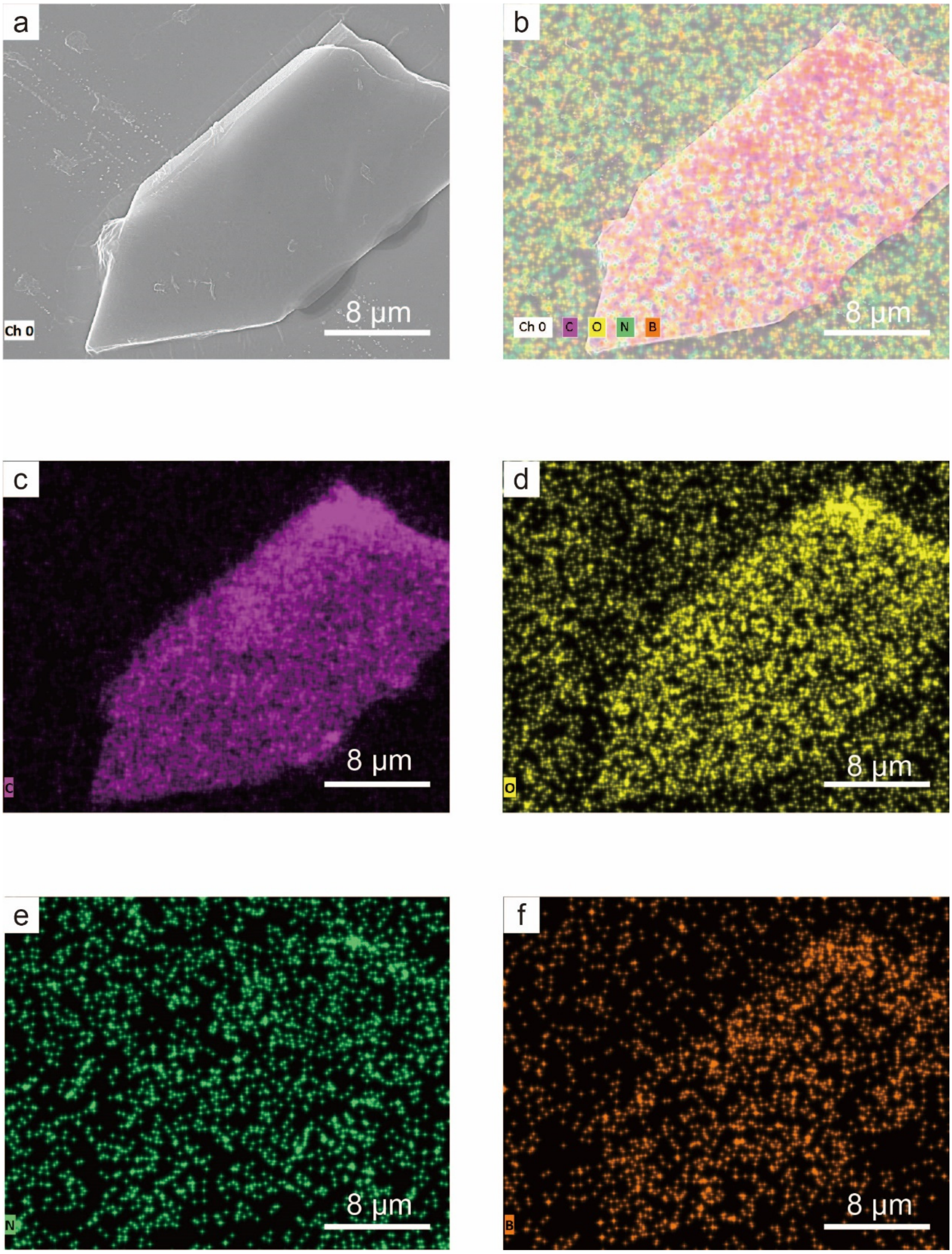

Supplementary Fig. 52. SEM Image (**a**) and the EDS mapping (**b**) of **NWPN-2**. The corresponding elemental maps: carbon (**c**), oxygen (**d**), nitrogen (**e**), and boron (**f**)

20. *Crystal structure of* ***NWPN-2***

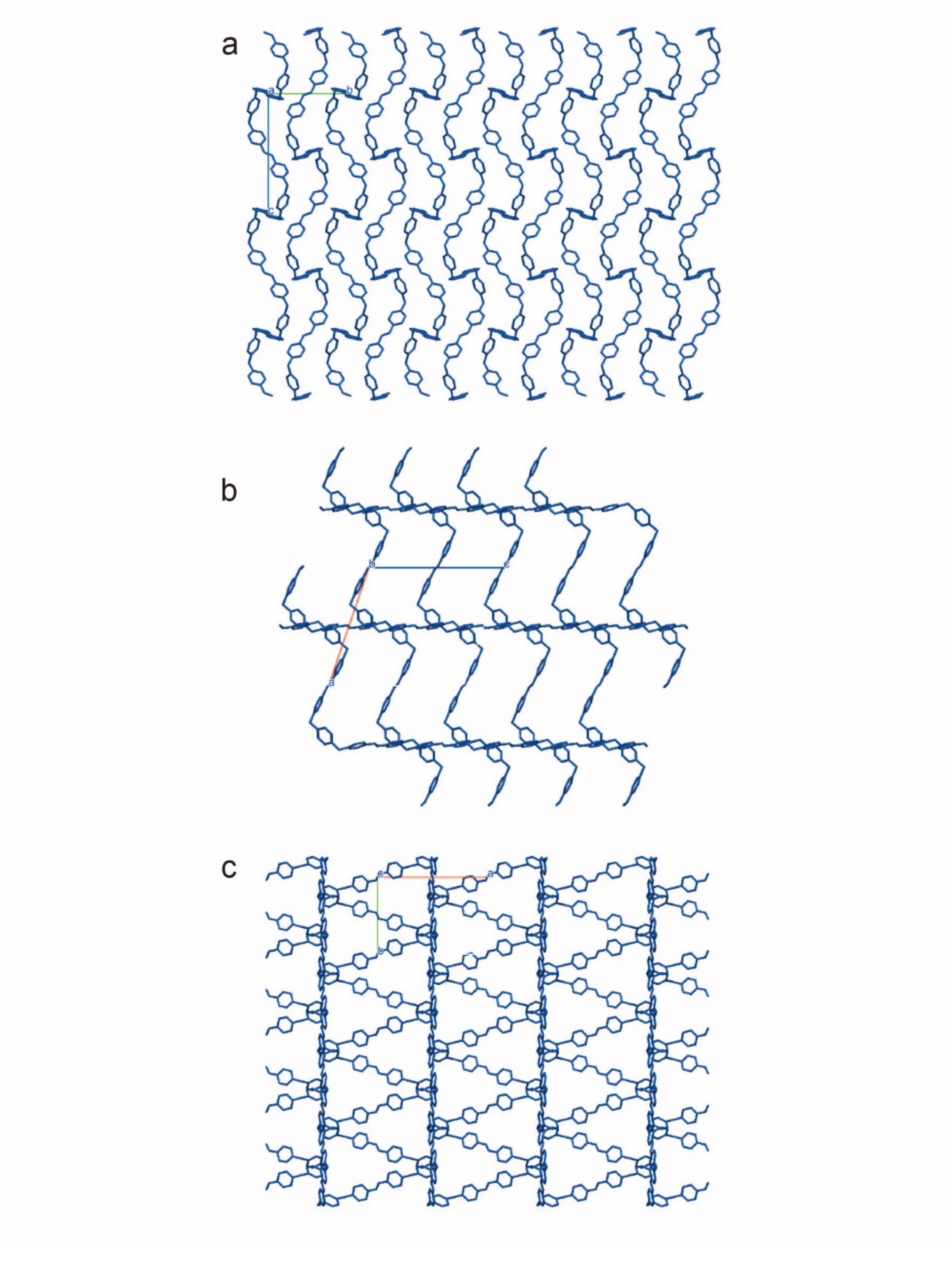

Supplementary Fig. 53. Crystal structure of **NWPN-2**. (**a**, **b**, and **c**) views along the *a*, *b*, and *c* axes. H atoms, catechol groups and *ortho*-xylene molecules were hidden for the sake of providing a clear display.

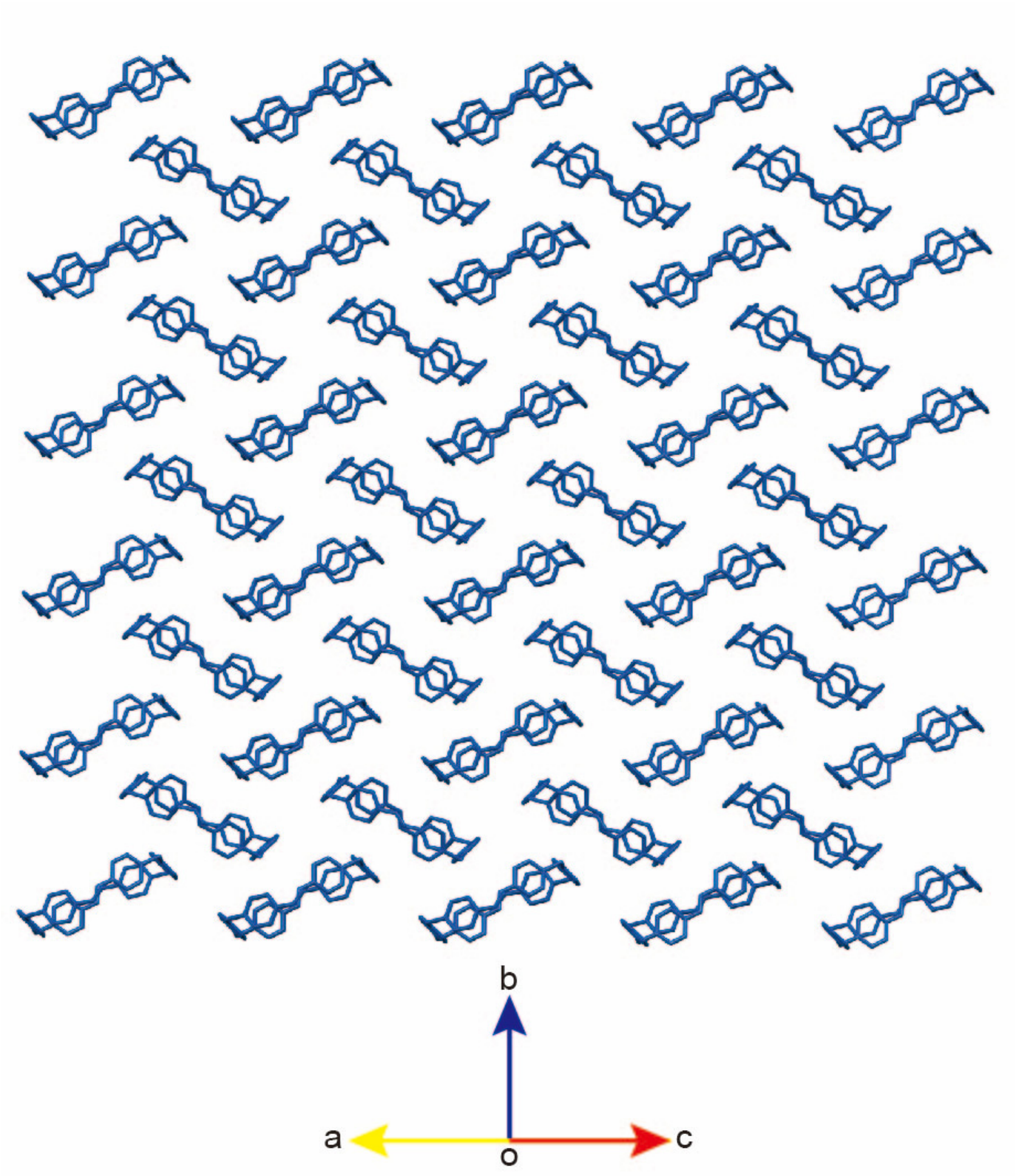

Supplementary Fig. 54. Crystal structure of **NWPN-2**. H atoms, catechol groups and *ortho*-xylene molecules were hidden for the sake of clarity. The crystal structure of **NWPN-2** is formed by parallel stacking of polymer chains.

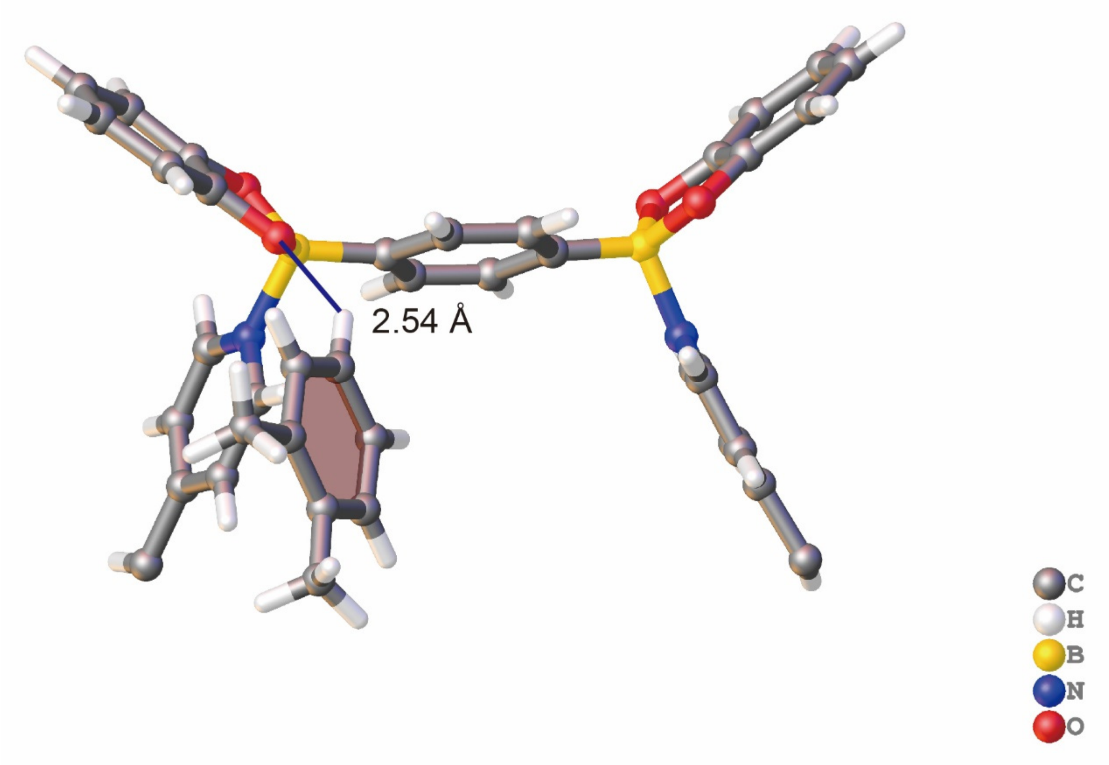

Supplementary Fig. 55. The [C–H···O] interaction between an *ortho*-xylene solvent molecule and a polymer chain in an asymmetric unit of **NWPN-2**

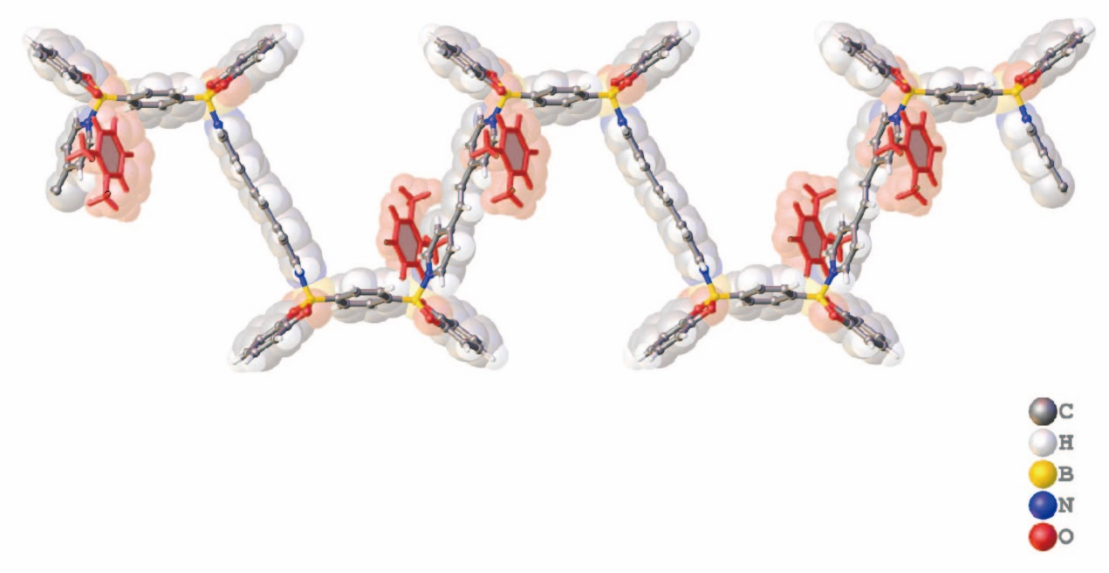

Supplementary Fig. 56. The conformation of a polymer chain in the crystal structure of **NWPN-2**

21. *Theoretical calculations of packing energies for* ***2DWPN-1***

To provide microscopic insight into the directional self-assembly of **2DWPN-1**, molecular simulations were conducted to estimate the packing energies of **2DWPN-1** along the *ab*-plane (in-plane direction) and *c*-axis (out-of-plane direction). Firstly, both model structures created from the X-ray crystal structure of **2DWPN-1** were optimized by the Forcite in Materials Studio[3]. In order to simplify the calculation, a layer of polymer chains in a cell was selected as a unit. The energies of **2DWPN-1** along the *ab*-plane (E_{ab}) and *c*-axis (E_c) as well as a unit (E_{unit}) were calculated, respectively. Then, the packing energies were calculated based on the following equations:

$$\Delta E_{ab} = (E_{ab} - 2E_{\mathrm{unit}})/2 \quad [1]$$

$$\Delta E_{c} = (E_{c} - 2E_{\mathrm{unit}})/2 \quad [2].$$

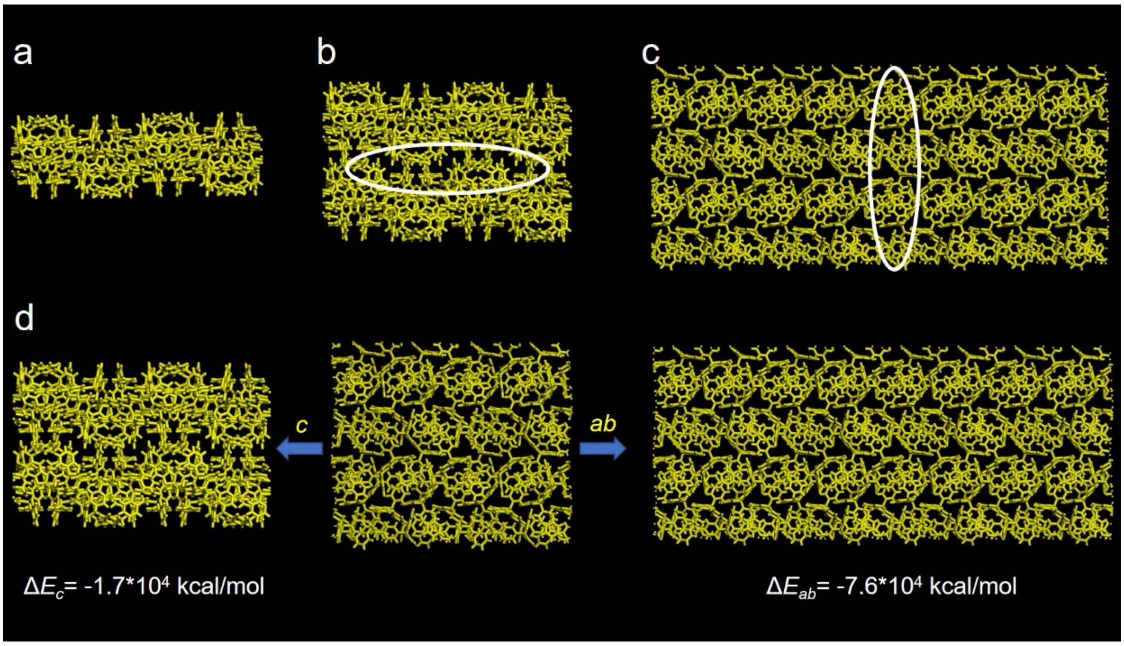

Supplementary Fig. 57. Theoretical calculations of packing energies for **2DWPN-1** along the *ab*-plane (in-plane direction) and *c* axis (out-of-plane direction). (**a**) A layer of polymer chains in a cell is selected as a unit. (**b**) The interactions between two units within the *ab*-plane (*ab*

direction). (**c**) The interactions between two units along the *c* axis (*c* direction). (**d**) Theoretical calculations of packing energies for **2DWPN-1** in the *ab* in-plane direction (ΔE_{ab}) and along *c* out-of-plane direction (ΔE_c) through the molecular simulation.

22. *Optical microscopy images of typical* ***2DWPN-1*** *flakes exfoliated on a* SiO_2*/Si substrate*

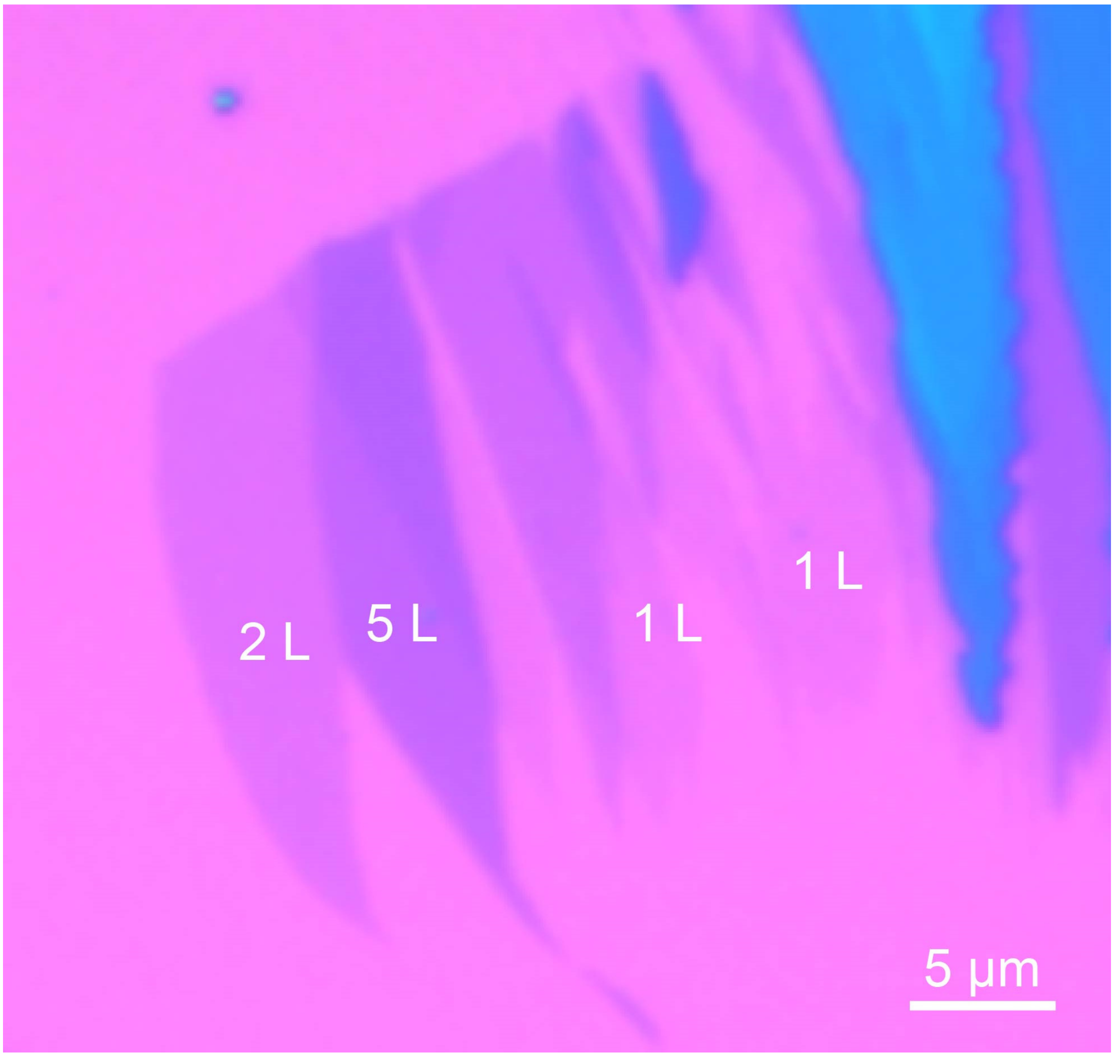

Supplementary Fig. 58. OM Image of typical **2DWPN-1** flakes obtained by the micromechanical exfoliation method. After exfoliation, nanosheets with different thicknesses are obtained on a SiO_2/Si substrate.

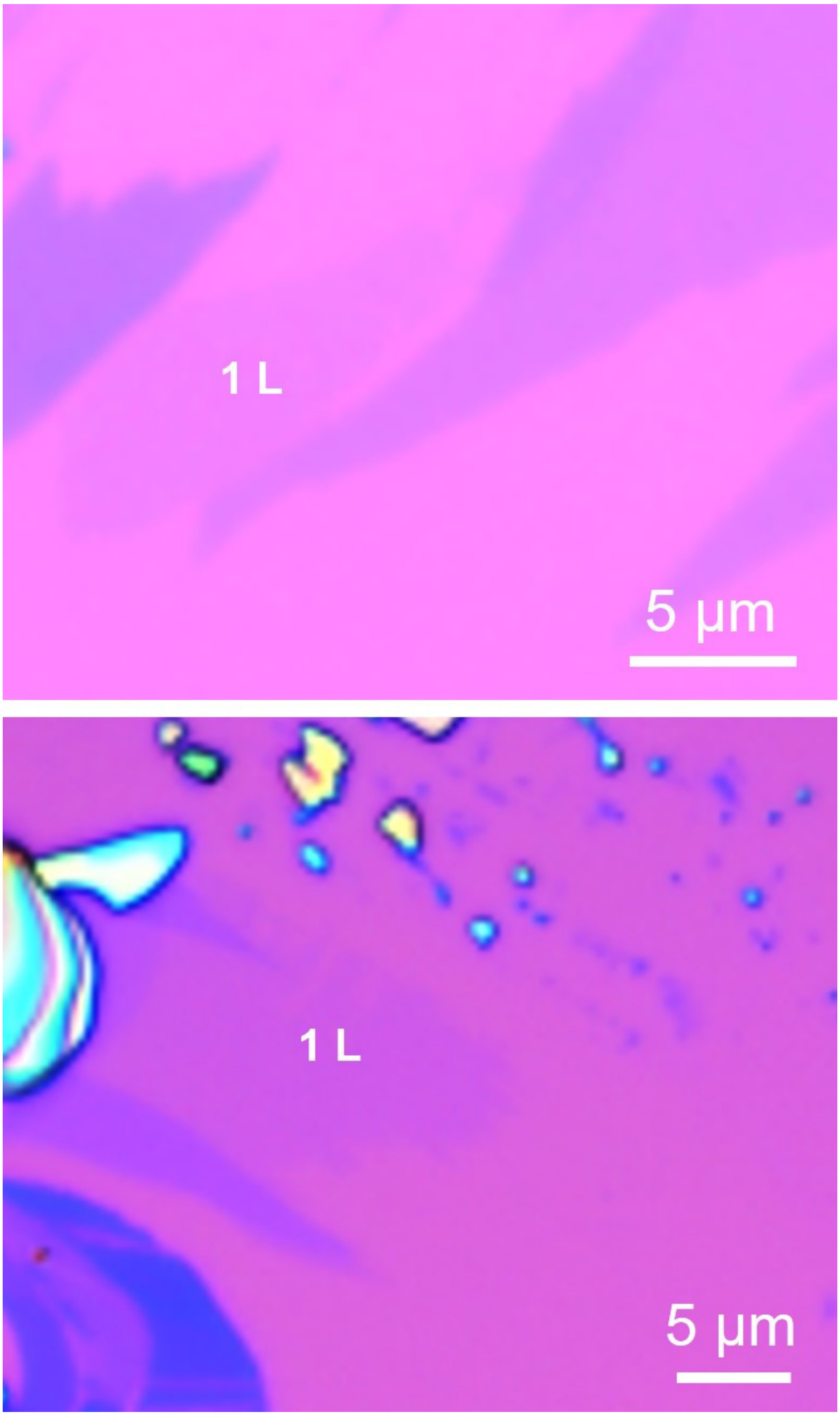

Supplementary Fig. 59. OM Images of large size monolayers of **2DWPN-1** on a SiO_2/Si substrate obtained by the micromechanical exfoliation method. After exfoliation, ultrathin monolayers of **2DWPN-1** were obtained on a SiO_2/Si substrate.

23. *AFM Images of 2D flakes on a SiO2/Si substrate*

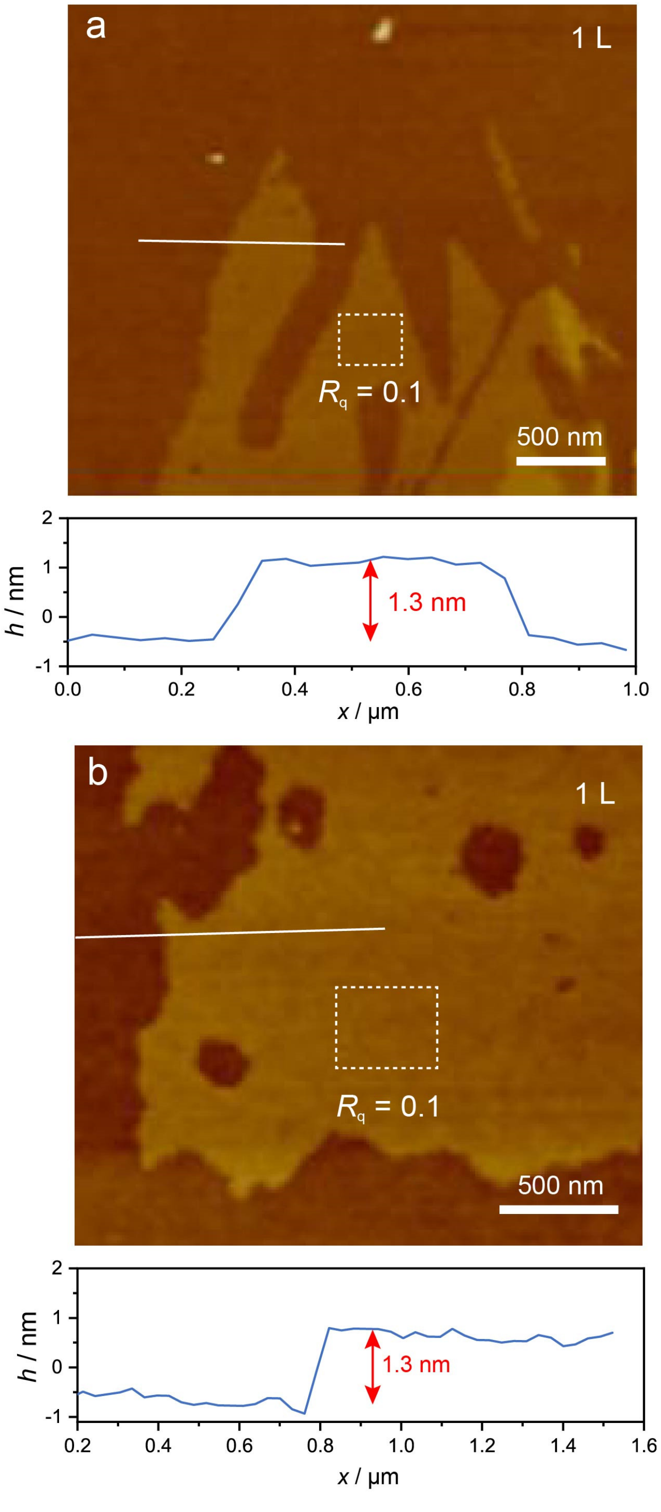

Supplementary Fig. 60. AFM Images of monolayers of **2DWPN-1** on a SiO_2/Si substrate obtained by the micromechanical exfoliation method. The surface roughness (R_q) values of the selected areas in (**a**) and (**b**) are both 0.1.

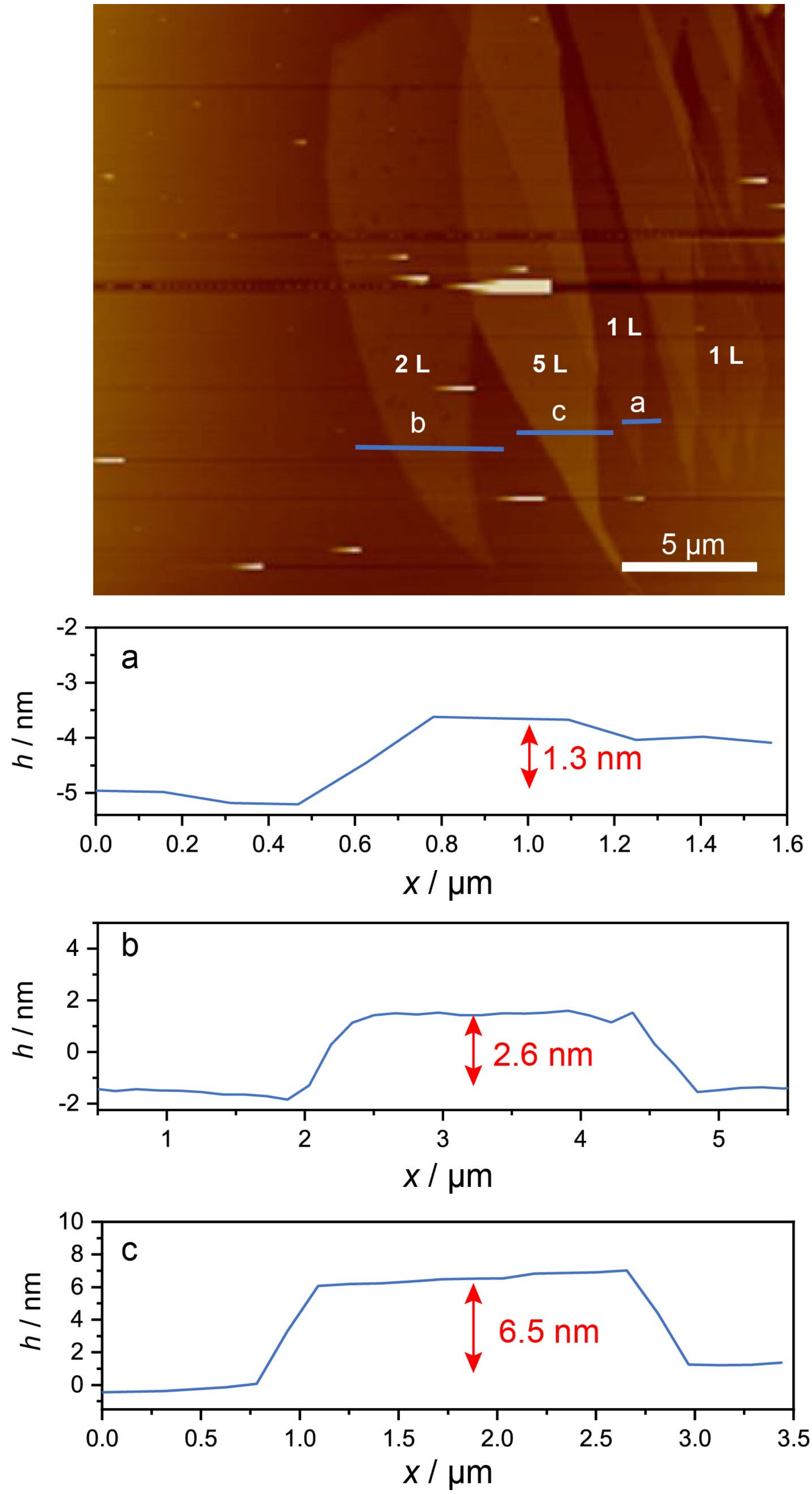

Supplementary Fig. 61. An AFM image of typical **2DWPN-1** flakes, obtained by the micromechanical exfoliation method, with different numbers of layers on a SiO_2/Si substrate

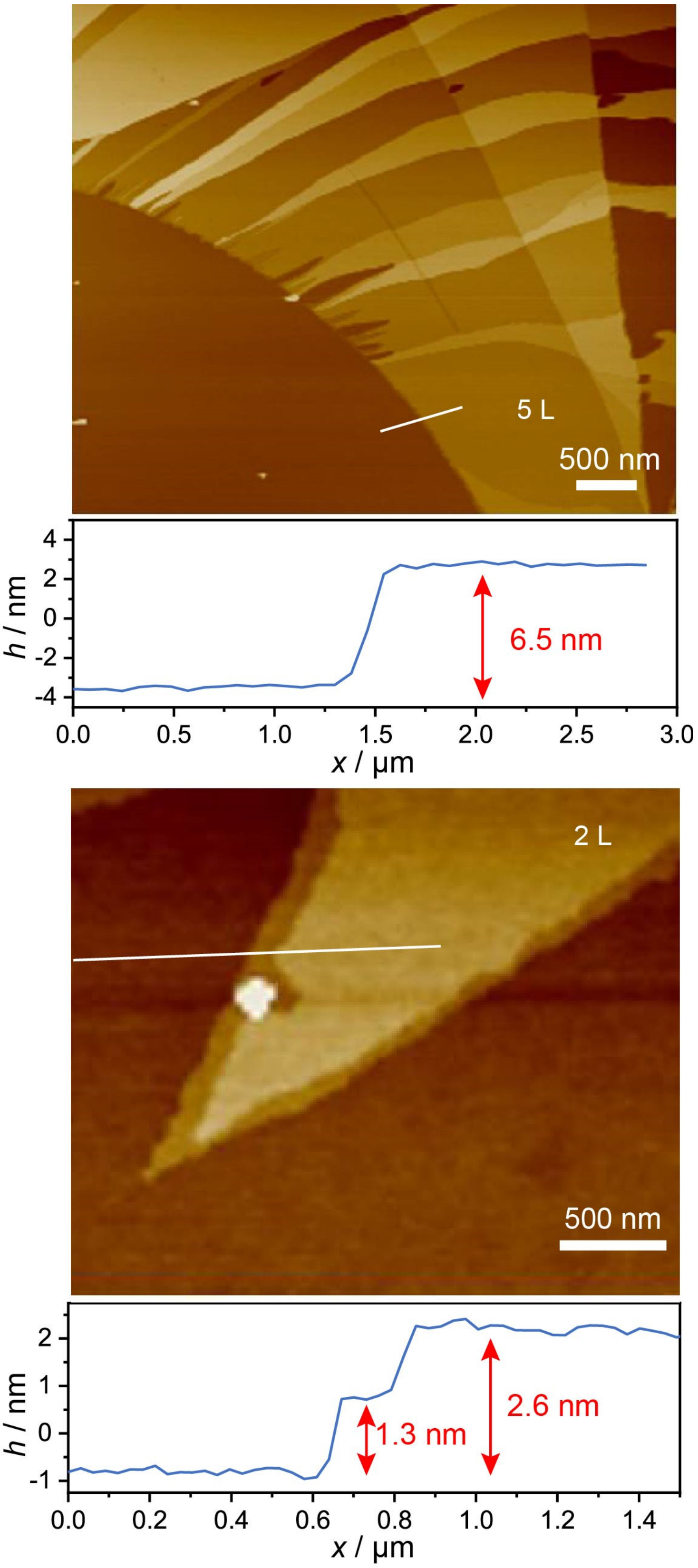

Supplementary Fig. 62. AFM Images of typical **2DWPN-1** flakes, obtained by the micromechanical exfoliation method, with 5 and 2 layers on a SiO_2/Si substrate

24. *AFM Measurements of blank SiO2/Si substrates*

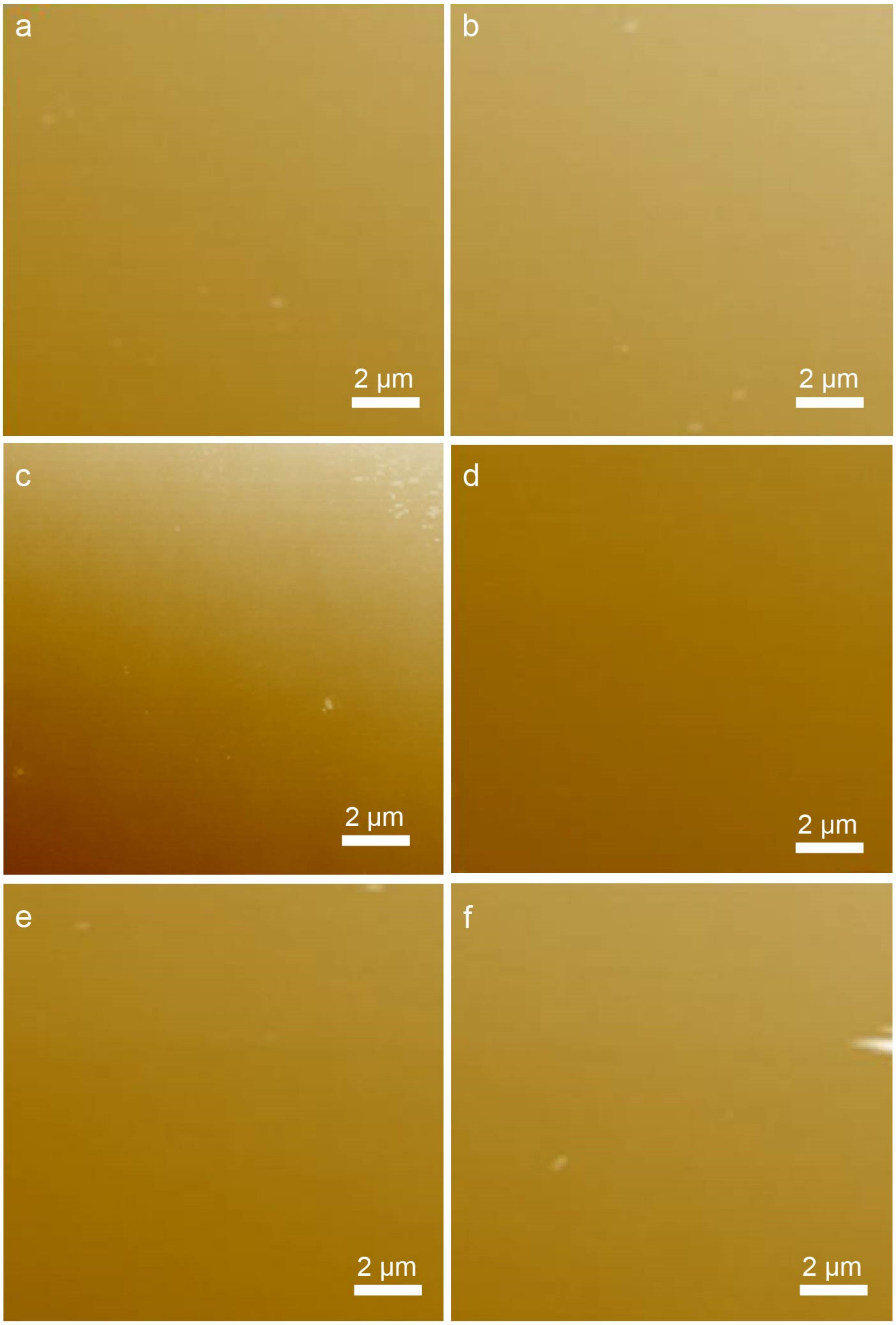

Supplementary Fig. 63. AFM Measurements of blank SiO_2/Si substrates. AFM Images of blank SiO_2/Si substrates used for deposition of the molecular nanosheets of the obtained crystals after pre-treatment. No nanosheets of crystals are observed on blank substrates.

25. *AFM of as-exfoliated **2DWPN-1** nanosheets obtained by liquid exfoliation*

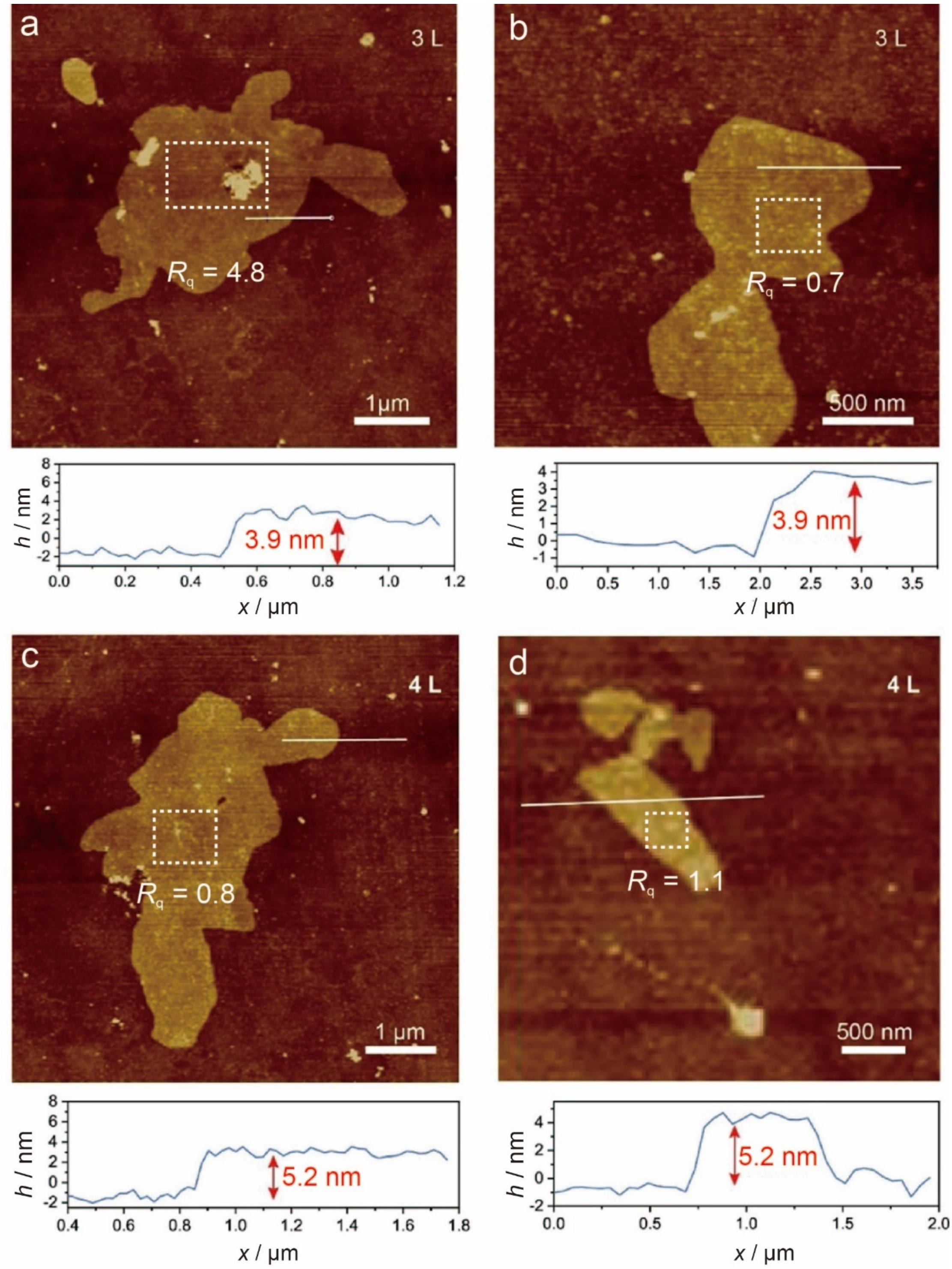

Supplementary Fig. 64. AFM Images of typical **2DWPN-1** nanosheets with 3 and 4 layers on a SiO_2/Si substrate obtained by liquid exfoliation. The surface roughness (R_q) values of the selected areas in (**a**), (**b**), (**c**) and (**d**) are 4.8, 0.7, 0.8 and 1.1, respectively.

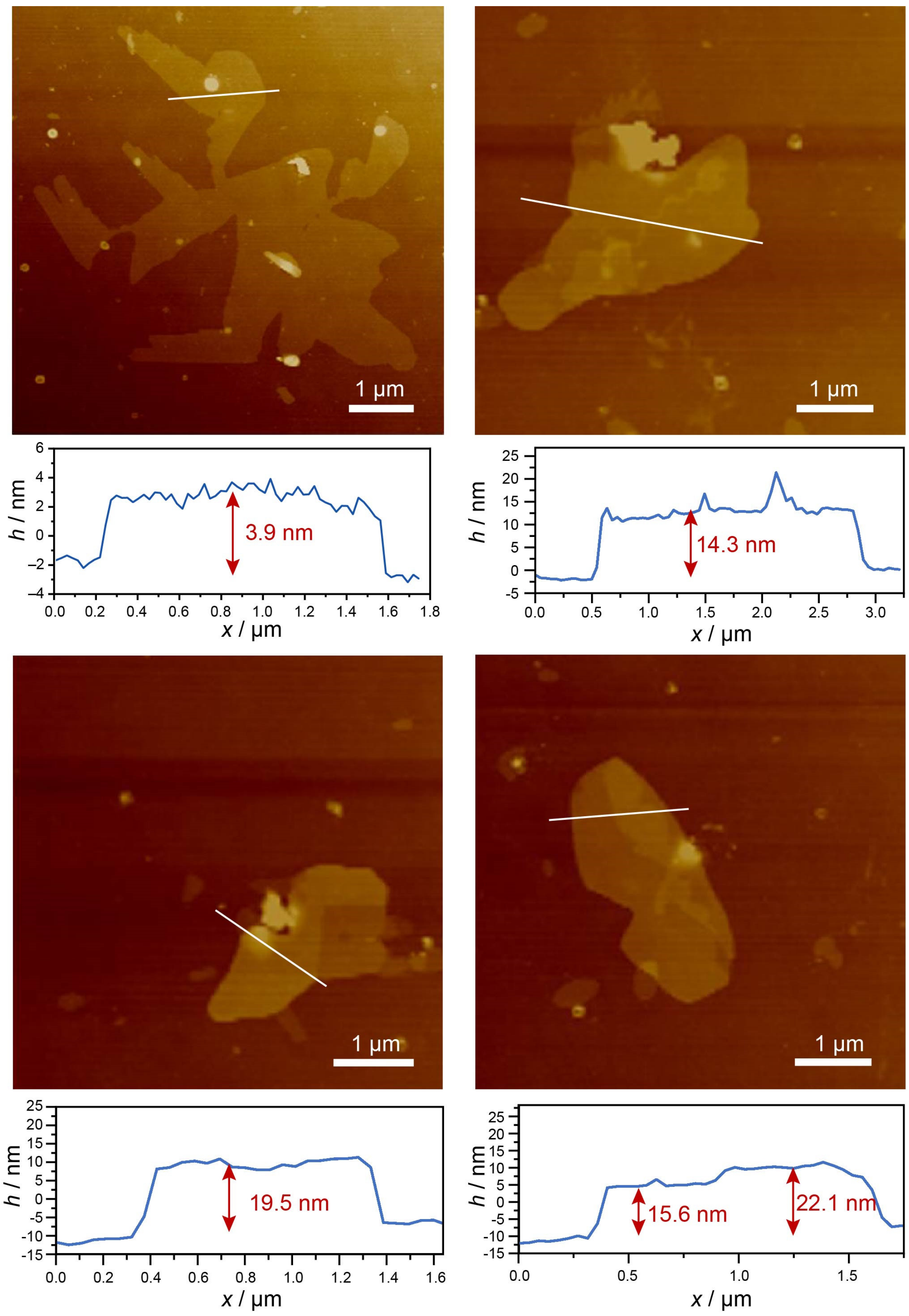

Supplementary Fig. 65. AFM Images of typical **2DWPN-1** flakes on a SiO_2/Si substrate obtained by liquid exfoliation

26. *AFM Measurements of the particles obtained from* ***NWPN-1*** *bulk crystals after ultrasonication*

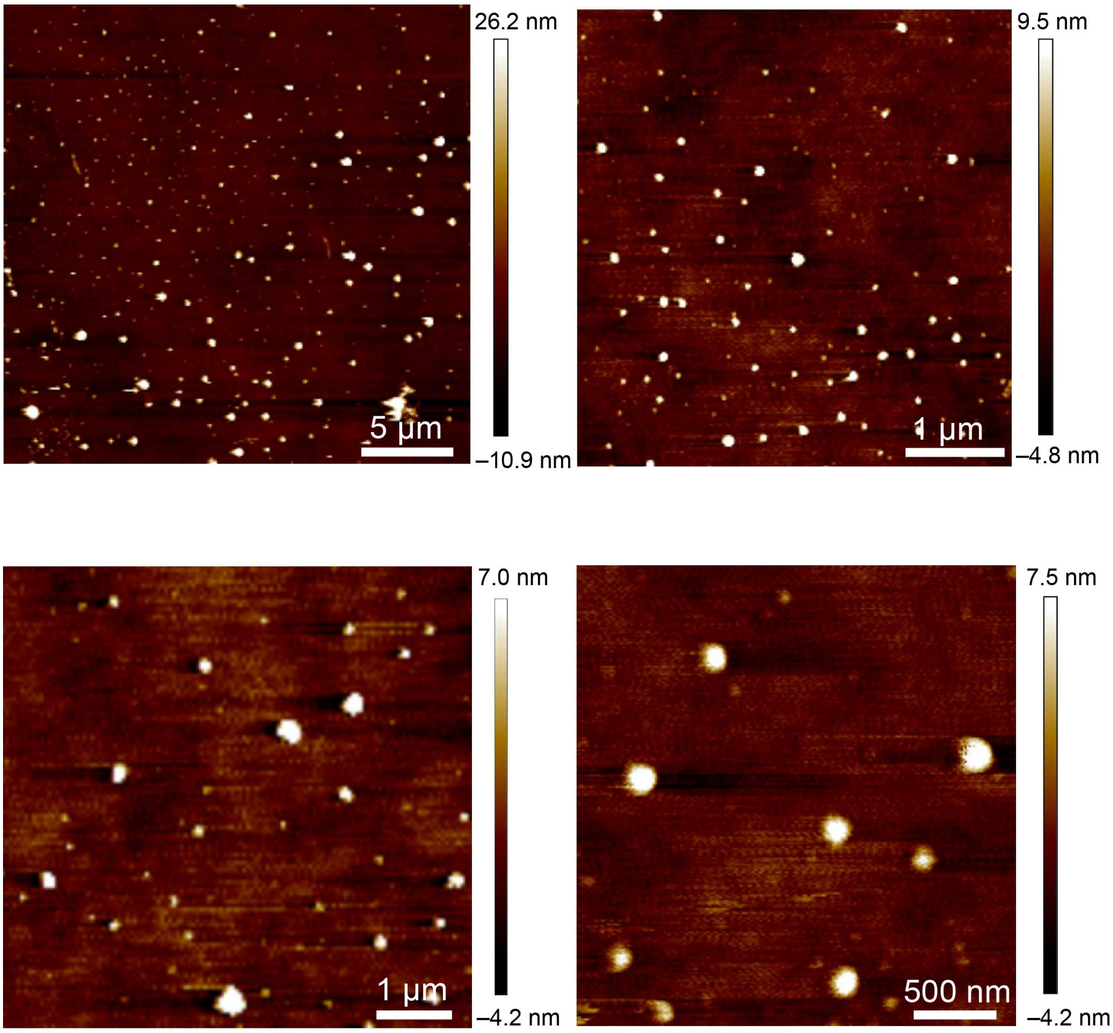

Supplementary Fig. 66. AFM Measurements of the particles obtained from **NWPN-1** bulk crystals after ultrasonication. Here the crystals of **NWPN-1** were grown in *meta*-xylene.

27. *Simulation of Raman peaks of* ***2DWPN-1***

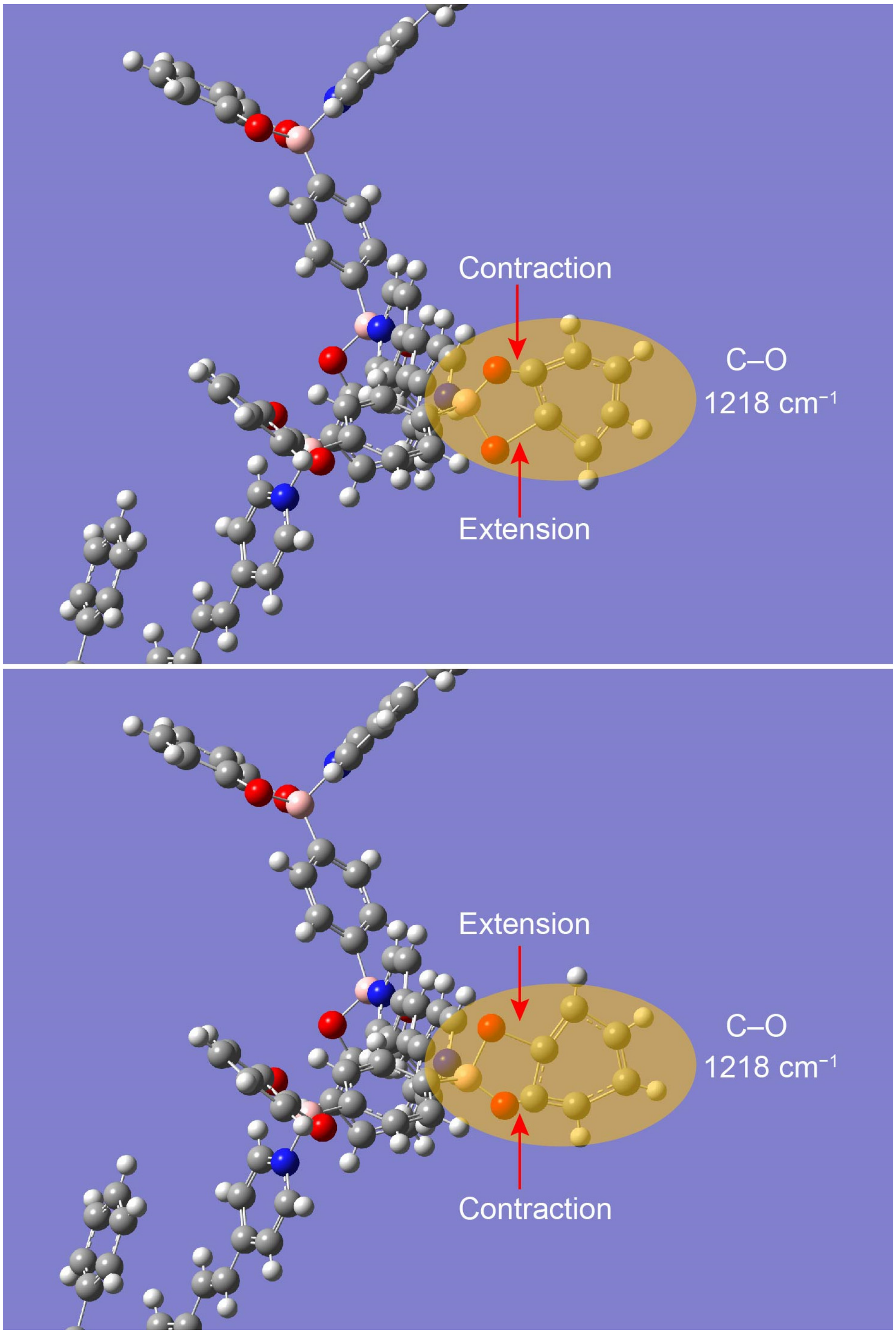

Supplementary Fig. 67. Simulation of the Raman peak (1218 cm^{-1}) of the C−O bond of **2DWPN-1** from DFT calculations. All these calculations are performed using the Gaussian 09 software package, employing the B3LYP /6-311G (d, p) level of theory[2].

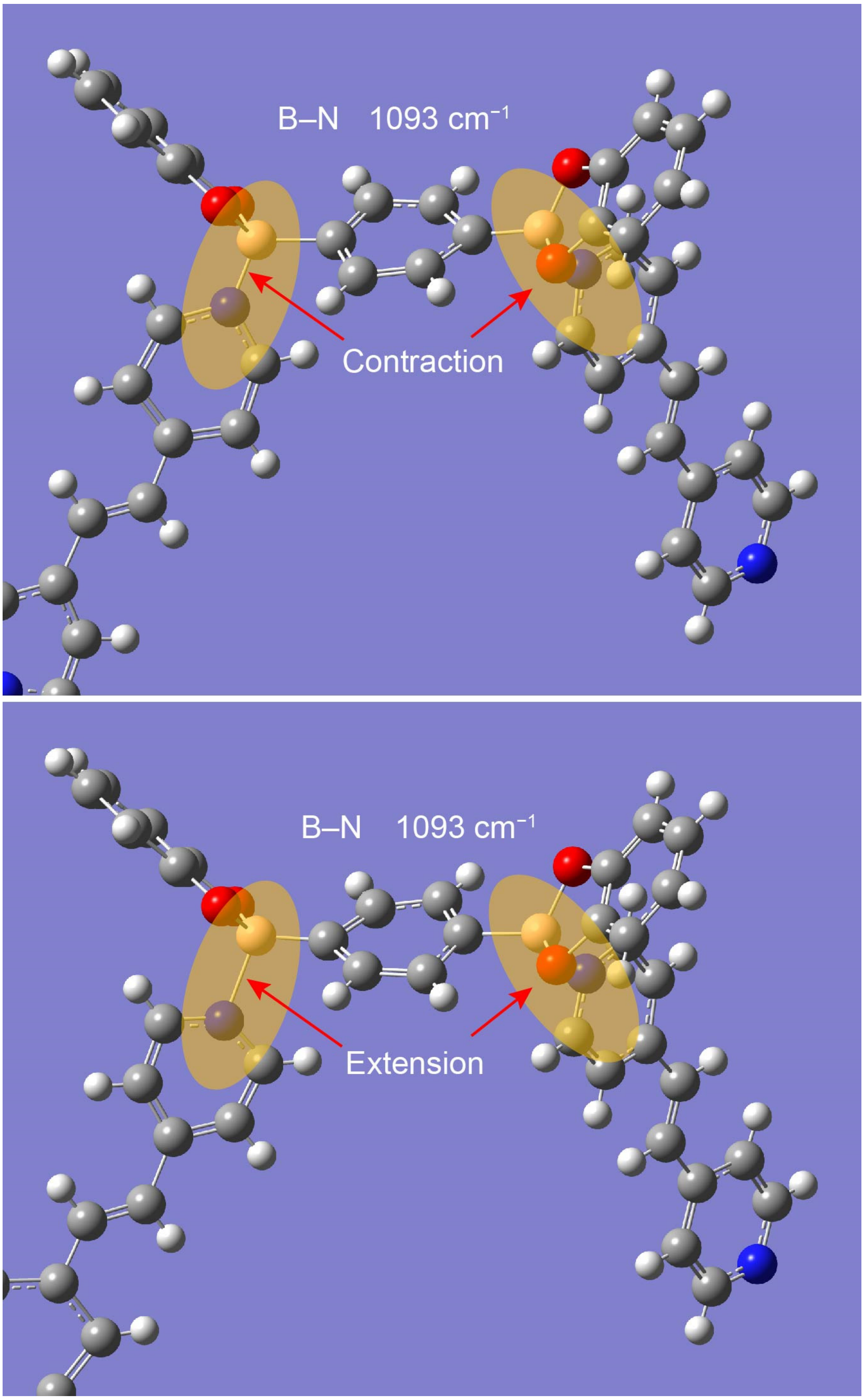

Supplementary Fig. 68. Simulation of the Raman peak (1093 cm^{-1}) of the dative B−N bond of **2DWPN-1** from DFT calculations

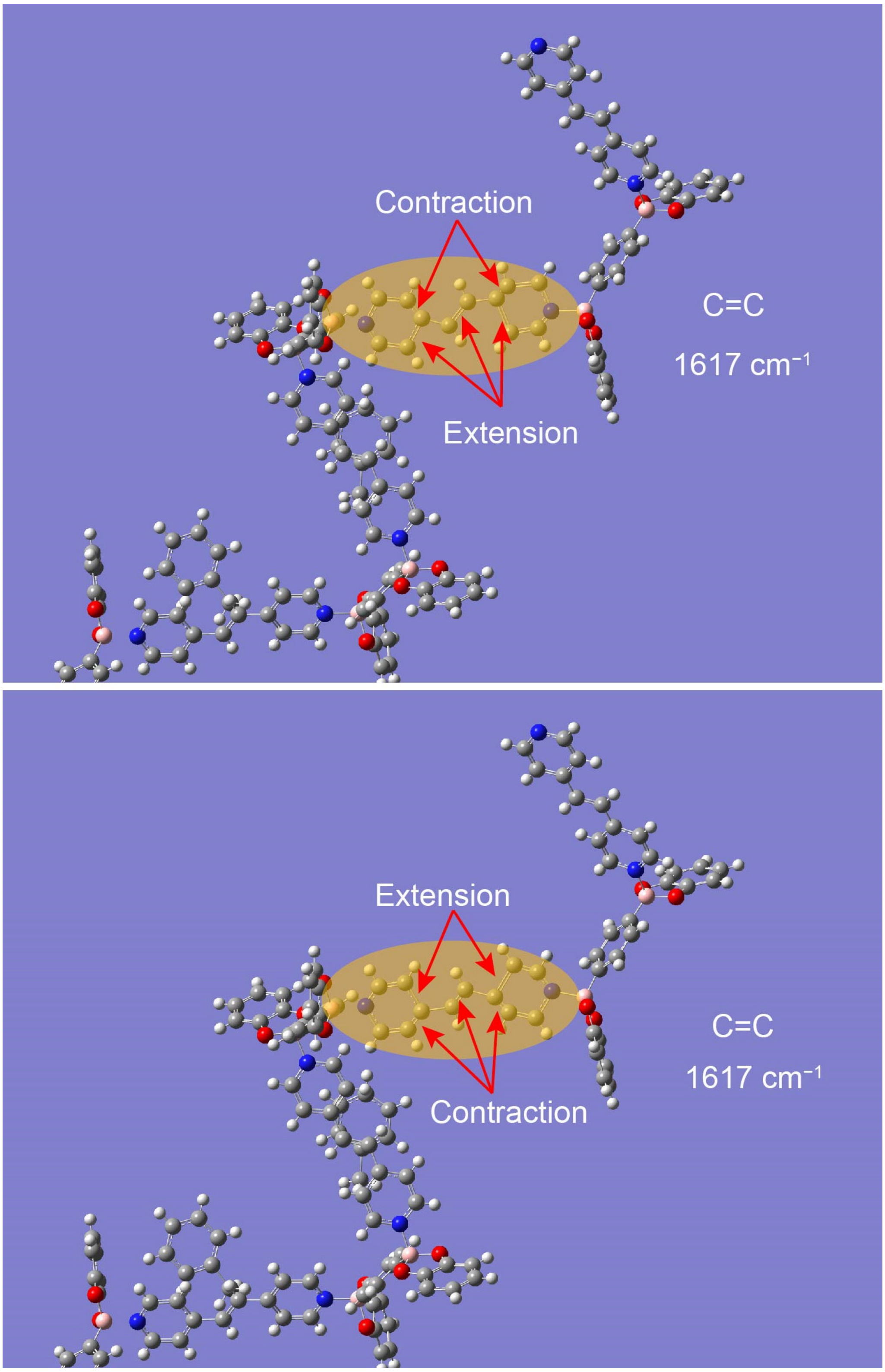

Supplementary Fig. 69. Simulation of the Raman peak (1617 cm^{-1}) of the C=C bond of **2DWPN-1** from DFT calculations

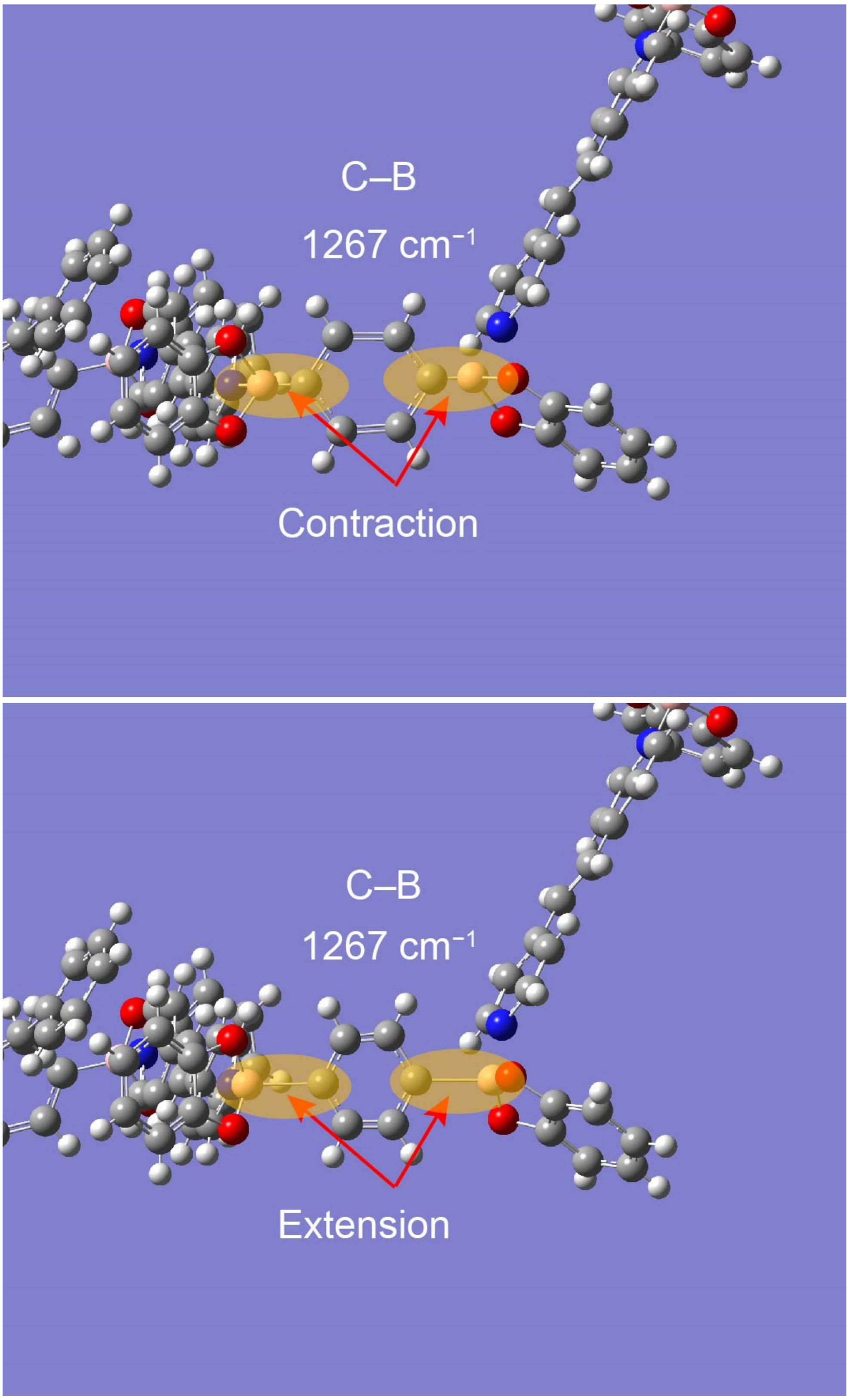

Supplementary Fig. 70. Simulation of the Raman peak (1267 cm^{-1}) of the C−B bond of **2DWPN-1** from DFT calculations

28. *TEM Analysis of* ***2DWPN-1***

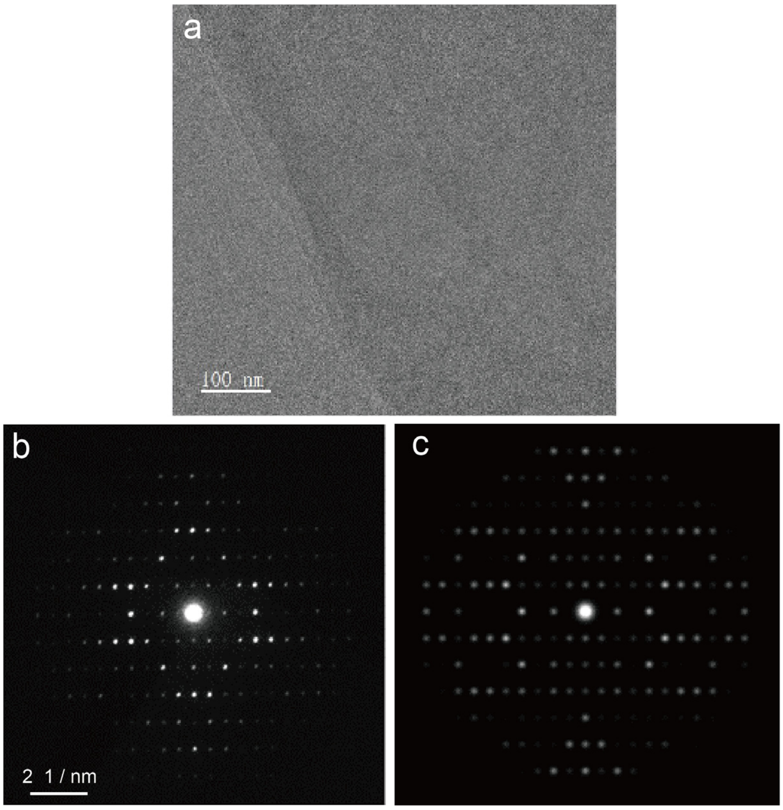

Supplementary Fig. 71. (**a**) TEM Image of an as-exfoliated nanosheet of **2DWPN-1**, and (**b**) the corresponding selected area electron diffraction pattern. (**c**) The simulated electron diffraction pattern of **2DWPN-1**.

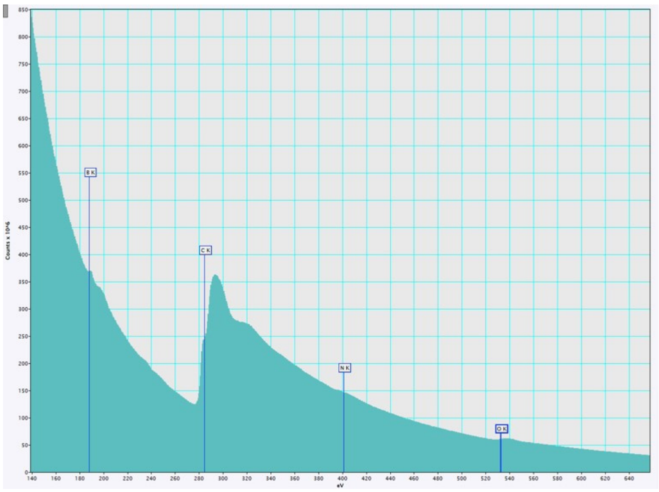

Supplementary Fig. 72. The electron energy loss spectrum of an as-exfoliated nanosheet of **2DWPN-1**

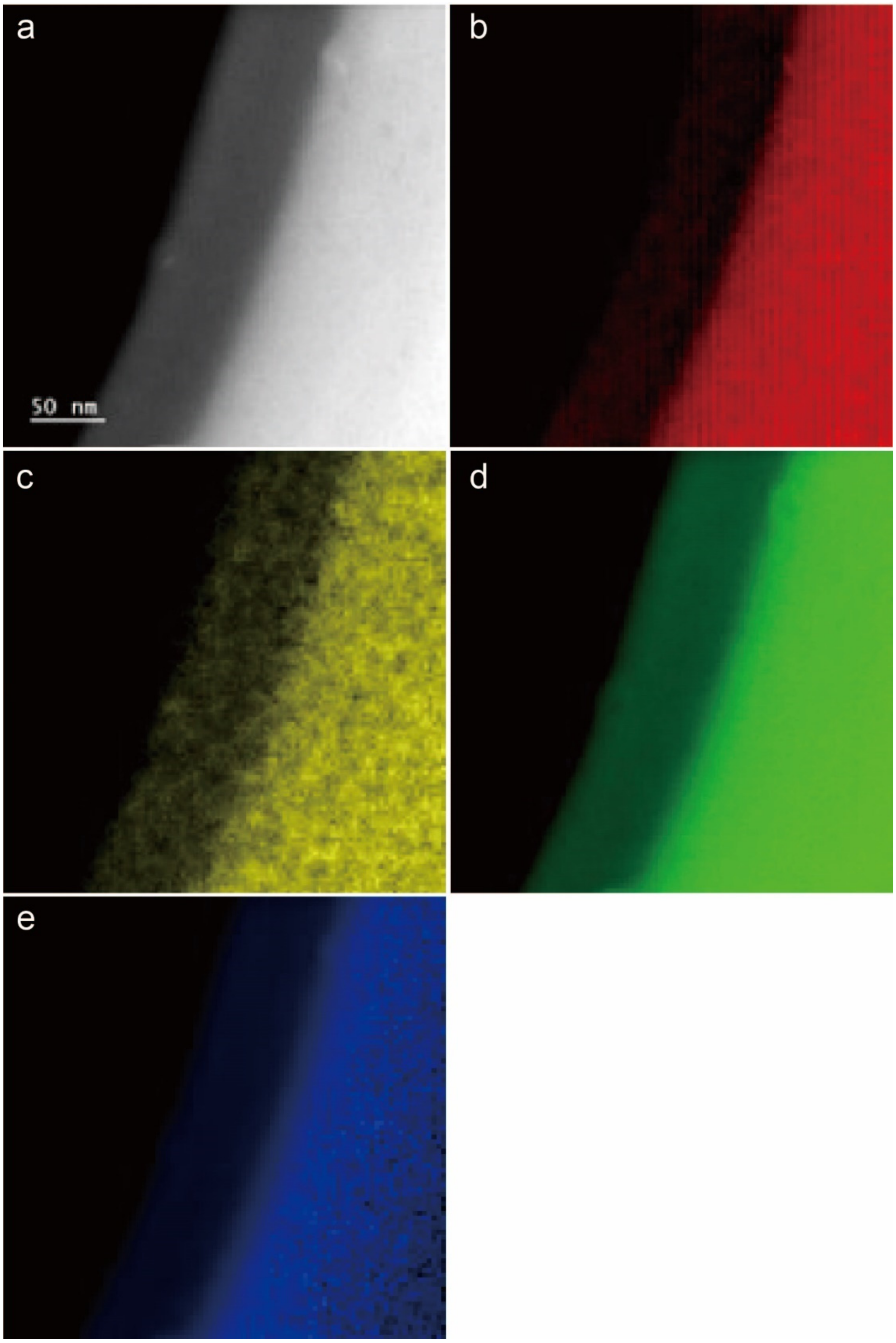

Supplementary Fig. 73. TEM Image (**a**) and the corresponding electron energy loss spectroscopy elemental mapping of (**b**) boron, (**c**) oxygen, (**d**) carbon and (**e**) nitrogen elements on an as-exfoliated nanosheet of **2DWPN-1**

29. *Nanoindentation studies on crystals of 2DWPN-1 and NWPN-1*

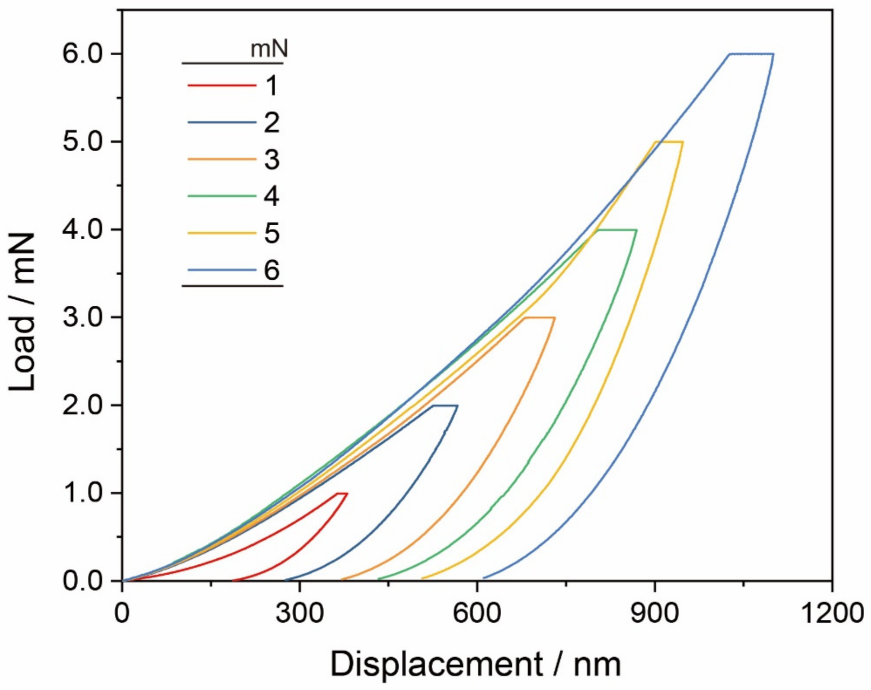

Supplementary Fig. 74. Nanoindentation studies on a crystal of **NWPN-1**. Representative load-depth (*P*–*h*) curves were obtained using a load-controlled mode of different loads from 1 to 6 mN.

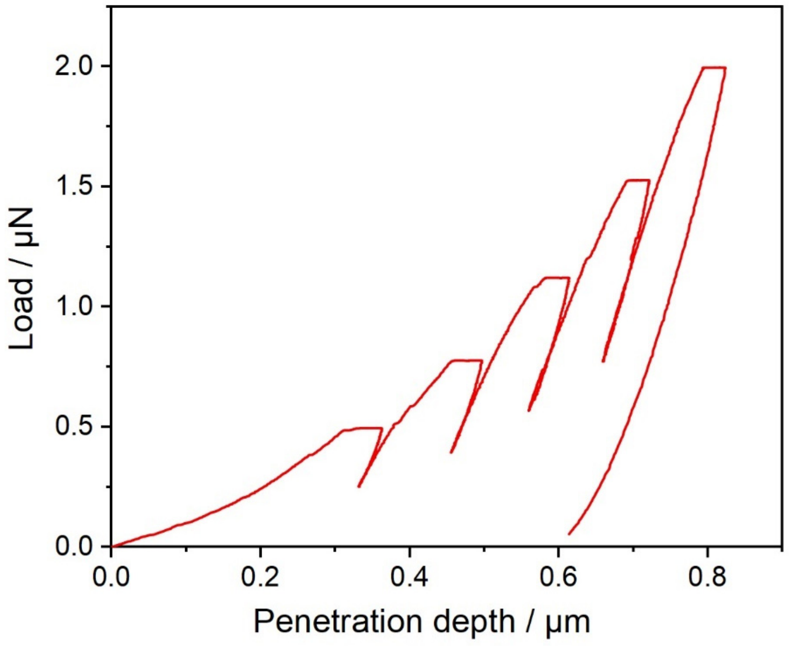

Supplementary Fig. 75. Nanoindentation studies on a crystal of **2DWPN-1**. Representative preliminary load-depth (*P*–*h*) traces for multi-load nanoindentation on this crystal

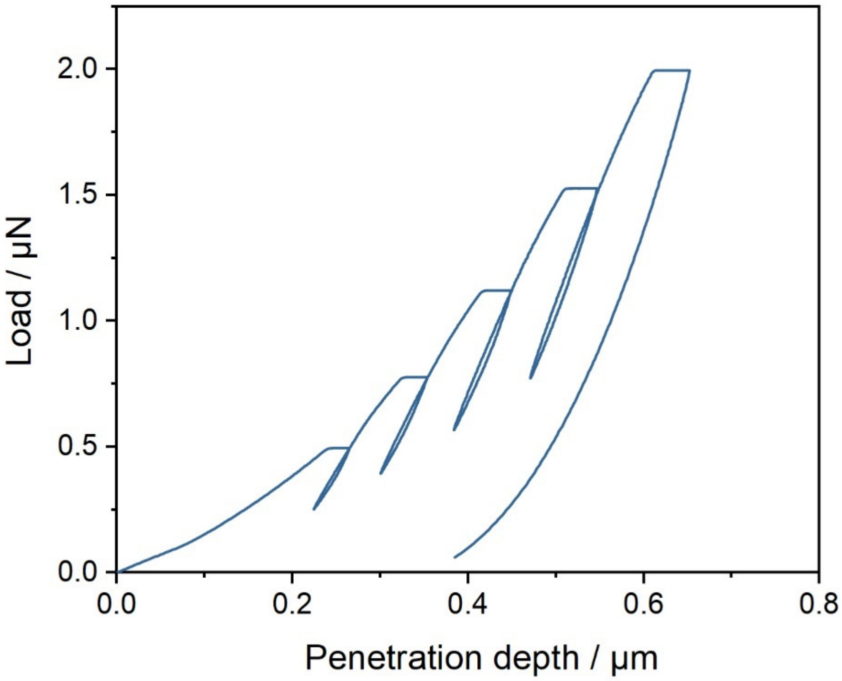

Supplementary Fig. 76. Nanoindentation studies on a crystal of **NWPN-1**. Representative preliminary load-depth (*P*–*h*) traces for multi-load nanoindentation on this crystal

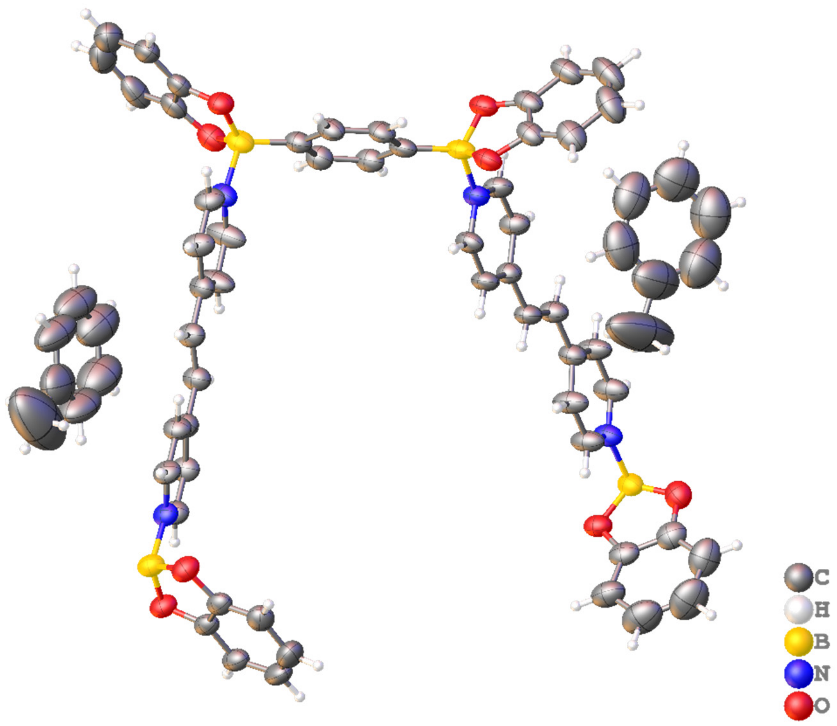

Supplementary Fig. 77. ORTEP-style illustration of **2DWPN-1** (CCDC 2238613)

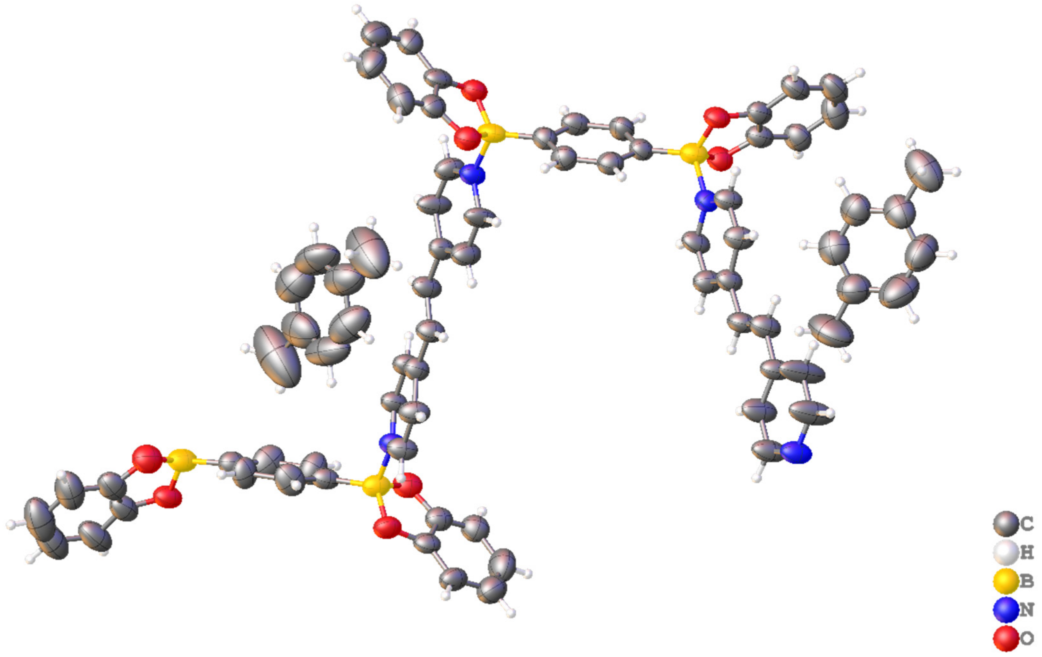

Supplementary Fig. 78. ORTEP-style illustration of **2DWPN-2** (CCDC 2238614)

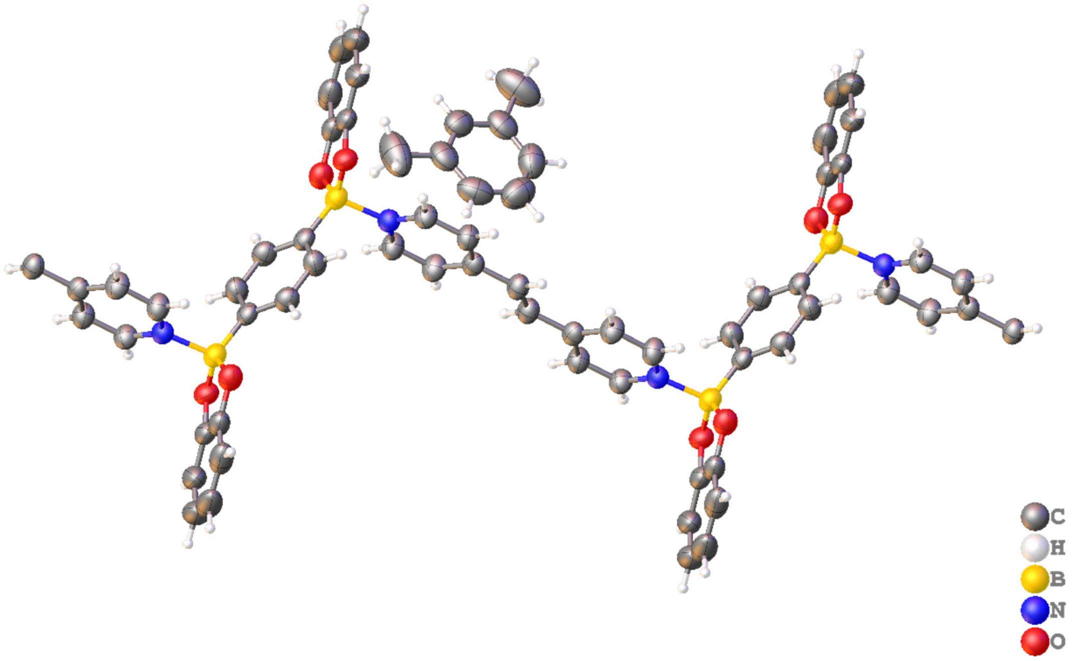

Supplementary Fig. 79. ORTEP-style illustration of **NWPN-1** (CCDC 2238615)

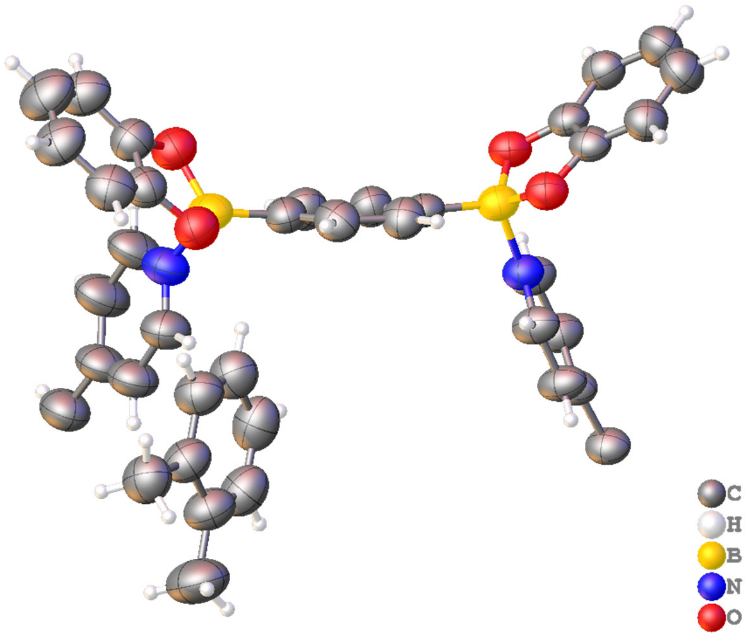

Supplementary Fig. 80. ORTEP-style illustration of **NWPN-2** (CCDC 2238616)

Supplementary Table 1| *Crystallographic data and structural refinement summary of* ***2DWPN-1***

Compounds	**2DWPN-1**
CCDC	2238613
Empirical formula	$C_{60}H_{44}B_4N_4O_8 \cdot 2(C_7H_8)$
Temperature (K)	213
Formula weight	1176.50
Crystal system	Orthorhombic
Space group	P_{bca}
a (Å)	23.4671 (6)
b (Å)	19.6536 (5)
c (Å)	28.7688 (5)
α (°)	90
β (°)	90
γ (°)	90
Volume (Å^3)	13268.5 (6)
Z	8
ρ_{calc} (g cm^{-3})	1.178
μ (mm^{-1})	0.38
F(000)	4928
Crystal size (mm^3)	0.07 × 0.07 × 0.05
Radiation	Ga Kα, λ = 1.34139 Å
Goodness-of-fit on F^2	1.013
Final R indexes[$I \geq 2\sigma(I)$][(a)]	R_1 = 0.0787<br>wR_2 = 0.2346
Final R indexes [all data][(a)]	R_1 = 0.1350<br>wR_2 = 0.2005

(a) $R_1 = \Sigma||F_o|-|F_c||/\Sigma|F_o|$, $wR_2 = \{\Sigma[w(F_o^2-F_c^2)^2]/\Sigma[w(F_o^2)^2]\}^{1/2}$

Supplementary Table 2| *Crystallographic data and structural refinement summary of NWPN-1*

Compounds	**NWPN-1**
CCDC	2238615
Empirical formula	$C_{30}H_{22}B_2N_2O_4 \cdot C_8H_{10}$
Temperature (K)	221
Formula weight	602.27
Crystal system	Monoclinic
Space group	$C_{2/c}$
a (Å)	15.2669 (2)
b (Å)	16.6354 (2)
c (Å)	13.7304 (2)
α (°)	90
β (°)	113.2110 (10)
γ (°)	90
Volume (Å^3)	3204.87 (8)
Z	4
ρ_{calc} (g cm^{-3})	1.256
μ (mm^{-1})	0.41
F(000)	1264
Crystal size (mm^3)	0.07 × 0.07 × 0.05
Radiation	Ga Kα, λ = 1.34139 Å
Goodness-of-fit on F^2	1.054
Final R indexes[$I \geq 2\sigma(I)$][(a)]	R_1 = 0.0510<br>wR_2 = 0.1498
Final R indexes [all data][(a)]	R_1 = 0.0649<br>wR_2 = 0.1385

(a) $R_1 = \Sigma||F_o|-|F_c||/\Sigma|F_o|$, $wR_2 = \{\Sigma[w(F_o^2-F_c^2)^2]/\Sigma[w(F_o^2)^2]\}^{1/2}$

Supplementary Table 3| *Crystallographic data and structural refinement summary of **2DWPN-2***

Compounds	**2DWPN-2**
CCDC	2238614
Empirical formula	$C_{60}H_{44}B_4N_4O_8{\cdot}2(C_8H_{10})$
Temperature (K)	213
Formula weight	1204.55
Crystal system	Orthorhombic
Space group	P_{bca}
a (Å)	23.4319 (12)
b (Å)	19.7445 (12)
c (Å)	30.0301 (18)
α (°)	90
β (°)	90
γ (°)	90
Volume (Å^3)	13893.5 (14)
Z	8
ρ_{calc} (g cm^{-3})	1.152
μ (mm^{-1})	0.37
F(000)	5056
Crystal size (mm^3)	0.03 × 0.02 × 0.01
Radiation	Ga $K\alpha$, λ = 1.34139 Å
Goodness-of-fit on F^2	0.966
Final R indexes[$I \geq 2\sigma(I)$][(a)]	R_1 = 0.1010<br>wR_2 = 0.3358
Final R indexes [all data][(a)]	R_1 = 0.2732<br>wR_2 =0.2433

(a) $R_1 = \Sigma||F_o|-|F_c||/\Sigma|F_o|$, $wR_2 = \{\Sigma[w(F_o^2-F_c^2)^2]/\Sigma[w(F_o^2)^2]\}^{1/2}$

Supplementary Table 4| *Crystallographic data and structural refinement summary of NWPN-2*

Compounds	**NWPN-2**
CCDC	2238616
Empirical formula	$C_{30} H_{22} B_2 N_2 O_4 C_8 H_{10}$
Temperature (K)	193
Formula weight	602.27
Crystal system	Monoclinic
Space group	$P2_{1/c}$
a (Å)	18.4332 (17)
b (Å)	12.1984 (14)
c (Å)	19.9224 (14)
α (°)	90
β (°)	108.850 (7)
γ (°)	90
Volume (Å^3)	4274.3 (7)
Z	4
ρ_{calc} (g cm^{-3})	0.936
μ (mm^{-1})	0.476
F(000)	1264.0
Crystal size (mm^3)	0.13 × 0.12 × 0.11
Radiation	Cu $K\alpha$, λ = 1.54189 Å
Goodness-of-fit on F^2	1.025
Final R indexes[$I \geq 2\sigma(I)$][(a)]	$R_1 = 0.0970$<br>$wR_2 = 0.2795$
Final R indexes [all data][(a)]	$R_1 = 0.1373$<br>$wR_2 = 0.3278$

(a) $R_1 = \Sigma||F_o|-|F_c||/\Sigma|F_o|$, $wR_2 = \{\Sigma[w(F_o^2-F_c^2)^2]/\Sigma[w(F_o^2)^2]\}^{1/2}$

Responses to A level and B level check cif alerts for 2DWPN-2 single crystal

Alert level A

THETM01_ALERT_3_A The value of sine(theta_max)/wavelength is less than 0.550 calculated sin(theta_max)/wavelength = 0.5262

Response: The higher angle diffraction data was too weak for structural determination because of the low quality crystal that weakly diffracted.

PLAT029_ALERT_3_A Resolution (too) Low [sin(theta)/Lambda < 0.6]. 0.50 Ang^{-1} Why?

Response: The crystal was very weakly diffracting at high angles.

Alert level B

PLAT340_ALERT_3_B Low Bond Precision on C-C Bonds 0.01319 Ang. Why?

Response: This is due to the large number of weak reflections.

Supplementary Table 5| *Elastic moduli and hardness of* ***2DWPN-1***

	E (elastic modulus)<br>GPa	***H*** (hardness)<br>GPa
1	4.07	0.241
2	4.05	0.215
3	3.49	0.250
4	4.39	0.281
5	4.20	0.240
Average	**4.04**	**0.245**

Supplementary Table 6| *Elastic moduli and hardness of* ***NWPN-1***

	E (elastic modulus)<br>GPa	***H*** (hardness)<br>GPa
1	6.15	0.364
2	5.74	0.334
3	6.04	0.344
4	6.04	0.342
5	5.98	0.330
Average	**5.99**	**0.343**

Supplementary Table 7| *Calculated results of the dative B−N bond energy*

	**2DWPN-1**
Adduct	–1614.78305
**BDBB**	–5762.06191
**BPE**	–1042.66376
B−N bond energy	–0.0574 hartree<br>–150.673 kJ mol^{-1}
B−N bond length	1.70 Å

Supplementary References

1. Rambo B. M. & Lavigne J. J., Defining self-assembling linear oligo(dioxaborole)s. *Chem. Mater.* **19**, 3732–3739 (2007).
2. M.J. Frisch et al., Gaussian 09, Revision A.1, Gaussian, Inc., Wallingford CT, 2009.
3. Materials Studio; Accelrys: San Diego
